# The interaction of *Orthoflavivirus* nonstructural proteins 3 and 5 with human fatty acid synthase

**DOI:** 10.1371/journal.pone.0319207

**Published:** 2025-03-25

**Authors:** Suthatta Sornprasert, Wannapa Sornjai, Duncan R. Smith

**Affiliations:** Institute of Molecular Biosciences, Mahidol University, Salaya, Thailand.; Instituto Nacional de Salud Pública: Instituto Nacional de Salud Publica, MEXICO

## Abstract

Mosquito-transmitted viruses of the genus *Orthoflavivirus* impose a significant public health burden in many tropical and sub-tropical countries around the world, yet there is still no therapeutic drug to treat infection by any of these viruses, and so a deeper understanding of the mechanism of viral replication is required to identify potential therapeutic targets. Studies have shown that lipid metabolism is modulated upon virus infection, and that fatty acid synthase (FASN) is a key enzyme in fatty acid biosynthesis. In particular it has been reported that FASN interacts with DENV NS3 and is subsequently located to the replication complex. To further investigate this, the interaction of FASN with NS3 and NS5 of the Orthoflaviviruses dengue virus (DENV), Zika virus (ZIKV), and Japanese encephalitis virus (JEV) was investigated by coimmunoprecipitation and indirect immunofluorescent assay. Unexpectedly, FASN interacted with both NS3 and NS5 independently. The colocalization of NS3 and FASN was found for all investigated viruses, and while NS5 interacted with FASN, colocalization was not observed. Markedly however, FASN colocalized with dsRNA, a marker for the replication complex. FASN is an essential enzyme and plays a role in viral replication complex and cellular membrane remodelling. The interaction of FASN with both NS3 and NS5, as well as some of FASN being localized to the site of replication for DENV, JEV and ZIKV further highlights FASN as an important therapeutic target which may have applications to many mosquito-transmitted Orthoflaviviruses.

## Introduction

The genus *Orthoflavivirus* [1] in the family *Flaviviridae* contains 50 viral species which are predominantly transmitted via hematophagous arthropods, and which collectively pose a significant public health concern worldwide. In tropical and subtropical countries mosquito transmitted viruses such as dengue virus (DENV), West Nile virus (WNV), Zika virus (ZIKV), Japanese encephalitis virus (JEV) and yellow fever virus (YFV) exert a significant impact on public health [2].

The largest impact by these viruses on human health worldwide as measured by number of infections per year is caused by the four viruses of the species *Orthoflavivirus denguei* (DENV 1 to 4), which are endemic in over 100 tropical and subtropical countries around the world, and which cause an estimated 390 million human infections yearly [3]. The majority of human infections (80%) are asymptomatic, and in symptomatic cases, symptoms can range from a mild flu like fever to a severe and life-threatening hemorrhagic syndrome [4]. In contrast to the primary hemorrhagic manifestations of DENV infection, other viruses in this genus such as JEV and ZIKV primarily manifest neurological symptoms in severe cases [5]. Although there are highly effective vaccines to protect against JEV, it remains a leading cause of encephalitis in parts of Asia [6]. The recently emerged ZIKV [7] causes significant neurological and other damage to developing fetuses when a pregnant woman becomes infected during the first or second trimester of pregnancy [8].

The Orthoflavivirus genome consists of a single-stranded positive sense RNA of approximately 9.2-11 kb, which translates into a single polypeptide that is cleaved by host and viral proteases into three structural proteins (Capsid (C), pre-Membrane (prM/M) and Envelope (E), and seven of non-structural proteins (NS1, NS2A, NS2B, NS3, NS4A, NS4B, and NS5) [9]. Among the non-structural proteins, NS3 and NS5 both possess enzymatic activities that are essential in generating new genomes through RNA helicase (NS3) and RNA polymerase (NS5) activities [10]. These two proteins (in addition to other structural and non-structural proteins) are involved in processes that serve to modulate the cellular machinery to dampen the innate immune response and promote viral replication [11–14].

NS3 consists of 618 amino acid residues which are separated into two main domains. The N-terminal domain is the protease domain, while the C-terminal domain contains helicase, RNA-stimulated nucleoside triphosphatase (NTPase), and RNA-triphosphatase (RTPase) activities [10]. To be a functional protease, the N-terminal of NS3 requires interaction with 40 amino acids of the NS2B co-factor [15,16]. NS5 is the largest nonstructural protein of Orthoflaviviruses, consisting of approximately 900 amino acid residues. NS5 also consists of two domains and the sequence of NS5 is highly conserved across flaviviral species [17]. The N-terminal domain of NS5 contains methyl-transferase (MTase) activity responsible for cap formation, while the C-terminal domain possesses RNA-dependent RNA polymerase (RdRp) activity which plays a role in viral RNA replication [18]. During *Orthoflavivirus* RNA replication, NS3 and NS5 interact in the replication complex to synthesize the genome and to maintain the positive sense and negative sense strands of the viral RNA genome. The interaction between NS3 and NS5 occurs between the C-terminal region of NS3 between amino acid residues 566-585 and the N-terminal region of NS5 between amino acid residues 320-341. A competitive ELISA study and a biochemical assay identified two specific sites that affected the interaction, namely asparagine-570 of NS3 and the lysine-330 of NS5 [19,20]. In addition to interacting with each other, both NS3 and NS5 have been shown to interact with a large number of host cell proteins [21].

Fatty acid synthase (FASN) is the rate-limiting enzyme in *de novo* fatty acid synthesis. FASN is a 270 kDa, multifunctional enzyme, which synthesizes palmitic acid (C16) the precursor of the longer lipid chains from acetyl Co-A and malonyl Co-A [22]. FASN plays a role in DENV 2 replication in a human cell line as inhibition of FASN via siRNA markedly decreased DENV replication [23], while the FASN inhibitor orlistat has been shown to possess antiviral activity against DENV, ZIKV, JEV, and chikungunya virus [24].

In a previous study, DENV NS3 was shown to interact with FASN and to induce the re-localization of FASN to the DENV replication complex to support viral replication although infection did not increase overall FASN expression [25]. A more recent study suggested that the interaction of FASN with DENV NS3 was dependent upon on the conversion of the GDP-bound form of Rab18 to the GTP-bound form of Rab18, and that the targeting of FASN to the replication complex was dependent on functional Rab18 [26]. In another recent interactome study, we observed the interaction of ZIKV NS5 with FASN, but no interaction was observed between JEV NS5 and FASN [27].

Given that NS3 and NS5 interact [19,20], and that interactions have been variously reported between FASN and NS3 [25,26] or NS5 [27], it is unclear whether these represent separate interactions, or apparent interactions mediated by the NS3/NS5 interaction. This study therefore sought to determine for DENV, JEV and ZIKV whether FASN interacted with NS3 and/or NS5.

## Materials and methods

### Cell lines and viruses

The human embryonic kidney cell line HEK293T/17 (ATCC CRL-11268), the human lung adenocarcinoma cell line A549 (ATCC CCL-185) and the Rhesus monkey kidney epithelia cell line LLC-MK2 (ATCC CCL-7) were maintained in Dulbecco’s Modified Eagle’s Medium (DMEM, GIBCO, Invitrogen, Grand Island, NY) supplemented with 10% or 5% (respectively) fetal bovine serum (FBS, GIBCO, Invitrogen, Grand Island, NY) and 5% CO_2_ at 37°C. The *Aedes albopictus* cell line C6/36 (ATCC CR-1660) was maintained in Minimal Essential Medium (MEM, GIBCO, Invitrogen, Grand Island, NY) supplemented with 10% FBS at 28°C without CO_2_ supplementation. Viruses were DENV 2 (lab strain, 16681, NCBI Accession number NC_001474), ZIKV (Asian lineage, Thai strain, ZIKV SV0010/15), and JEV (Beijing strain, BJ1, NCBI accession number L48961). The viruses were propagated in C6/36 cells and titer determined by standard plaque assay as described previously [24]. The identity of the viruses was confirmed by commercial DNA sequencing (Macrogen, Seoul, Korea) before use.

### Recombinant NS3 and NS5 of DENV 2, ZIKV, and JEV

To construct recombinant NS3 of DENV, ZIKV, and JEV tagged with the EGFP, viral RNA was extracted from viral stocks using TRIzol reagent (Thermo Fisher Scientific, Waltham, MA) according to the manufacturer’s recommendations. RNA was reverse transcribed to cDNA using RevertAid Reverse Transcription kit (Thermo Fisher Scientific, Waltham, MA) according to the manufacturer’s recommendations. Full-length NS3 of DENV 2, ZIKV, and JEV were amplified using Phusion High-Fidelity DNA Polymerase (Thermo Fisher Scientific, Waltham, MA) under the following conditions: 98 °C for 30 sec, 35 cycles of 98 °C for 10 sec, 60 °C for 30 sec, 72 °C for 1.40 min and final extension at 72 °C for 10 min. The primer sequences are given in S1 Table. The NS3 fragments of all three viruses were inserted into the EGFP-C2 plasmid between the *Hin*dIII and *Kpn*I restriction enzyme sites using T4 DNA Ligase (5 U/ µ L). The recombinant plasmids were confirmed by commercial Sanger DNA sequencing (Macrogen, Seoul, Korea). Recombinant NS5 proteins of DENV, ZIKV and JEV tagged with EGFP were as previously described [27].

To construct recombinant FASN tagged with the EGFP, total RNA was extracted from HEK293T/17 cells using TRIzol reagent (Thermo Fisher Scientific, Waltham, MA) and cDNA was prepared as described above. Two fragments of FASN were generated from the cDNA using specific primers (C2-FASN-HindIII-F/ FASN-NheI-R and FASN-NheI-F/ C2-FASN-KpnI-R) and touch-down PCR, using the following conditions: 98 °C for 30 sec, 8 cycles of 98 °C for 10 sec, 68 °C to 61 °C for 30 sec (decrease 1°C every cycle), 72 °C for 3 min (first fragment), 5 min (second fragment), and 27 cycles of 98 °C for 10 sec, 60 °C for 30 sec 72 °C for 3 min (first fragment), 5 min (second fragment) and final extension at 72 °C for 10 min. The two FASN fragments generated were purified, double digested with *Hin*dIII plus *Nhe*I and *Nhe*I plus *Kpn*I and cloned into plasmid pEGFP-C2 between the *Hin*d III and *Kpn* I restriction sites. The recombinant plasmid was confirmed by commercial DNA sequencing (Macrogen, Seoul, Korea).

### Transfection and infection

HEK293T/17 cells were seeded at approximately 3 x 10^6^ cells on 10 cm dishes (for Co-IP) or at approximately 4 x 10^5^ cells on coverslips in 6-well plates (for immunofluorescence assay) one day before transfection to allow cells to reach approximately 40% confluency on the day of the experiment. A total of 2.5 μg or 15 μg of plasmid was transfected into the HEK293T/17 cells by the calcium phosphate transfection method. The cells were collected at 48 h post-transfection (h.p.t.) by scraping and washed with ice-cold phosphate buffered saline (PBS) or by fixing with 4% formaldehyde for IFA. In combination of transfection and infection experiment, the transfection complex was removed from the cell at 24 h.p.t, after which DENV, ZIKV or JEV as appropriate diluted in serum-free culture media with an appropriate viral titer was added to the cells for 2 hours. After this time the medium was removed and replaced with complete medium and cells were incubated for a further 24 hours (for DENV and ZIKV) or 12 hours (for JEV) before collecting the cells.

### Co-immunoprecipitation (Co-IP) and western blot analysis

Proteins were extracted from cells by incubating them in Co-IP lysis buffer (10 mM Tris/Cl pH7.5, 150 mM NaCl, 0.5 mM EDTA, 0.5% of Nonidet P40 Substitute (Merck & Co., Rahway, NJ)) supplemented with 1X PIC (100X Protease inhibitor cocktail, Biobasic Inc, Ontario, Canada) and 1 mM PMSF. Briefly, 200 µl of lysis buffer was applied to the collected cells and samples were vortexed on ice for 30 min before centrifuging at 12,000 g for 10 min. The supernatant was collected, and then 300 µl of diluent buffer (10 mM Tris/Cl pH7.5, 450 mM NaCl, 0.5 mM EDTA) supplemented with 1X PIC and 1 mM PMSF was added. After that 20 µl of the protein was collected for determining the input protein lysate by western blot analysis. GFP-trap Agarose beads (ChromoTek, Planegg, Germany) were added to the remaining protein solution and the mixture was rotary mixed at 4°C for 2 hours. The complex of protein-bound with GFP-trap Agarose beads were washed 10 times with wash buffer (10 mM Tris/Cl pH7.5, 450 mM NaCl, 0.5 mM EDTA, 0.05% of Nonidet P40 Substitute, Triton X-100 0.05%) at 4°C to remove unbound proteins. The bound proteins were eluted from the GFP beads by addition of 2.5x SDS buffer with DTT and incubation at 90°C, for 15 min before collecting the eluate using a needle.

Proteins were separated by electrophoresis through 10% SDS-PAGE gels and proteins were transferred onto nitrocellulose membranes (Whatman GmbH, Germany) using a wet blotting transfer system (Bio-Rad Laboratories, Richmond, CA) for 3 hours. After that, 5% skimmed milk (w/v) in TBS-T (Tris-buffer saline plus 0.05% Tween20) was used to block the membrane before applying primary antibodies for FASN (dilution 1:1000; sc-32233), GFP (dilution 1:3000; sc-8334), GAPDH (dilution 1:10000; sc-55580), HSP90 (dilution 1:5000; sc-7947), GRP78 (dilution 1:3000; sc-166490) and DENV-ENV (dilution 1:10,000; MA1-27093) ZIKV-ENV (dilution 1:5000; GTX133314). For reverse Co-IP the primary antibodies were for DENV-NS3 (dilution 1:5000; GTX124252), DENV-NS5 (dilution 1:3000; MA517295), ZIKV-NS3 (dilution 1:5000; GTX133309), ZIKV-NS5 (dilution 1:5000; GTX133312), JEV-NS3 (dilution 1:10000; GTX125868), JEV-NS5 (dilution 1:5000; GTX131359) and actin-HRP (dilution 1:50,000; sc-9996). After incubation with primary antibodies overnight at 4°C the membranes were washed three times with TBS-T before incubation with an appropriate secondary antibody at room temperature for 1 h, which were a HRP conjugated rabbit anti-mouse IgG (dilution 1:5000; A9044, Merck KGaA, Darmstadt, Germany), and a HRP conjugated goat anti-rabbit IgG (dilution 1:5000; 31460, Pierce, Rockford, IL). Following secondary incubation, the membranes were washed three times with TBS-T, and the signals were developed by using Immobilon Forte Western blot HRP Substrate (Merck KGaA, Darmstadt, Germany) and detected immediately on X-ray film. The antibody codes “GTX, SC, MA5” indicate the antibodies were purchased from GeneTex, Irvine, CA., Santa Cruz Biotechnology, Dallas, TX, USA, and Thermo Fisher Scientific, Waltham, MA, respectively.

### Indirect immunofluorescence assay (IFA)

HEK293T/17 cells grown on coverslips were fixed with 4% paraformaldehyde for 20 minutes and permeabilized by addition of 0.3% of Triton-x in 1X PBS buffer for 10 min. After that cells were blocked with 10% of normal goat serum before incubation with the primary antibody; DENV-NS3 (dilution 1:50; GTX124252), DENV-NS5 (dilution 1:50; PA527888), ZIKV-NS3 (dilution 1:50; GTX133309), JEV-NS3 (dilution 1:10,000; GTX131359), FASN (dilution 1:25; sc-32233), FASN (dilution 1:50; 3180) and J2 (dilution 1:150; J2-0702, Scicons, Limburg, Netherlands) at 4°C in a humid chamber overnight. After washing cells were then incubated with an appropriate secondary antibody, donkey anti-mouse IgG antibody conjugated with Alexa^TM^ Fluor 488 (dilution 1:100; PA5-21202), donkey anti-rabbit IgG antibody conjugated with Alexa^TM^ Fluor 647 (dilution 1:100; PA5-31573) and 0.5 µg/ml DAPI for one hour. After washing cell were mounted on glass slides in, ProLong Gold Antifade reagent (Thermo Fisher Scientific, Waltham, MA) before observation under a confocal laser scanning microscope LSM 800w Airy scan (ZEISS, Oberkochen, Germany) at 63x magnification.

### Colocalization analysis

The co-localization analysis was performed using ZEN blue with 2-dimensional analysis of colocalization, and ImageJ software [28] with the PSC colocalization plugin [29]. GFP-C2 and MOCK samples were used for setting the threshold in both channels (GFP or Alexa^TM^ Fluor 488 and Alexa^TM^ Fluor 647). Because not every cell was transfected or infected, target cells which presented both colors were individually selected for analysis of the colocalization with a minimum of ROIs n = 20. The statistical analysis of colocalization was undertaken by determining the Pearson’s correlation coefficient (-1, + 1). Non-parametric unpaired student’s T-test and one-way ANOVA were used to determine differences in correlation coefficient between samples using GraphPad Prism version 8 for Windows (GraphPad Software, La Jolla, CA). The statistically significant two-tailed P-value are displayed as *  (P ≤  0.05), ** (P ≤  0.01), and *** (P ≤  0.001).

To undertake a 3-dimensional analysis of co-localization analysis approximately 30 stack photos were collected at 63X magnification with zoom in 1.5 times using the ZEN blue program. The percent colocalization was analyzed by the object base method (Imaris version 9.9.0). The criteria of each object were set as a sphere shape with a 1.00 radius scale. The algorithm settings for analysis were classified as spot and object-to-object statistics. For spot detection, the estimated XY diameter of all samples was set as 0.5 µ M with background subtraction. The object was classified into two groups including colocalization and non-colocalization. The criteria cut off was set as 0.5 µ M for the shortest distance to spot-spot.

## Results

### Human FASN and the viral protein NS3 and NS5 of DENV, ZIKV, and JEV interaction

**Fig 1:**
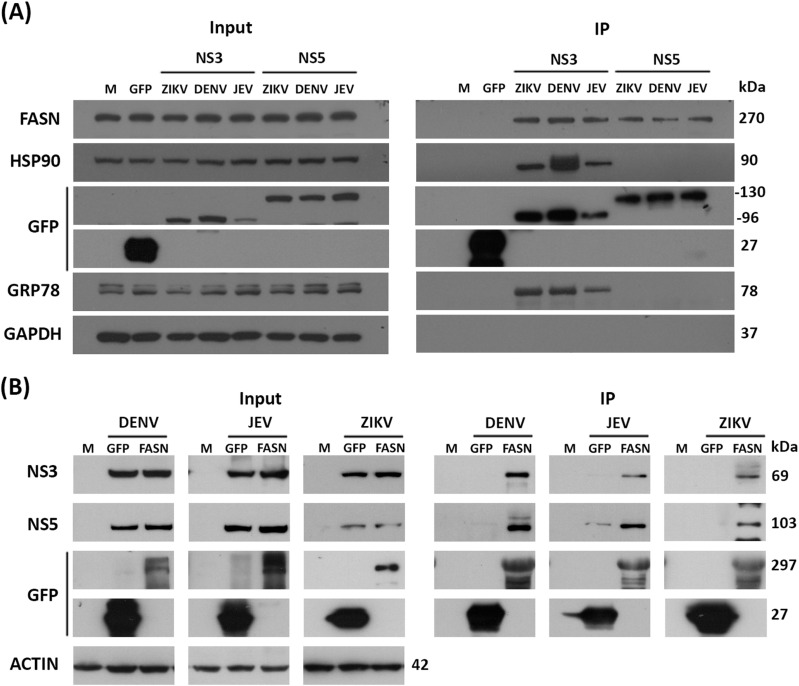
The interaction of FASN with NS3 and NS5 of DENV, ZIKV, and JEV. (A) Co-IP, HEK293T/17 cells were transfected with the recombinant NS3 and NS5 of DENV, JEV and ZIKV. The expression level of recombinant protein after 2 days post-transfection was detected by probe with the anti-GFP and other proteins were detected with the specific antibodies (Input; left). The Co-IP was performed by using the GFP trapped bead to target the GFP tagged with the bait proteins (NS3 and NS5). The target prey protein (FASN) and the positive control (the known interactors) were probed with the specific antibodies to FASN, HSP90, and GRP78 (IP; right). (B) Reverse Co-IP, HEK293T/17 cells were transfected with the recombinant FASN and infected with DENV, JEV, and ZIKV. The expression level of recombinant protein FASN were detected by probe with the anti-GFP and other proteins were detected with the specific antibodies (Input; left). Reverse co-IP was performed by using the GFP trapped bead to target the GFP tagged with the bait proteins (FASN). The prey proteins (NS5 and NS3) were probed with the specific antibodies to NS5 and NS3 of each virus (IP; right). An anti-GFP antibody was used to check the level of proteins which were immunoprecipitated (IP; right). All experiments were undertaken as independent biological triplicates. Composite images are shown consisting of successive antibody probings of the same membrane which are separated by white bars. Full, uncropped western blots can be found in the supplemental materials.

To determine any interactions between FASN and NS5 and NS3 of DENV, ZIKV, and JEV, recombinant plasmids of the appropriate Orthoflaviviral protein in frame with EGFP were either constructed (NS3) or were as previously described (NS5, [27]). HEK293T/17 cells were transfected individually with the six constructs, and on day 2 post-transfection cells were collected and proteins prepared. A portion of the protein lysate was reserved (input), while the rest was used to pull down the Orthoflaviviral proteins using a GFP-trap. Western blot analysis clearly showed that FASN was pulled down by all six constructs (Fig 1A). We additionally probed the pulled down proteins for the presence of chaperone proteins known, or likely to interact with either NS3 and or NS5 including GRP78 and HSP90 [30,31]. Both GRP78 and Hsp90 interacted with all three NS3 constructs (Fig 1A), but not with the NS5 constructs. Previously we had reported that Hsp90 interacted with both DENV NS3 and NS5 [30]. However, that study was undertaken using virus infection (and not with cloned proteins), and so it seems likely that Hsp90 was shown as an interactor of NS5 through the well-known NS3-NS5 interaction.

To confirm the interactions, reverse Co-IP was undertaken using FASN as the bait protein to identify NS3 and NS5 viral prey proteins. A recombinant FASN tagged with EGFP was constructed and transfected into the HEK293T/17 cells. At 24 hours post transfection, the cells were separately infected with DENV, ZIKV, and JEV. Proteins were collected at 24 hours post infection for DENV and ZIKV, and at 12 hours post infection for JEV because both NS3 and NS5 are highly expressed at 12 h.p.i. [32]. GFP-trap beads were used to pull down FASN, and the presence of NS3 or NS5 was determined in the immunoprecipitated proteins. The results (Fig 1B) confirmed the previous results, in that both NS3 and NS5 for the three viruses were pulled down together with FASN. It should however be noted that a signal was observed for the JEV GFP negative control, both in input and IP, the cause of which is unclear. Neither DENV or ZIKV had a similar signal in the IP, but did have a signal in the input proteins.

### Independent interactions of NS3 and NS5 of DENV, ZIKV, and JEV with FASN

**Fig 2:**
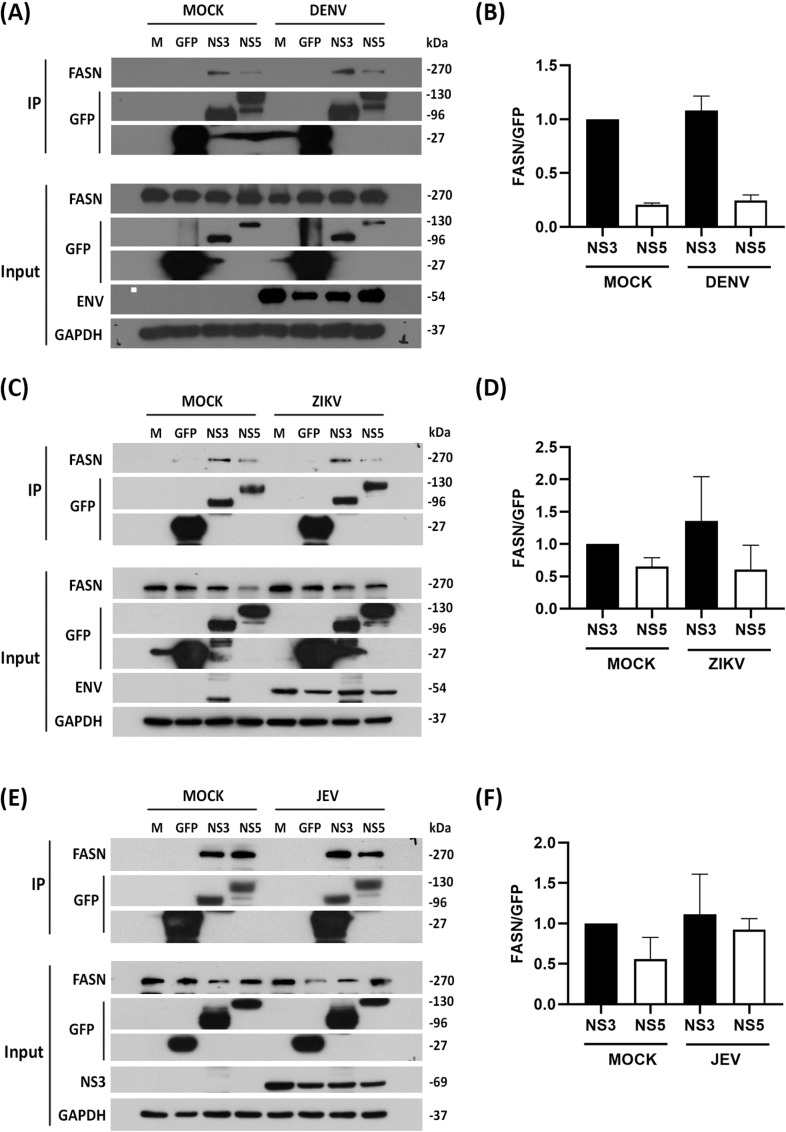
The interaction of NS3 and NS5 with FASN during DENV, ZIKV, and JEV infection. HEK293T/17 cells were transfected with the recombinant NS3 and NS5 of DENV, ZIKV, and JEV for 24 h before infected with DENV, ZIKV, and JEV MOI 5. The cell lysates were collected after 24 h (for DENV and ZIKV) or 12 h (for JEV) post-infection. To determine the level of expression before co-IP, the expression level of recombinant protein NS3 and NS5 of DENV, ZIKV, and JEV were detected by probing with the anti-GFP antibody. The Co-IP was performed by using the GFP trapped bead to target the GFP tagged with the bait proteins (NS3 and NS5 of DENV, ZIKV, and JEV; A, C, E, respectively). The target prey protein (FASN) was detected with an anti-FASN antibody (A, C, E). Anti-GFP was probed to check the level of proteins which can immunoprecipitated. Western blot quantification between FASN prey protein and GFP immunoprecipitated protein of (B) DENV, (D) ZIKV, and (F) JEV. Experiments were all undertaken as independent biological triplicate. Composite images are shown consisting of successive antibody probings of the same membrane which are separated by white bars. Full, uncropped western blots can be found in the supplemental materials.

The previous results showed that both NS3 and NS5 interact independently with FASN. To determine if the interaction is enhanced in the presence of the other protein, DENV, ZIKV, and JEV NS3 and NS5 were separately transfected into HEK293T/17 cells, and after 24 hours the cells were either infected with DENV, ZIKV, JEV, or not infected. After a further 24 h (for DENV, ZIKV), or 12 h (JEV) proteins were collected and NS3 and NS5 were pulled down using a GFP-trap, after which the immunoprecipitated proteins were probed to detect the presence of FASN (Fig 2A, C, E). The results (Fig 2B, D, F) showed no significant difference in FASN signal intensity between the transfection/mock infection and transfection/DENV, ZIKV, JEV infection samples.

### Localization of recombinant NS3 and NS5 of DENV, ZIKV, and JEV

**Fig 3:**
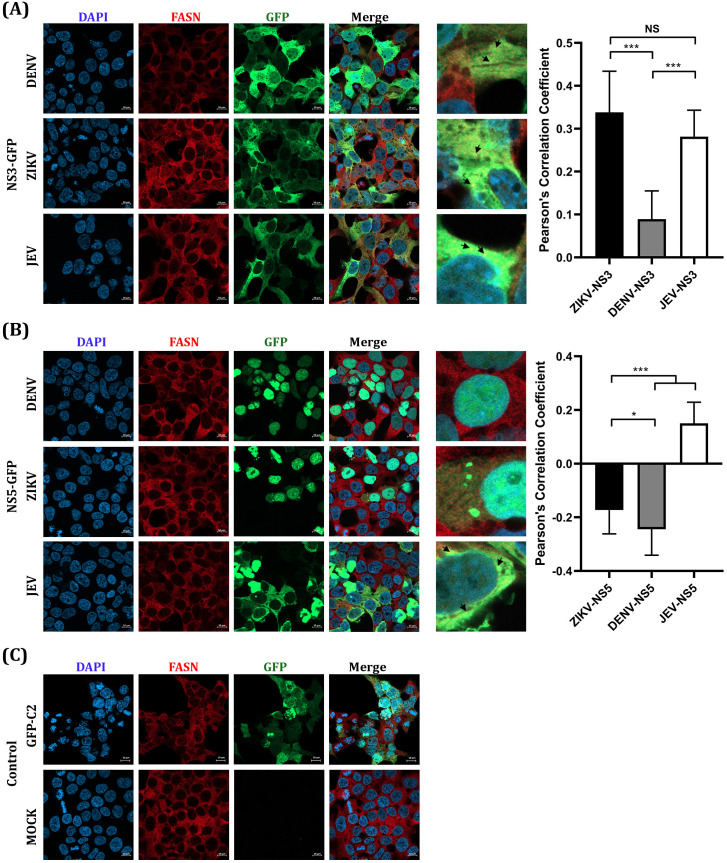
The localization of FASN and recombinant NS3 and NS5 proteins of DENV, ZIKV, and JEV tagged with EGFP. HEK293T/17 cells were transfected with the recombinant NS3 and NS5 of DENV, ZIKV and JEV on the cover slips. At 2-day post-transfection, cells on cover slips were examined by IFA with the primary antibody to FASN and secondary antibody Alexa^TM^ Fluor 647 (red). The localization of each protein was observed with 63X magnification by confocal microscopy. DAPI was used to stain nucleus (blue). GFP represented the location of (A) NS3 and (B) NS5 (C) GFP-C2 empty vector (green). The statistical analysis of Pearson’s correlation is shown in the right panel. NS means non-significant (P >  0.05), *  (P ≤  0.05) and *** (P ≤  0.001).

To determine the location of the EGFP-tagged NS3 and NS5 in transfected cells, transfected cells were stained to detect GFP and FASN and observed using a confocal microscope. EGFP-NS3 was mostly found in the cytoplasm, while EGFP-NS5 of DENV and ZIKV was found in the nucleus, while JEV EGFP-NS5 showed a cytoplasmic localization (Fig 3). FASN also showed a cytoplasmic localization (Fig 3).

To evaluate the degree of colocalization between NS3 and NS5 with FASN, the colocalization with FASN was observed by staining the cell with an antibody to FASN. The colocalization was evaluated by a 2-dimensional analysis of the Pearson’s correlation coefficient which showed the colocalization of NS3 and FASN differed significantly among the viruses (Fig 3A, right panel). The colocalization was observed in some regions which is shown in the zoom images (Fig 3A) and indicated with black arrows. On the contrary, no colocalization was presented between NS5 and FASN in ZIKV and DENV as shown by the negative value of the Pearson’s coefficient value and by observation. The colocalization of NS5 and FASN was found with JEV (Fig 3B, left panel). In sum, FASN colocalized in some areas with NS3 protein of all three viruses at the different levels in concordance with the co-immunoprecipitation results.

### The rearrangement of the correlation between FASN and viral proteins NS3 and NS5 of DENV, ZIKV, and JEV upon natural infection.

**Fig 4:**
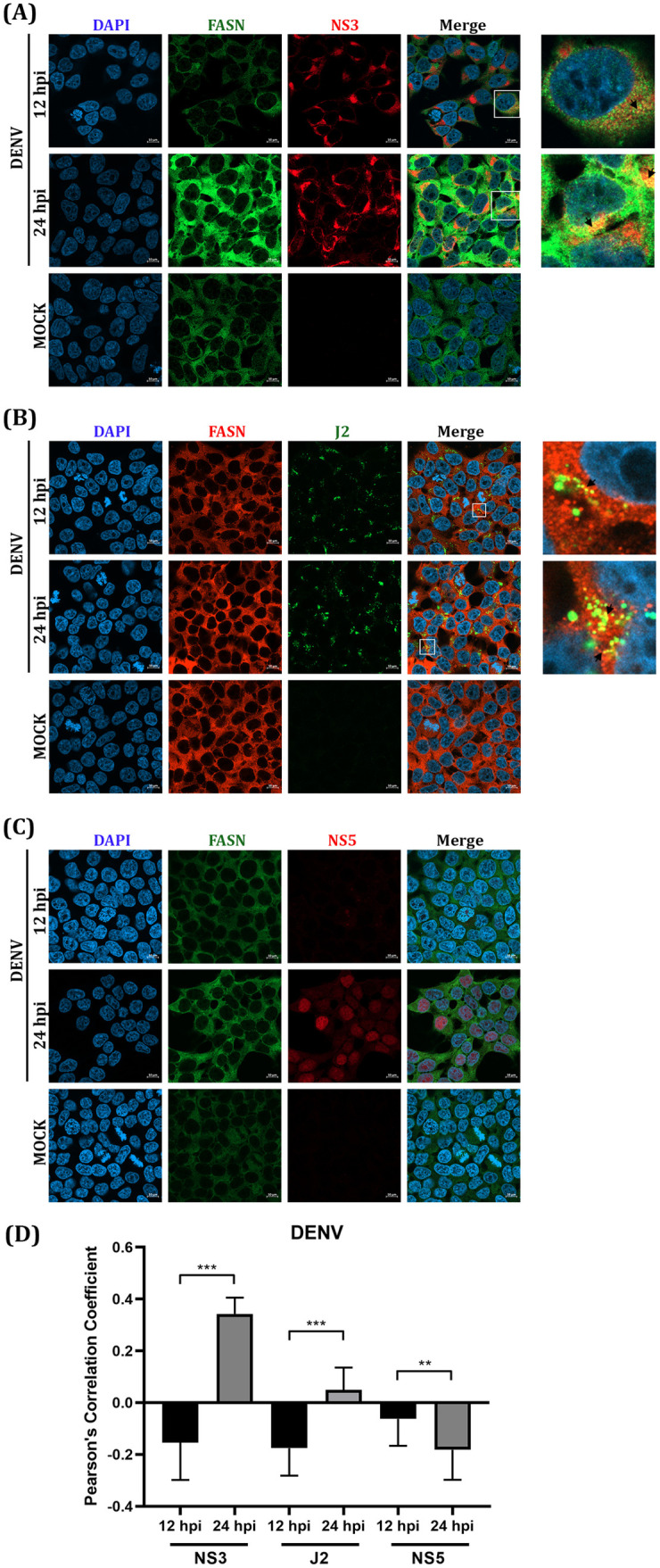
Colocalization analysis of FASN and DENV viral proteins NS3 and NS5 and dsRNA. HEK293T/17 cells on cover slips were infected with DENV MOI 5. After 12 and 24 hpi, cells were used in an IFA analysis by double staining with the specific primary antibodies to (A) FASN (green) and NS3 (red) (B) FASN (red) and dsRNA (green) (C) FASN (green) and NS5 (red) and the secondary antibodies (Alexa^TM^ Fluor 488 and Alexa^TM^ Fluor 647). DAPI was used to stain the nucleus (blue). The localization of each protein was observed with 63X magnification by confocal microscopy. The statistical analysis of Pearson’s correlation is shown in panel (D). ** (P ≤  0.01), and *** (P ≤  0.001).

**Fig 5:**
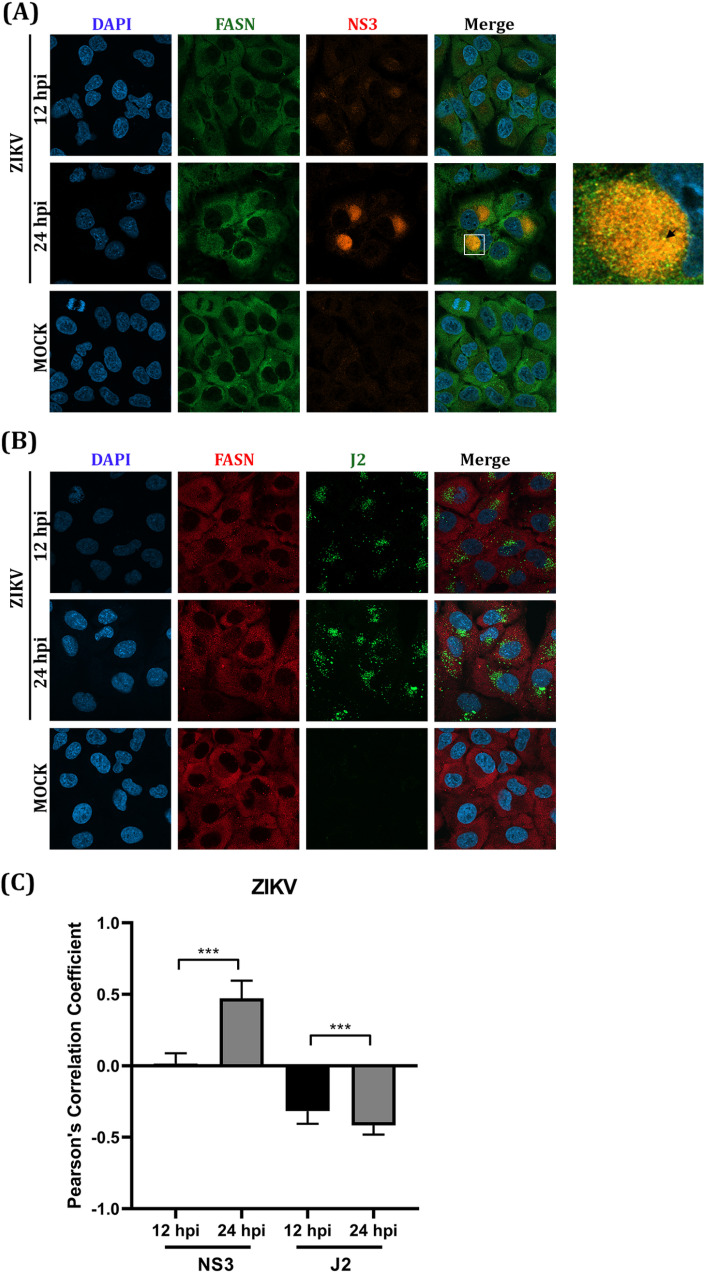
Colocalization analysis of FASN and ZIKV protein NS3 and dsRNA. A549 cells on cover slips were infected with ZIKV MOI 5. After 12 and 24 hpi, cells on cover slips were used in an IFA analysis by double staining with the specific primary antibodies to (A) FASN (green) and NS3 (red) (B) FASN (red) and dsRNA (green) and the secondary antibodies (Alexa^TM^ Fluor 488 and Alexa^TM^ Fluor 647). DAPI was used to stain the nucleus (blue). The localization of each protein was observed with 63X magnification by confocal microscopy. The statistical analysis of Pearson’s correlation is shown in panel (C). *** (P ≤  0.001).

**Fig 6:**
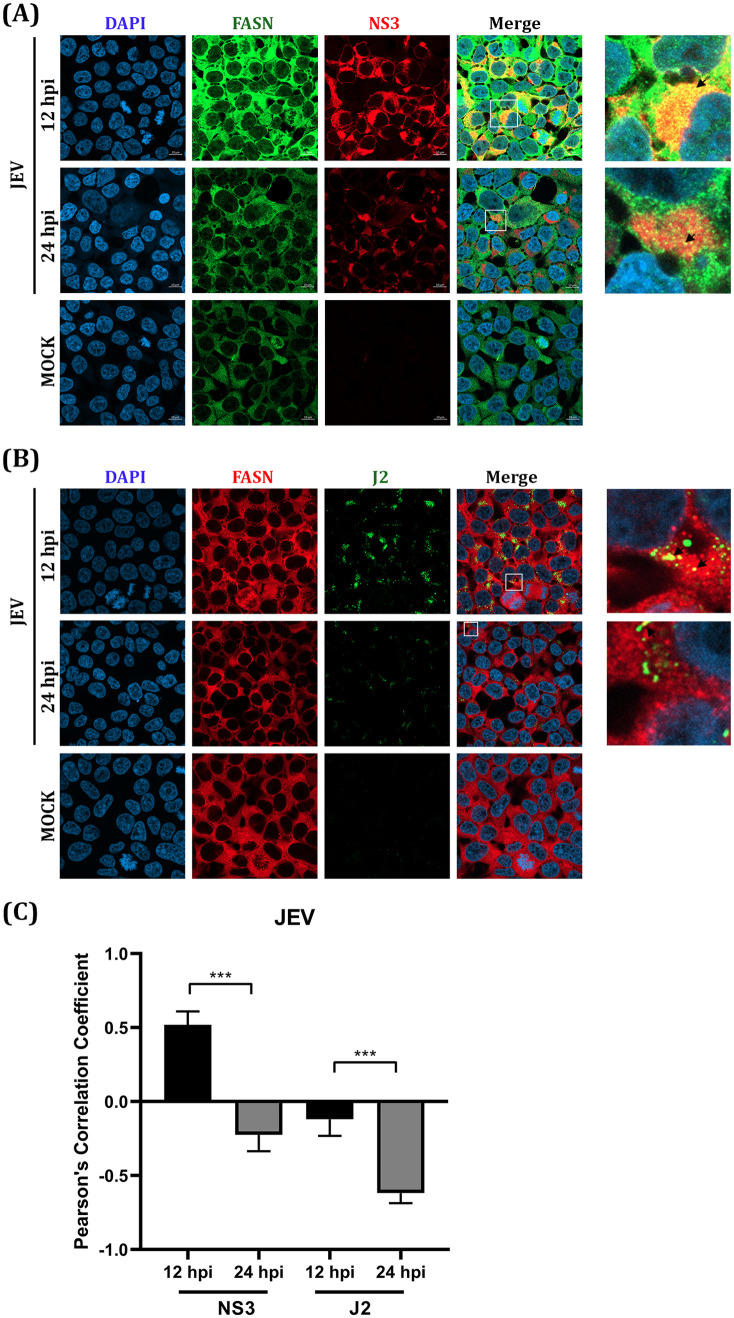
Colocalization analysis of FASN and JEV protein NS3 and dsRNA. HEK293T/17 cells on cover slips were infected with JEV MOI 5. After 12 and 24 hpi, cells on cover slips were used in an IFA analysis by double staining with the specific primary antibodies to (A) FASN (green) and NS3 (red) (B) FASN (red) and dsRNA (green) and the secondary antibodies (Alexa^TM^ Fluor 488 and Alexa^TM^ Fluor 647). DAPI was used to stain the nucleus (blue). The localization of each protein was observed with 63X magnification by confocal microscopy. The statistical analysis of Pearson’s correlation is shown in panel (C). *** (P ≤  0.001).

The colocalization between FASN and the cloned viral proteins (NS3 and NS5) in transfected cells might not reflect the reorganization of the host and viral proteins upon virus infection, where ancillary proteins may play a significant role. To observe the rearrangement of FASN, NS3, and NS5 proteins in natural infection, DENV and JEV at MOI 5 were used to infect HEK293/17 cells, while ZIKV at MOI 5 was used to infect A549 cells. The location of each protein was observed at different time points (12 h and 24 h) under a confocal microscope. To indicate the specific location of the replication complex, the antibody J2 was used to determine the location of dsRNA. Colocalization of NS3 of all three viruses and FASN was observed at 24 h for DENV and ZIKV, and 12 h for JEV (Figs 4A, 5A, 6A). Colocalization of DENV NS5 was not observed, and expression of DENV NS5 was almost exclusively confined to the nucleus (Fig 4C). Due to the lack of suitable antibodies, localization of ZIKV and JEV NS5 could not be undertaken. FASN was shown to colocalize with DENV dsRNA at 24 h.p.i. (Fig 4B), but no colocalization was conserved at 12 h.p.i. (Fig 4B). The site of colocalization was predominantly perinuclear (Fig 4B). Similarly, perinuclear colocalization was observed between ZIKV NS3 and FASN (Fig 5A) at 24 h.p.i., but no colocalization was seen between FASN and ZIKV dsRNA (Fig 5B). For JEV, colocalization between NS3 and FASN was observed at 12 h.p.i, but not at 24 h.p.i (Fig 6A). Similarly, no colocalization was observed between FASN and dsRNA (Fig 6B). In each case, the degree of colocalization was assessed by analysis of the Pearson’s correlation coefficient and the correlation of either NS3, NS5 and dsRNA with FASN was significantly changed between 12 and 24 h.p.i for all three viruses (Fig 4D, 5C, 6C), confirming that the colocalization of FASN and viral proteins NS3, NS5, and dsRNA was altered during the viral infection for all three viruses at different time points.

The Pearson correlation coefficient was determined based on the intensity base colocalization of a 2-dimensional image that might be less accurate through accidentally merging in the same plain. Moreover, the correlation was calculated based on the two intensities of the channels, and it can be difficult to compare between viruses if the molecules under investigation have different cellular distributions, according to which virus is investigated. Therefore, the colocalization between dsRNA and NS3 was confirmed by an object base colocalization analysis which measured the proximity between two objects. The percentage of colocalization was calculated based on the percentage of viral NS3 protein or dsRNA colocalizing with FASN. The results showed that approximately 25-50% of NS3 and dsRNA colocalized with FASN (S1 Figs S1A, S1B, S1), confirming that both NS3 and dsRNA of DENV, ZIKV and JEV are colocalized with FASN.

### FASN expression in DENV, ZIKV, and JEV infected cells

**Fig 7:**
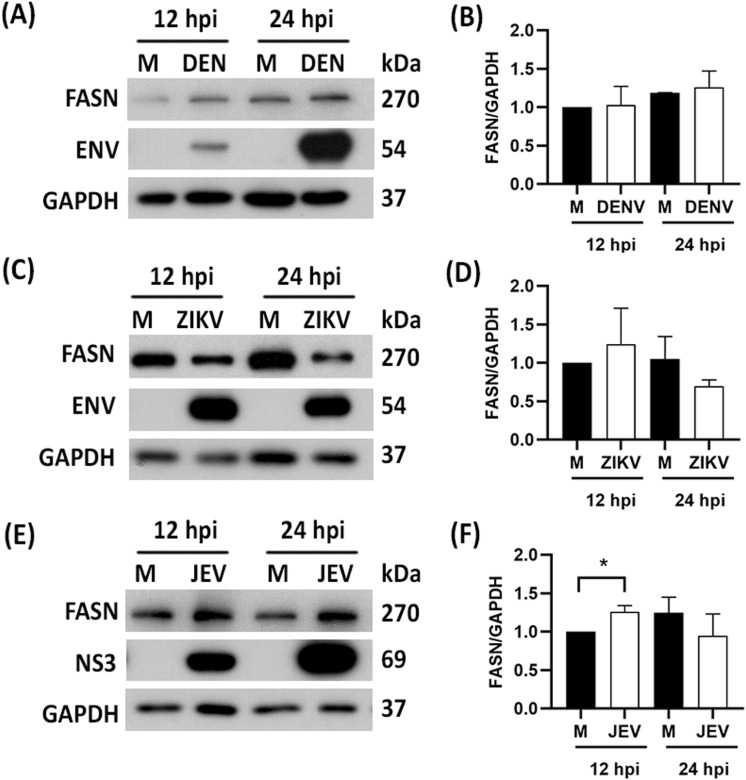
Expression of FASN in response to DENV and JEV infection of HEK293T/17 cells and ZIKV infected A594 cells. HEK293T/17 cells were infected with DENV and JEV at MOI 5 while A549 cells were infected with ZIKV MOI 5. The cells were collected at 12 and 24 h.p.i, and FASN expression was determined by western blot analysis probing with antibodies specific to FASN, viral protein ENV for (A) DENV, (C) ZIKV and (E) NS3 for JEV. All experiments were undertaken as three independent biological replicates. FASN expression levels in cells infected with the three viruses (B, D, F) were quantitated by the band intensity of FASN and an internal control, GAPDH. * (P >  0.05). Composite images are shown consisting of successive antibody probings of the same membrane which are separated by white bars. Full, uncropped western blots can be found in the supplemental materials.

To investigate FASN expression during virus infection HEK293T/17 cells (for DENV and JEV), or A549 cells (for ZIKV) were infected and the cells were collected after 12 and 24 hpi, the level of FASN protein expression was determined by western blot analysis. Cells infected with DENV and ZIKV showed no significant difference in FASN expression as compared to uninfected cells (Fig 7A-D), while there was a small but significant increase in cells infected with JEV at 12 hpi as compared to uninfected cells (Fig 7E, F). However, by 2 4h.p.i. this increase in FASN was no longer present.

## Discussion

Several of the mosquito-transmitted viruses of the genus *Orthoflavivirus* exert a significant impact on tropical and sub-tropical countries around the world [2]. Five viruses in this genus are on the World Health Organization “Pathogens prioritization” list (available at https://www.who.int/publications/m/item/pathogens-prioritization-a-scientific-framework-for-epidemic-and-pandemic-research-preparedness) as either a priority pathogen or a prototype pathogen, namely ZIKV, DENV, YFV, JEV and WNV. Three of these viruses (DENV, ZIKV and JEV) were investigated in this study. It should be noted that these viruses have no specific treatment option (above general supportive care), but that three (JEV, YFV and DENV) have commercial vaccines available. There are safe and highly protective vaccines for JEV and YFV [33], and two vaccines commercially available for DENV. However, the first vaccine, Dengvaxia, has been withdrawn from several markets due to cases of serious disease when the vaccine has been administered to a DENV naïve person [34,35]. The second DENV vaccine recently available (QDENGA (TAK-003)) is still under close monitoring, and some studies have suggested that seronegative children were only protected against DENV 1 and 2 [36], which again could significantly impact roll out of this vaccine. It is also important to note that vaccines provide very specific protection against a single virus species, and that there are a number of members of the genus *Orthoflavivirus* that have the potential to rapidly emerge [37], akin to what happened with the rapid emergence of ZIKV [7]. Thus, a good pan-Orthoflaviviral drug could serve as the first defense in the emergence of a new mosquito-transmitted *Orthoflavivirus*.

Drug development can develop along several pathways, such as off-target screening, de novo development or through developing drugs to specific targets [38]. For the latter option, it is necessary to understand the basic processes by which a virus interacts with and modulates the host cell to achieve optimization of virus production over the detriment to the cell itself. Numerous studies have shown that Orthoflaviruses modulate numerous (if not most) basic cellular mechanisms. One cellular process that has garnered interest is the modulation of the host cell lipid profile and several studies have pointed to lipid involvement at multiple stages in the viral replication cycle (reviewed in [39,40]). Interestingly, it has been proposed that a major consequence of lipid remodeling is alteration of the lipid composition of cellular membranes, to allow the flexibility for the lipid membrane carrying prM and E proteins to wrap around the nucleocapsid core [25]. These *in vitro* studies have been supported by studies undertaken on samples from DENV patients which have shown that there are distinct changes in the lipidome of DENV infected patients [41,42].

Autophagy is a cellular pathway involved in the lysosomal degradation of macromolecules and organelles, allowing recycling of the constituents [43]. Autophagy has been shown to be increased during DENV infection [44,45], and it has been proposed that the main consequence of this is to increase β-oxidation to increase the available energy to support DENV replication [46].

Lipid metabolism is broadly divided into three processes, namely lipogenesis, lipolysis and fatty acid oxidation and expression of genes in all three processes have been shown to be perturbed in DENV infection [23]. A key enzyme in lipid metabolism is FASN which is the rate limiting enzyme in the lipogenesis pathway, which catalyzes the synthesis of palmitate from the precursors acetyl-CoA and malonyl-CoA [47]. Several previous studies have highlighted the critical involvement of FASN in DENV infection. One previous study showed that inhibition of FASN through either siRNA knockdown or treatment with the drug orlistat significantly reduced viral replication for DENV, JEV and ZIKV [24]. Other studies have shown that lipid remodeling is associated with the re-localization of FASN to the replication complex by interaction with DENV NS3 [25], possibly through the involvement of Rab18 [26]. In our earlier study we also observed colocalization between DENV NS3 and FASN, but were unable to confirm the interaction between the two proteins in pull-down assays [23]. More recently we observed in a ZIKV and JEV NS5 interactome study that FASN was pulled down by ZIKV NS5 [27]. This suggests that both NS3 and NS5 have the potential to interact with FASN. However, there is also the possibility that FASN was pulled down by JEV NS5 through its interaction with NS3 which interacts with NS5 [19,20]. However, the earlier study was focused on cellular proteins that interacted with JEV and ZIKV NS5s, and did not investigate any flaviviral proteins co-immunoprecipitating with NS5. However, the well documented interaction between NS3 and NS5 [19,20] suggest that this could have been a possibility. In addition, the involvement of other host cell proteins mediating the interactions cannot be rules out.

In this study, we showed conclusively that FASN can interact independently with both NS3 and NS5 of DENV, JEV and ZIKV suggesting that this is probably a common process with other *Orthoflavivirus* members, we also showed that having both NS3 and NS5 present did not result in any increase in binding to FASN. We also note that our results for DENV agree with the study of Heaton and colleagues [25] who first observed the interaction between DENV NS3 and FASN, and is in contradiction to our previous observation [23]. It should be noted that this study was undertaken with cloned NS3, while our previous study was undertaken with natural infection which may account for the discrepancy. However, both studies have reported the colocalization of DENV NS3 and FASN [23,25].

While specific antibodies against ZIKV and JEV NS5 proteins were able to detect the proteins in western blot analyses, they gave no signal in IFA studies, suggesting these antibodies may have been raised against a conformation susceptible epitope. The antibody against DENV NS5 protein did give a signal, but the signal was almost entirely located in the nucleus, as has previously been noted by others [48,49], and no signal colocalizing with FASN was observed. The nuclear localization of DENV NS5 has been reported to be serotype specific, although studies do not necessarily agree on which serotypes predominantly localize to the nucleus, and which do not [50,51]. While NS5 protein must be part of the replication complex (as the RdRp), detection of its cytoplasmic localization remains largely enigmatic. With the EGFP- tagged constructs, no colocalization was observed between ZIKV-NS5 and FASN or between DENV-NS5 and FASN (consistent with the results seen in natural infection). A significant degree of colocalization was however observed between JEV-NS5 and FASN, and this reflects the different cellular locations of the proteins. NS5 of JEV can be found in the cytoplasm, but more than 80-90% of DENV NS5 after 24 post-infection is located in the nucleus [49].

The localization of NS5 in natural infection was determined in this study. However, it showed no colocalization in DENV, even at 12- and 24-hour post-infection. We suggested that the interaction of NS5 was observed in the Co-IP experiment due to the bead concentrating all expressed NS5 proteins in the cells to bind with GFP bead which has very high sensitivity. In contrast with IFA which focused on some areas in the cells. It should be noted that the ability of NS5 to interact with FASN is independent of any colocalization, and there may be brief or transitory points in the replication cycle where the interaction has functional merit. Further study for the real-time live cell imaging which is able to track the location at each time point might be help in unravelling the complexities of NS5 localization during infection.

The antibody J2 recognizes double stranded RNA and has been used previously as a marker for the DENV replication complex [45,52]. We observed colocalization between dsRNA and FASN at 24 hours post infection in DENV infection but failed to see it in ZIKV and JEV infection in a single plain analysis. However, in a Z-stack analysis 25-50% of NS3 and dsRNA colocalized with FASN for all three viruses confirming the localization of FASN at the replication complex.

This study has shown that both NS3 and NS5 are able to interact with FASN and that some FASN becomes relocated to the replication complex. Importantly, this was shown for three different mosquito-transmitted viruses, namely DENV, JEV and ZIKV, implying that this may represent a common mechanism for mosquito-transmitted members of the genus *Orthoflavivirus*.

## Supporting information

S1 File **S1 Fig.** The three-dimensional structure of HEK239/17 and A549 cells infected with DENV, JEV, and ZIKV at 12 and 24 hpi. Confocal microscopy determined the colocalization between FASN and NS3/dsRNA of (A) DENV (B) ZIKV (C) JEV. The images were taken in approximately 30 stacks, with 63X magnification and 1.5X Zoom. Each color represents different fluorochrome staining proteins. For staining of FASN and NS3, FASN is represented in green (AlexaTM Fluor 488), and NS3 is represented in red (AlexaTM Fluor 647). For staining of FASN and dsRNA, FASN is represented in red (AlexaTM Fluor 647), and NS3 is represented in green (AlexaTM Fluor 488). The nucleus is represented in blue (DAPI). The percent colocalization was analyzed by Imaris program (version 9.9.0) shown in the right panels. **S1 Table.** Primer sequences for constructing NS3 and FASN. **uncropped western blots.**
(PDF)

## References

[1] Postler TS, Beer M, Blitvich BJ, Bukh J, de Lamballerie X, Drexler JF, et al. Renaming of the genus Flavivirus to Orthoflavivirus and extension of binomial species names within the family Flaviviridae. Arch Virol. 2023;168(9):224. doi: 10.1007/s00705-023-05835-1 . 37561168

[2] Pierson TC, Diamond MS. The continued threat of emerging flaviviruses. Nat Microbiol. 2020;5(6):796–812. doi: 10.1038/s41564-020-0714-0 32367055 PMC7696730

[3] Bhatt S, Gething PW, Brady OJ, Messina JP, Farlow AW, Moyes CL, et al. The global distribution and burden of dengue. Nature. 2013;496(7446):504–7. doi: 10.1038/nature12060 23563266 PMC3651993

[4] Gubler DJ. Dengue and dengue hemorrhagic fever. Clin Microbiol Rev. 1998;11(3):480–96. doi: 10.1128/CMR.11.3.480 9665979 PMC88892

[5] Caldwell M, Boruah AP, Thakur KT. Acute neurologic emerging flaviviruses. Ther Adv Infect Dis. 2022;9. doi: 10.1177/20499361221102664 35719177 PMC9198421

[6] Moore SM. The current burden of Japanese encephalitis and the estimated impacts of vaccination: Combining estimates of the spatial distribution and transmission intensity of a zoonotic pathogen. PLoS Negl Trop Dis. 2021;15(10):e0009385. doi: 10.1371/journal.pntd.0009385 34644296 PMC8544850

[7] Wikan N, Smith DR. Zika virus: history of a newly emerging arbovirus. Lancet Infect Dis. 2016;16(7):e119–26. doi: 10.1016/S1473-3099(16)30010-X 27282424

[8] Guerrero Saldivia SE, Unnikrishnan S, Chavarria YY, Akindele AO, Jalkh AP, Eastmond AK, et al. Zika Virus: A Systematic Review of Teratogenesis, Congenital Anomalies, and Child Mortality. Cureus. 2023;15(2):e34735. doi: 10.7759/cureus.34735 36909038 PMC9998135

[9] Simmonds P, Becher P, Bukh J, Gould EA, Meyers G, Monath T, et al. ICTV Virus Taxonomy Profile: Flaviviridae. J Gen Virol. 2017;98(1):2–3. doi: 10.1099/jgv.0.000672 28218572 PMC5370391

[10] Noble CG, Shi P-Y. Structural biology of dengue virus enzymes: towards rational design of therapeutics. Antiviral Res. 2012;96(2):115–26. doi: 10.1016/j.antiviral.2012.09.007 22995600

[11] Acosta EG, Kumar A, Bartenschlager R. Revisiting dengue virus-host cell interaction: new insights into molecular and cellular virology. Adv Virus Res. 2014;88:1–109. doi: 10.1016/B978-0-12-800098-4.00001-5 24373310

[12] Aguirre S, Maestre AM, Pagni S, Patel JR, Savage T, Gutman D, et al. DENV inhibits type I IFN production in infected cells by cleaving human STING. PLoS Pathog. 2012;8(10):e1002934. doi: 10.1371/journal.ppat.1002934 23055924 PMC3464218

[13] Angleró-Rodríguez YI, Pantoja P, Sariol CA. Dengue virus subverts the interferon induction pathway via NS2B/3 protease-IκB kinase epsilon interaction. Clin Vaccine Immunol. 2014;21(1):29–38. doi: 10.1128/CVI.00500-13 24173023 PMC3910921

[14] Chan YK, Gack MU. A phosphomimetic-based mechanism of dengue virus to antagonize innate immunity. Nat Immunol. 2016;17(5):523–30. doi: 10.1038/ni.3393 26998762 PMC4837045

[15] Luo D, Xu T, Hunke C, Grüber G, Vasudevan SG, Lescar J. Crystal structure of the NS3 protease-helicase from dengue virus. J Virol. 2008;82(1):173–83. doi: 10.1128/JVI.01788-07 17942558 PMC2224403

[16] Falgout B, Pethel M, Zhang YM, Lai CJ. Both nonstructural proteins NS2B and NS3 are required for the proteolytic processing of dengue virus nonstructural proteins. J Virol. 1991;65(5):2467–75. doi: 10.1128/JVI.65.5.2467-2475.1991 2016768 PMC240601

[17] Lim SP, Noble CG, Shi P-Y. The dengue virus NS5 protein as a target for drug discovery. Antiviral Res. 2015;119:57–67. doi: 10.1016/j.antiviral.2015.04.010 25912817

[18] El Sahili A, Lescar J. Dengue Virus Non-Structural Protein 5. Viruses. 2017;9(4):91. doi: 10.3390/v9040091 28441781 PMC5408697

[19] Tay MYF, Saw WG, Zhao Y, Chan KWK, Singh D, Chong Y, et al. The C-terminal 50 amino acid residues of dengue NS3 protein are important for NS3-NS5 interaction and viral replication. J Biol Chem. 2015;290(4):2379–94. doi: 10.1074/jbc.M114.607341 25488659 PMC4303688

[20] Zou G, Chen Y-L, Dong H, Lim CC, Yap LJ, Yau YH, et al. Functional analysis of two cavities in flavivirus NS5 polymerase. J Biol Chem. 2011;286(16):14362–72. doi: 10.1074/jbc.M110.214189 21349834 PMC3077636

[21] Lescar J, Soh S, Lee LT, Vasudevan SG, Kang C, Lim SP. The Dengue Virus Replication Complex: From RNA Replication to Protein-Protein Interactions to Evasion of Innate Immunity. Adv Exp Med Biol. 2018;1062:115–29. doi: 10.1007/978-981-10-8727-1_9 29845529

[22] Wakil SJ. Fatty acid synthase, a proficient multifunctional enzyme. Biochemistry. 1989;28(11):4523–30. doi: 10.1021/bi00437a001 2669958

[23] Tongluan N, Ramphan S, Wintachai P, Jaresitthikunchai J, Khongwichit S, Wikan N, et al. Involvement of fatty acid synthase in dengue virus infection. Virol J. 2017;14(1):28. doi: 10.1186/s12985-017-0685-9 28193229 PMC5307738

[24] Hitakarun A, Khongwichit S, Wikan N, Roytrakul S, Yoksan S, Rajakam S, et al. Evaluation of the antiviral activity of orlistat (tetrahydrolipstatin) against dengue virus, Japanese encephalitis virus, Zika virus and chikungunya virus. Sci Rep. 2020;10(1):1499. doi: 10.1038/s41598-020-58468-8 32001767 PMC6992670

[25] Heaton NS, Perera R, Berger KL, Khadka S, Lacount DJ, Kuhn RJ, et al. Dengue virus nonstructural protein 3 redistributes fatty acid synthase to sites of viral replication and increases cellular fatty acid synthesis. Proc Natl Acad Sci U S A. 2010;107(40):17345–50. doi: 10.1073/pnas.1010811107 20855599 PMC2951450

[26] Tang W-C, Lin R-J, Liao C-L, Lin Y-L. Rab18 facilitates dengue virus infection by targeting fatty acid synthase to sites of viral replication. J Virol. 2014;88(12):6793–804. doi: 10.1128/JVI.00045-14 24696471 PMC4054357

[27] Kovanich D, Saisawang C, Sittipaisankul P, Ramphan S, Kalpongnukul N, Somparn P, et al. Analysis of the Zika and Japanese Encephalitis Virus NS5 Interactomes. J Proteome Res. 2019;18(8):3203–18. doi: 10.1021/acs.jproteome.9b00318 31199156

[28] Schneider CA, Rasband WS, Eliceiri KW. NIH Image to ImageJ: 25 years of image analysis. Nat Methods. 2012;9(7):671–5. doi: 10.1038/nmeth.2089 22930834 PMC5554542

[29] French AP, Mills S, Swarup R, Bennett MJ, Pridmore TP. Colocalization of fluorescent markers in confocal microscope images of plant cells. Nat Protoc. 2008;3(4):619–28. doi: 10.1038/nprot.2008.31 18388944

[30] Srisutthisamphan K, Jirakanwisal K, Ramphan S, Tongluan N, Kuadkitkan A, Smith DR. Hsp90 interacts with multiple dengue virus 2 proteins. Sci Rep. 2018;8(1):4308. doi: 10.1038/s41598-018-22639-5 29523827 PMC5844963

[31] Ye J, Chen Z, Zhang B, Miao H, Zohaib A, Xu Q, et al. Heat shock protein 70 is associated with replicase complex of Japanese encephalitis virus and positively regulates viral genome replication. PLoS One. 2013;8(9):e75188. doi: 10.1371/journal.pone.0075188 24086464 PMC3781048

[32] Edward Z, Takegami T. Localization and functions of Japanese encephalitis virus nonstructural proteins NS3 and NS5 for viral RNA synthesis in the infected cells. Microbiol Immunol. 1993;37(3):239–43. doi: 10.1111/j.1348-0421.1993.tb03206.x 8321152

[33] Dutta SK, Langenburg T. A Perspective on Current Flavivirus Vaccine Development: A Brief Review. Viruses. 2023;15(4):860. doi: 10.3390/v15040860 37112840 PMC10142581

[34] Halstead SB, Dans LF. Dengue infection and advances in dengue vaccines for children. Lancet Child Adolesc Health. 2019;3(10):734–41. doi: 10.1016/S2352-4642(19)30205-6 31378686

[35] Halstead SB, Katzelnick LC, Russell PK, Markoff L, Aguiar M, Dans LR, et al. Ethics of a partially effective dengue vaccine: Lessons from the Philippines. Vaccine. 2020;38(35):5572–6. doi: 10.1016/j.vaccine.2020.06.079 32654899 PMC7347470

[36] Flacco ME, Bianconi A, Cioni G, Fiore M, Calò GL, Imperiali G, et al. Immunogenicity, Safety and Efficacy of the Dengue Vaccine TAK-003: A Meta-Analysis. Vaccines (Basel). 2024;12(7):770. doi: 10.3390/vaccines12070770 39066408 PMC11281463

[37] Smith DR. Waiting in the wings: The potential of mosquito transmitted flaviviruses to emerge. Crit Rev Microbiol. 2017;43(4):405–22. doi: 10.1080/1040841X.2016.1230974 27800692

[38] Singh N, Vayer P, Tanwar S, Poyet J-L, Tsaioun K, Villoutreix BO. Drug discovery and development: introduction to the general public and patient groups. Front Drug Discov. 2023;3. doi: 10.3389/fddsv.2023.1201419

[39] Leier HC, Messer WB, Tafesse FG. Lipids and pathogenic flaviviruses: An intimate union. PLoS Pathog. 2018;14(5):e1006952. doi: 10.1371/journal.ppat.1006952 29746606 PMC5944919

[40] Martín-Acebes MA, Merino-Ramos T, Blázquez A-B, Casas J, Escribano-Romero E, Sobrino F, et al. The composition of West Nile virus lipid envelope unveils a role of sphingolipid metabolism in flavivirus biogenesis. J Virol. 2014;88(20):12041–54. doi: 10.1128/JVI.02061-14 25122799 PMC4178726

[41] Cui L, Lee YH, Kumar Y, Xu F, Lu K, Ooi EE, et al. Serum metabolome and lipidome changes in adult patients with primary dengue infection. PLoS Negl Trop Dis. 2013;7(8):e2373. doi: 10.1371/journal.pntd.0002373 23967362 PMC3744433

[42] Durán A, Carrero R, Parra B, González A, Delgado L, Mosquera J, et al. Association of lipid profile alterations with severe forms of dengue in humans. Arch Virol. 2015;160(7):1687–92. doi: 10.1007/s00705-015-2433-z 25936955

[43] Parzych KR, Klionsky DJ. An overview of autophagy: morphology, mechanism, and regulation. Antioxid Redox Signal. 2014;20(3):460–73. doi: 10.1089/ars.2013.5371 23725295 PMC3894687

[44] Lee Y-R, Lei H-Y, Liu M-T, Wang J-R, Chen S-H, Jiang-Shieh Y-F, et al. Autophagic machinery activated by dengue virus enhances virus replication. Virology. 2008;374(2):240–8. doi: 10.1016/j.virol.2008.02.016 18353420 PMC7103294

[45] Panyasrivanit M, Khakpoor A, Wikan N, Smith DR. Co-localization of constituents of the dengue virus translation and replication machinery with amphisomes. J Gen Virol. 2009;90(Pt 2):448–56. doi: 10.1099/vir.0.005355-0 19141455

[46] Heaton NS, Randall G. Dengue virus-induced autophagy regulates lipid metabolism. Cell Host Microbe. 2010;8(5):422–32. doi: 10.1016/j.chom.2010.10.006 21075353 PMC3026642

[47] Jones SF, Infante JR. Molecular Pathways: Fatty Acid Synthase. Clin Cancer Res. 2015;21(24):5434–8. doi: 10.1158/1078-0432.CCR-15-0126 26519059

[48] Kapoor M, Zhang L, Mohan PM, Padmanabhan R. Synthesis and characterization of an infectious dengue virus type-2 RNA genome (New Guinea C strain). Gene. 1995;162(2):175–80. doi: 10.1016/0378-1119(95)00332-z 7557426

[49] Kumar A, Bühler S, Selisko B, Davidson A, Mulder K, Canard B, et al. Nuclear localization of dengue virus nonstructural protein 5 does not strictly correlate with efficient viral RNA replication and inhibition of type I interferon signaling. J Virol. 2013;87(8):4545–57. doi: 10.1128/JVI.03083-12 23408610 PMC3624364

[50] Cheng CX, Tan MJA, Chan KWK, Choy MMJ, Roman N, Arnold DDR, et al. Serotype-Specific Regulation of Dengue Virus NS5 Protein Subcellular Localization. ACS Infect Dis. 2024;10(6):2047–62. doi: 10.1021/acsinfecdis.4c00054 38811007 PMC11184549

[51] Hannemann H, Sung P-Y, Chiu H-C, Yousuf A, Bird J, Lim SP, et al. Serotype-specific differences in dengue virus non-structural protein 5 nuclear localization. J Biol Chem. 2013;288(31):22621–35. doi: 10.1074/jbc.M113.481382 23770669 PMC3829348

[52] Weber F, Wagner V, Rasmussen SB, Hartmann R, Paludan SR. Double-stranded RNA is produced by positive-strand RNA viruses and DNA viruses but not in detectable amounts by negative-strand RNA viruses. J Virol. 2006;80(10):5059–64. doi: 10.1128/JVI.80.10.5059-5064.2006 16641297 PMC1472073

