# Supplementary material for: The interaction of Orthoflavivirus nonstructural proteins 3 and 5 with human fatty acid synthase
Source: PLoS One. 2025 Mar 25;20(3):e0319207. doi: 10.1371/journal.pone.0319207 (PMC11936160; doi:10.1371/journal.pone.0319207)

Supplemental materials

The interaction of flavivirus nonstructural proteins 3 and 5 with human fatty acid synthase

Suthatta Sornprasert[1], Wannapa Sornjai[1], Duncan R. Smith[1]*

[1]Institute of Molecular Biosciences, Mahidol University, Salaya, 73170, Thailand;

* Correspondence: duncan_r_smith@hotmail.com; Tel.: +66-2800-3624-8

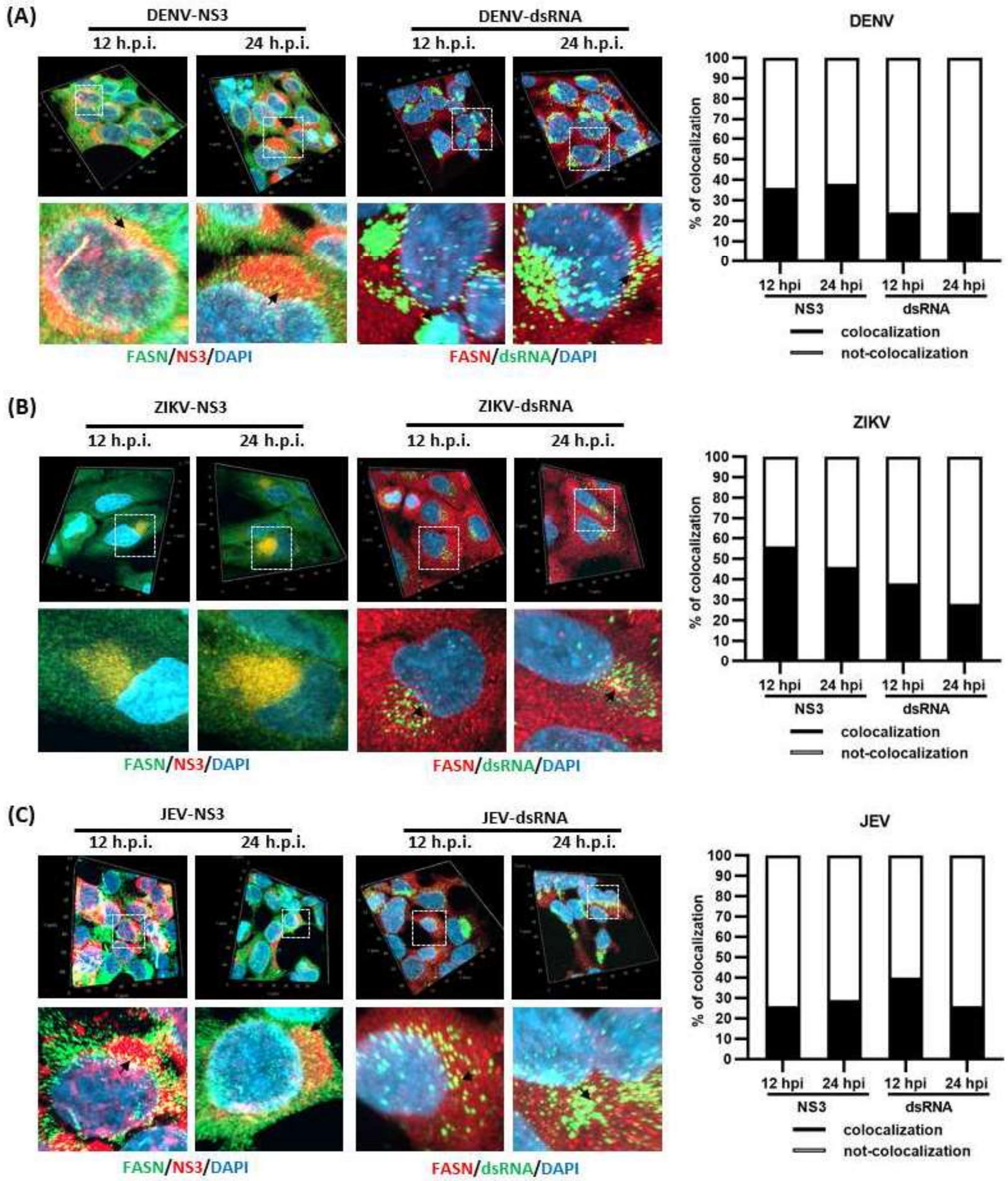

Figure S1. The three-dimensional analysis of A549 cells infected with DENV, JEV, and ZIKV at 12 and 24 hpi. Confocal microscopy determined the colocalization between FASN and NS3/dsRNA of (A) DENV (B) ZIKV (C) JEV. The images were taken in approximately 30 stacks, with 63X magnification and 1.5X Zoom. Each color represented the different fluorochrome staining proteins. For staining of FASN and NS3, FASN was represented in green (Alexa^{TM} Fluor 488), and NS3 was represented in red (Alexa^{TM} Fluor 647). For staining of FASN and dsRNA, FASN was represented in red (Alexa^{TM} Fluor 647), and NS3 was represented in green (Alexa^{TM} Fluor 488). The nucleus was represented in blue (DAPI). The percent colocalization was analyzed by Imaris program (version 9.9.0) shown in the right panel.

Table S1. Primer sequences for construct NS3 and FASN.

Gene names	Primer names	Sequences
DENV 2 - NS3	DEN 2 NS3-eGFPC2-F	gcgaagcttagccggagtattgtgggatgttc
	DEN 2 NS3-eGFPC2-R	aaaggtaccctactttcttccggctgcaaattcc
ZIKV - NS3	ZIKV NS3-eGFPC2-F	attggaattcagtggtgctctatgggatgtgc
	ZIKV NS3-eGFPC2-R	aaaggtaccctatcttttcccagcggcaaactcc
JEV - NS3	JEV NS3-eGFPC2-F	attggaattcgggggcgtgttttgggacacg
	JEV NS3-eGFPC2-R	aaaggtaccctatctcttccctgctgcaaagtctttg
Human FASN	C2-FASN-Hindlll-F	gcgaagcttagaggaggtggtgattgccgg
	FASN-NheI-R	ttccaggctagcctccaggatgc
	FASN-NheI-F	atcctggaggctagcctggaaggtg
	C2-FASN-Kpnl-R	aaaggtaccctagccctcccgcacgc

Figure 1A ; ZIKV_DENV_JEV-FASN (Input)

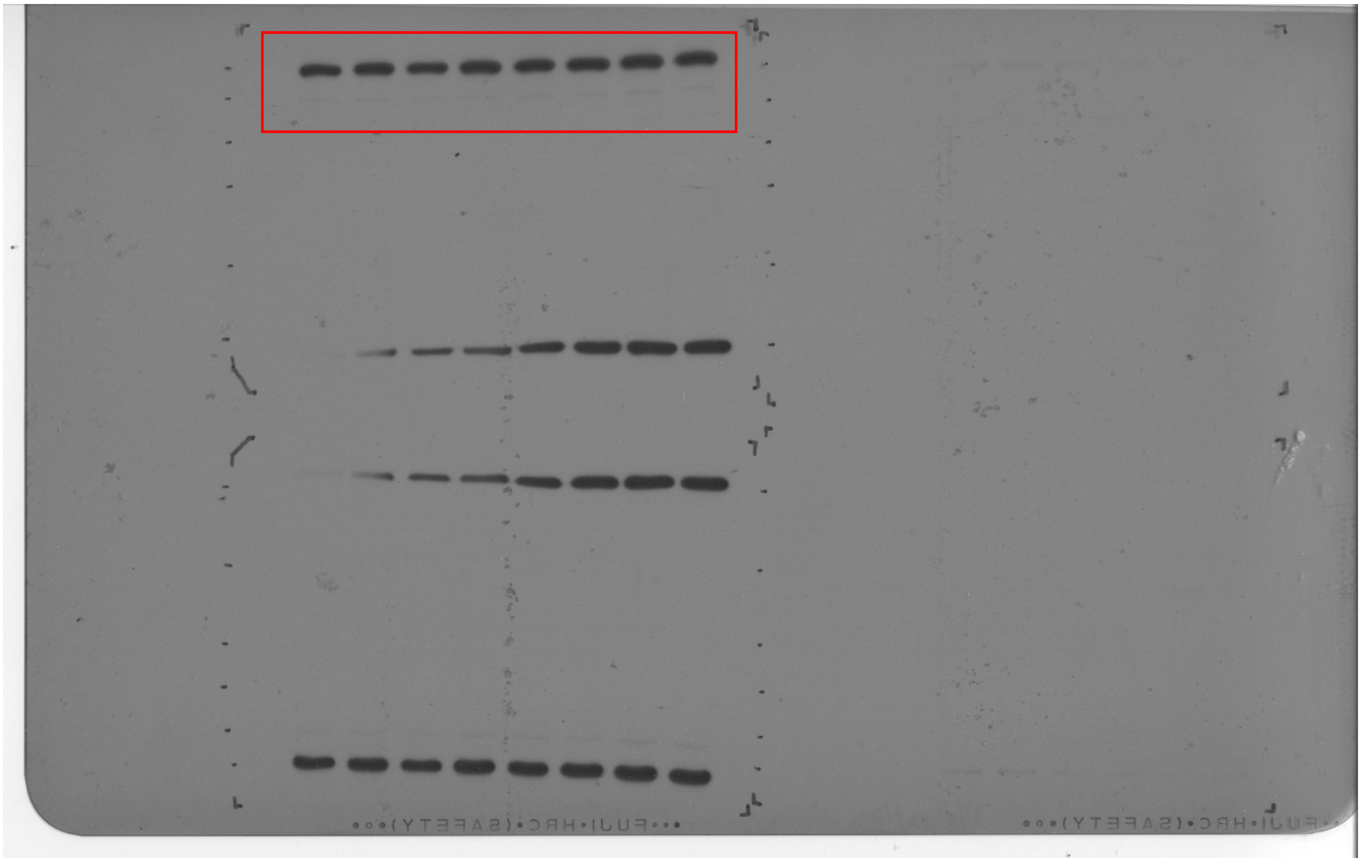

Figure 1A ; ZIKV_DENV_JEV-FASN (IP)

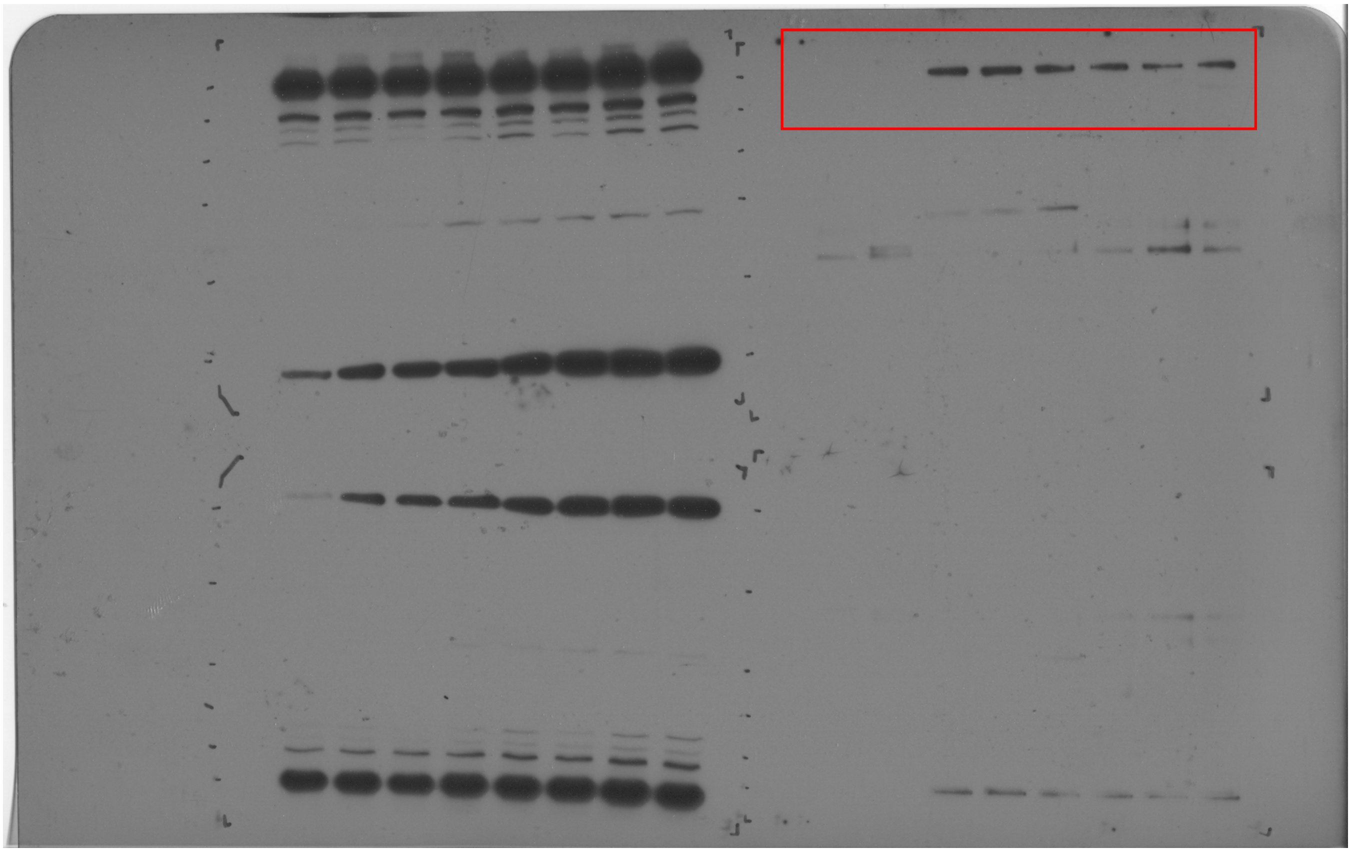

Figure 1A ; ZIKV_DENV_JEV-HSP90 (Input)

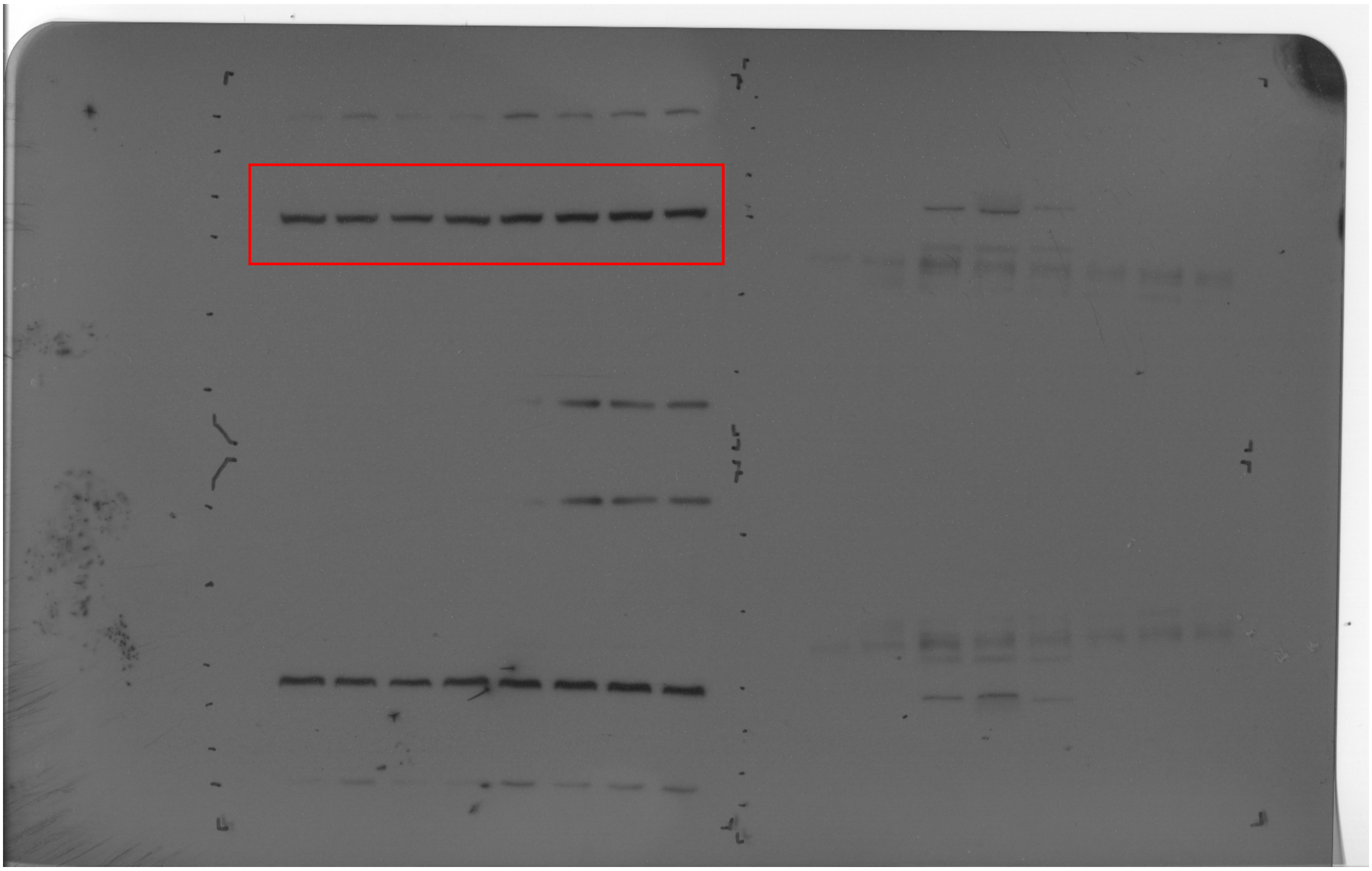

Figure 1A ; ZIKV_DENV_JEV-HSP90 (IP)

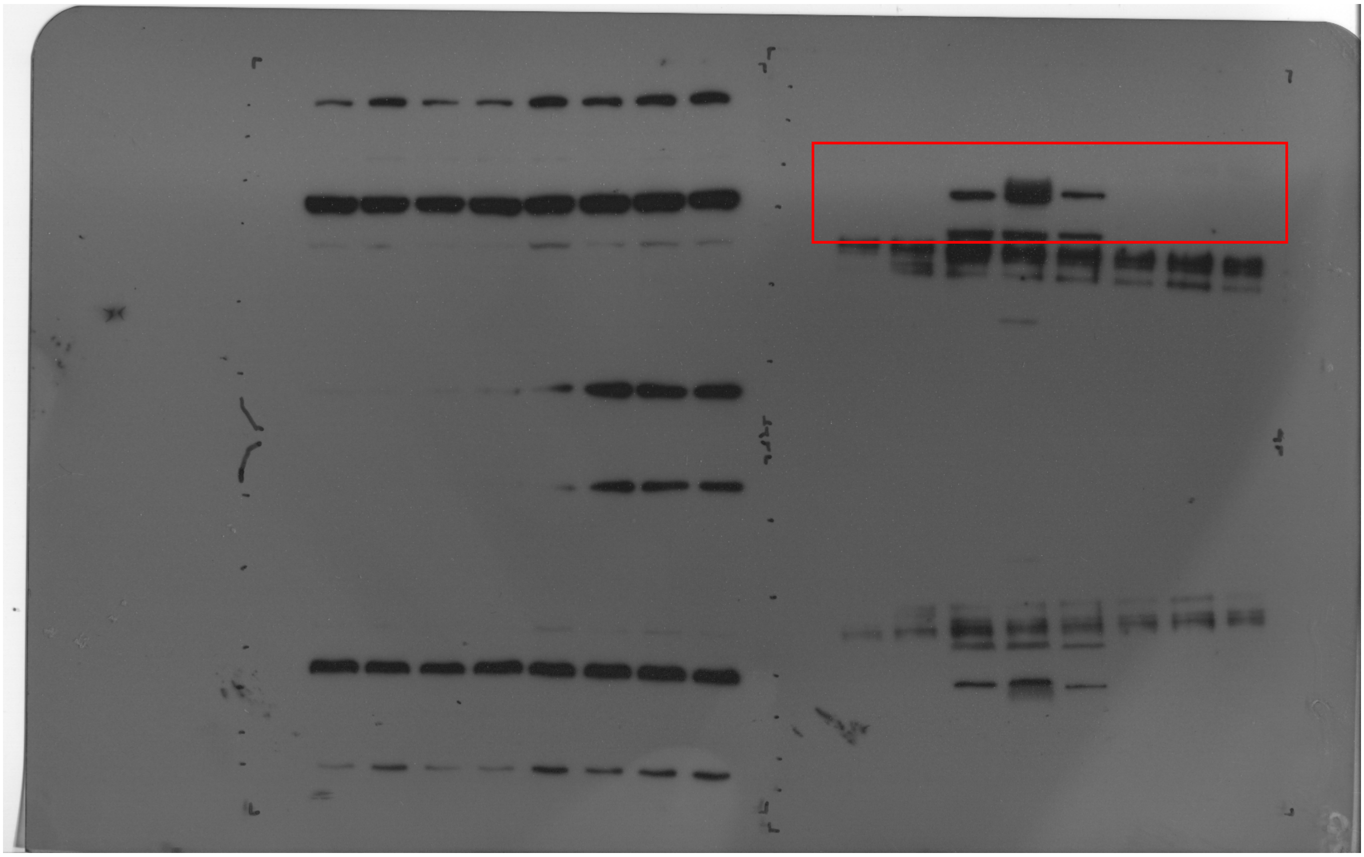

Figure 1A ; ZIKV_DENV_JEV-GFP (Input)

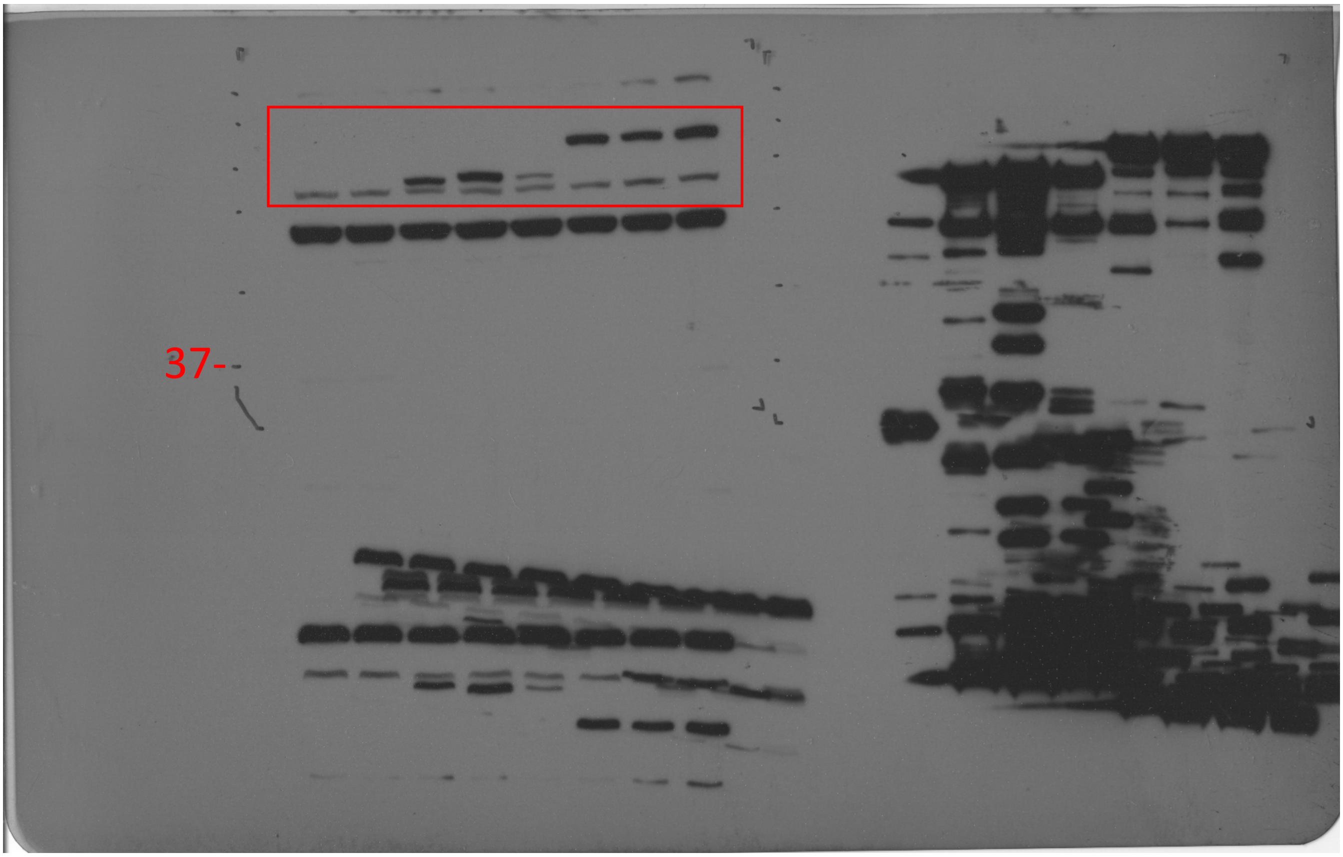

Figure 1A ; ZIKV_DENV_JEV-GFP (IP)

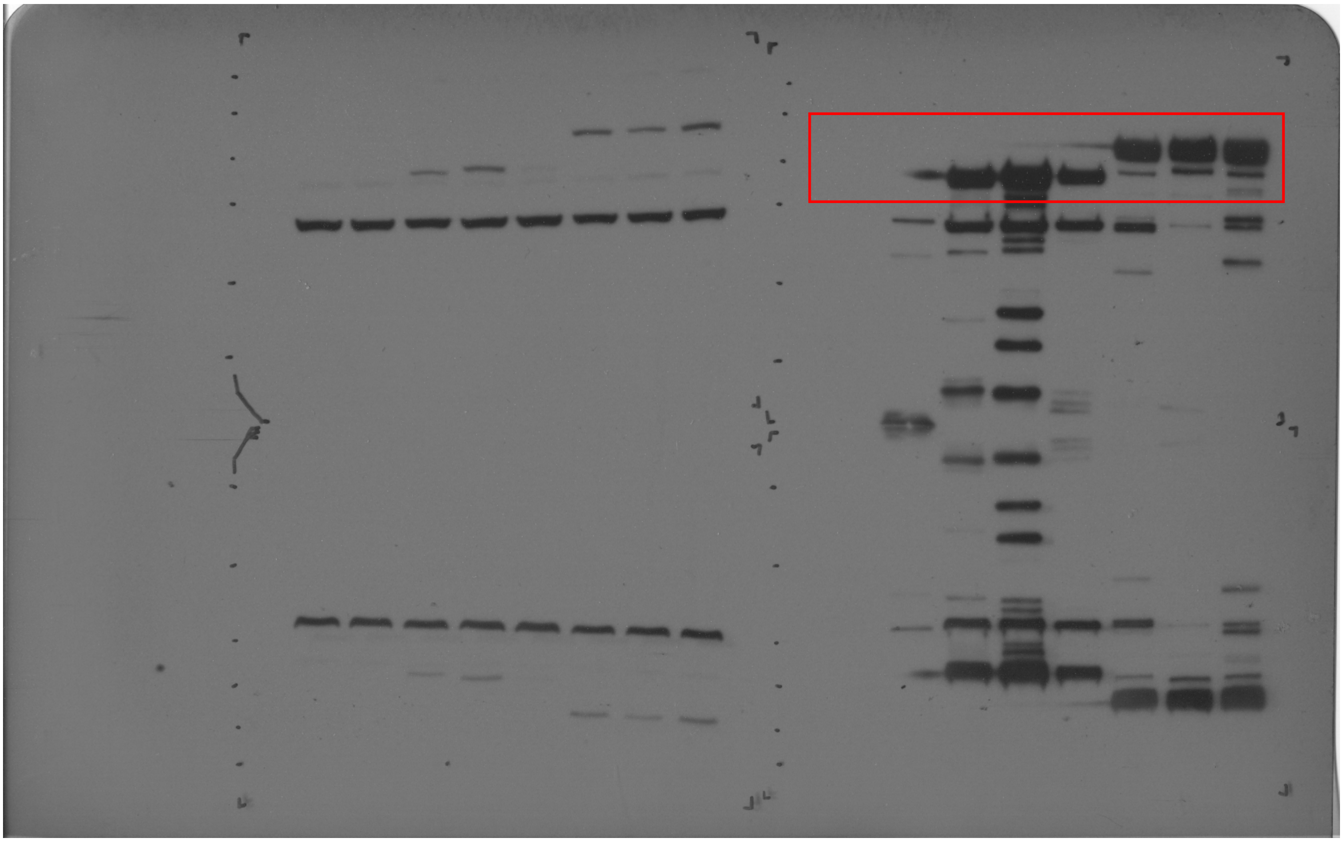

Figure 1A ; ZIKV_DENV_JEV-GFP (Input)

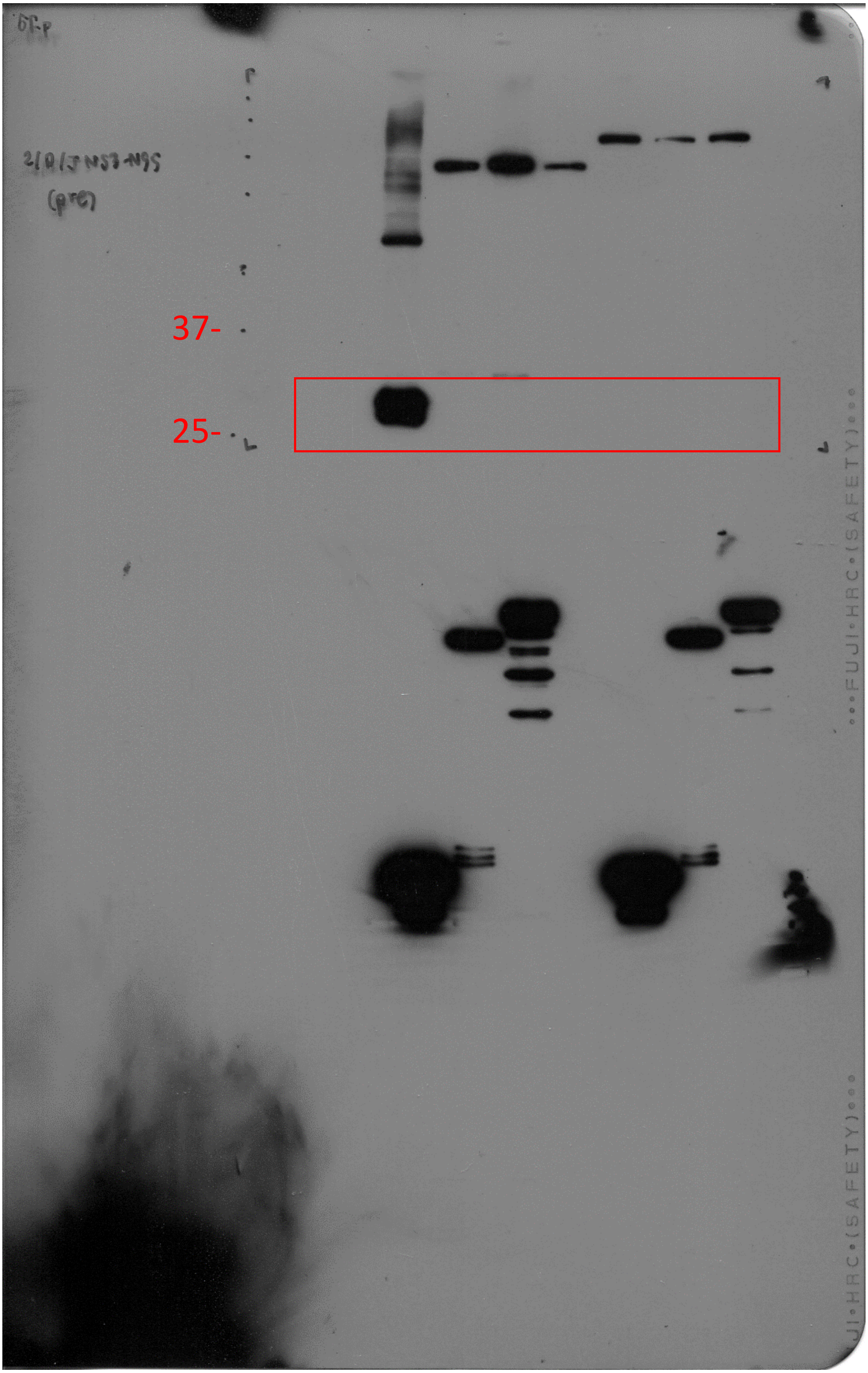

Figure 1A ; ZIKV_DENV_JEV-GFP (IP)

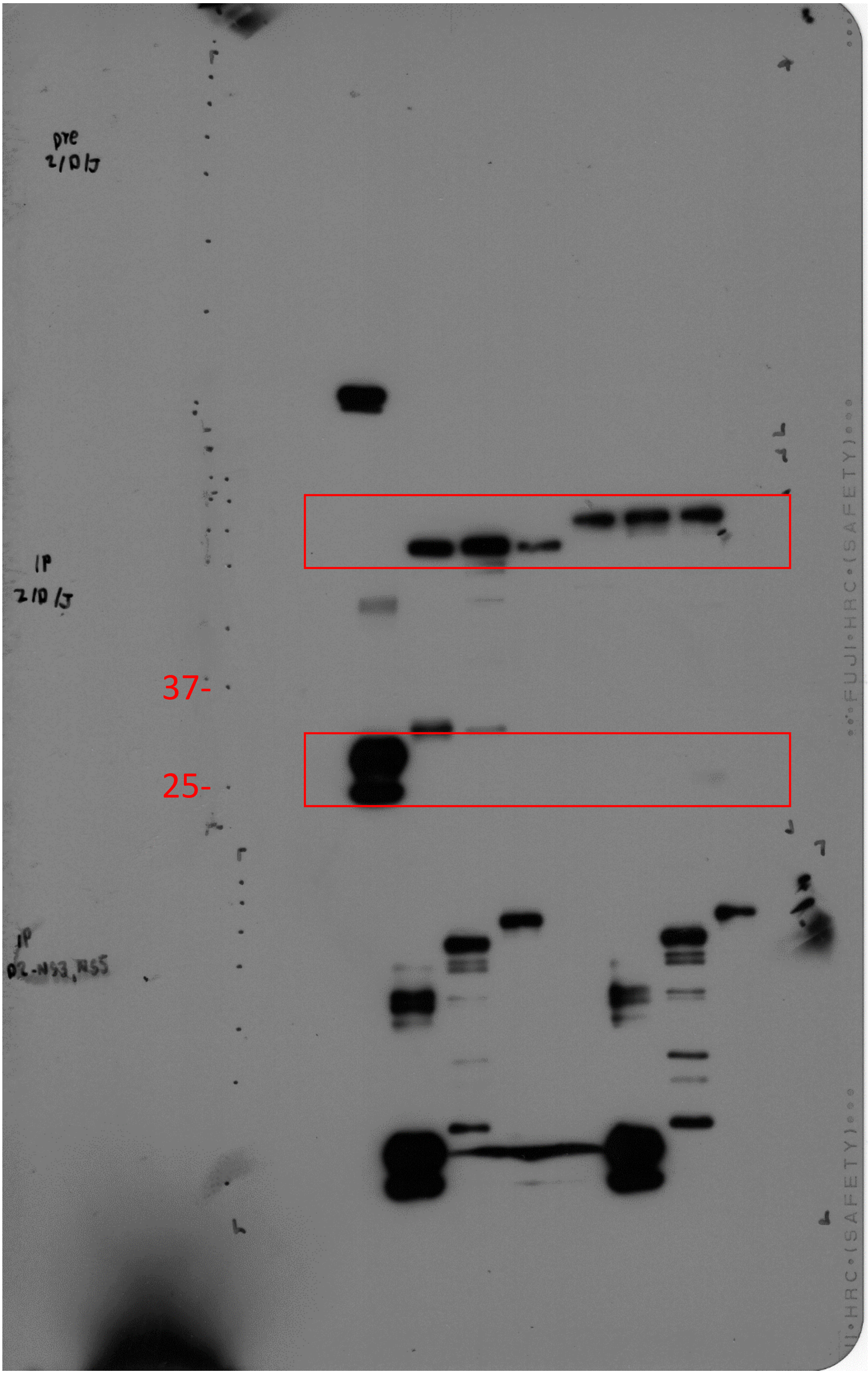

Figure 1A ; ZIKV_DENV_JEV-GRP78 (Input)

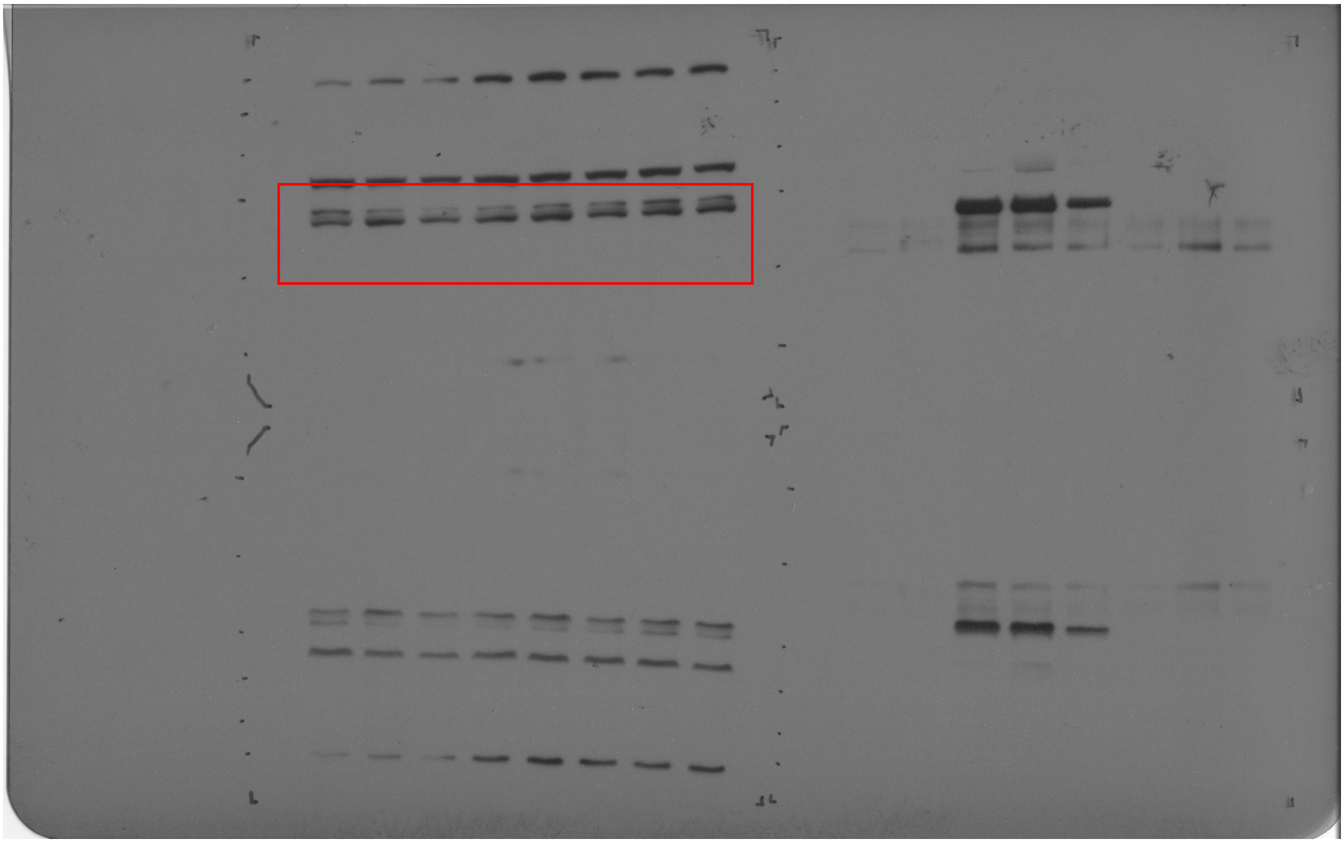

Figure 1A ; ZIKV_DENV_JEV-GRP78 (IP)

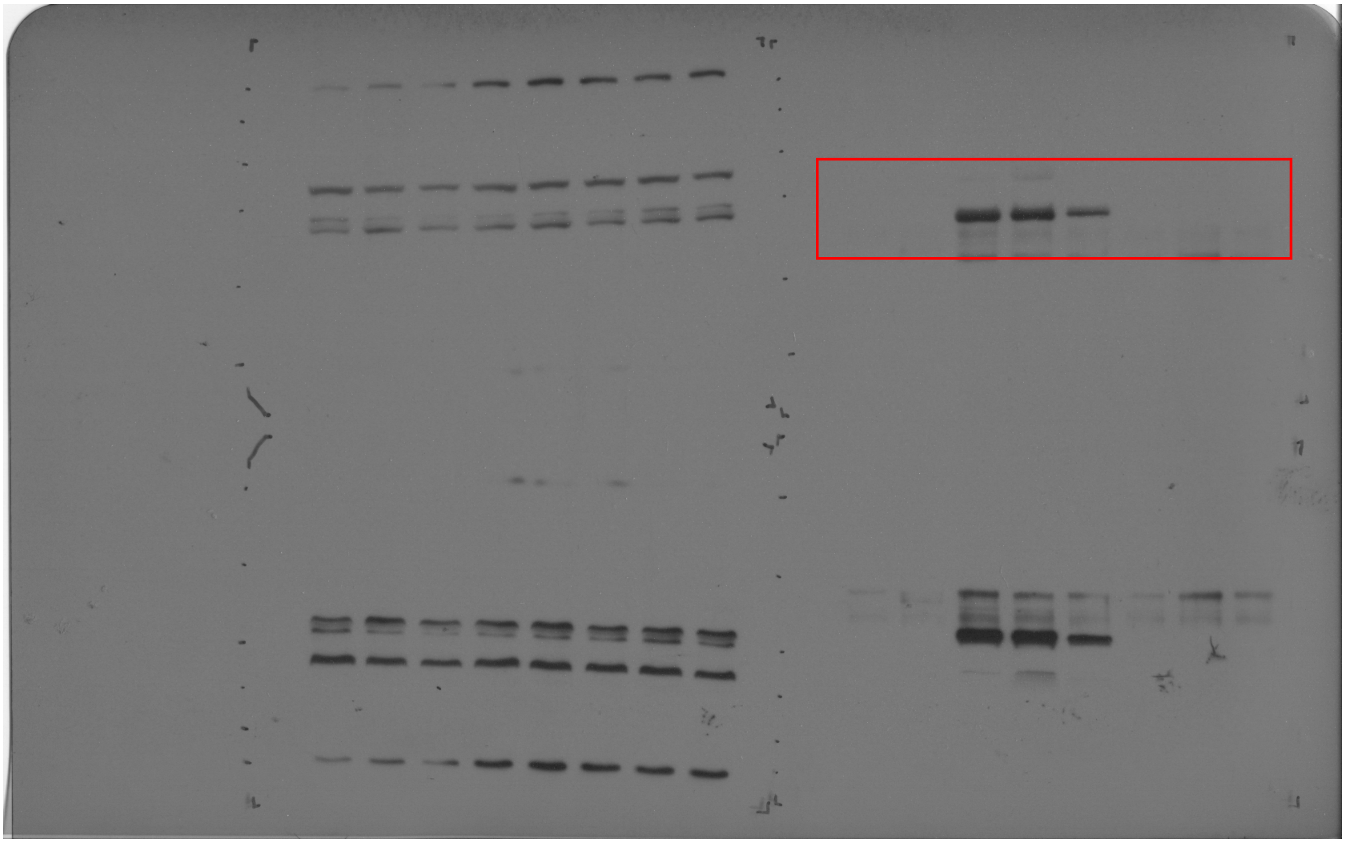

Figure 1A ; ZIKV_DENV_JEV-GAPDH (Input)

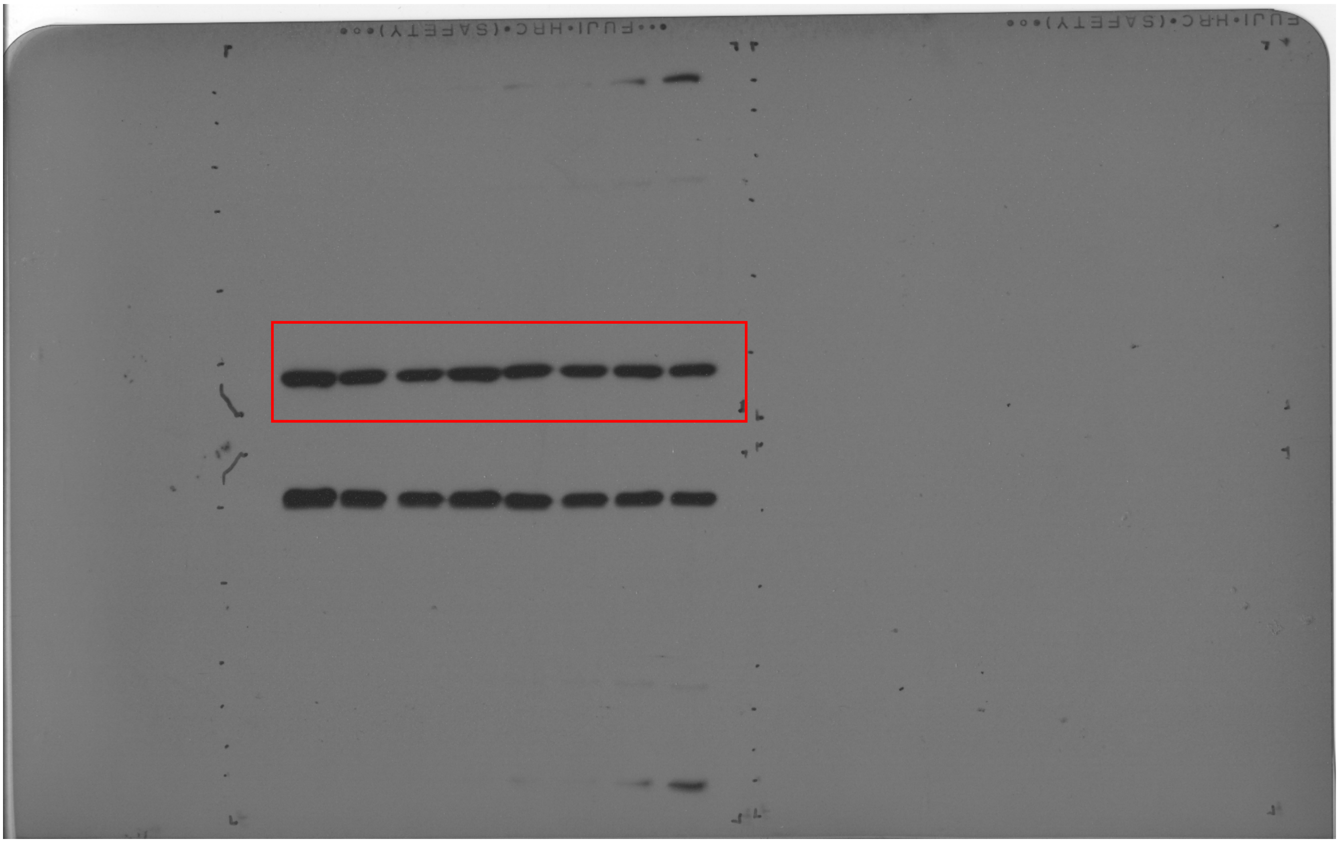

Figure 1A ; ZIKV_DENV_JEV-GAPDH (IP)

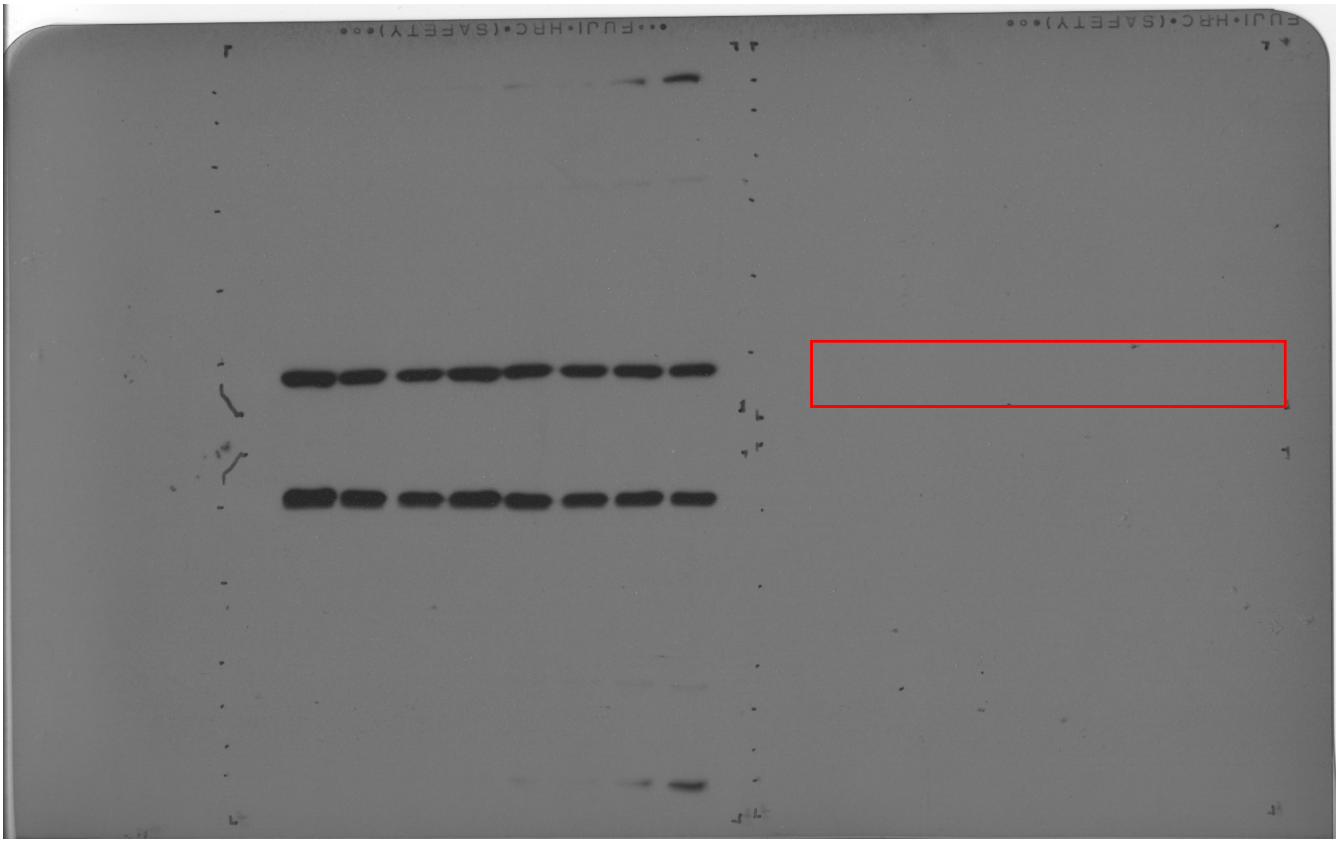

Figure 1A ; ZIKV_DENV_JEV-FASN (Input) #Replicate 2

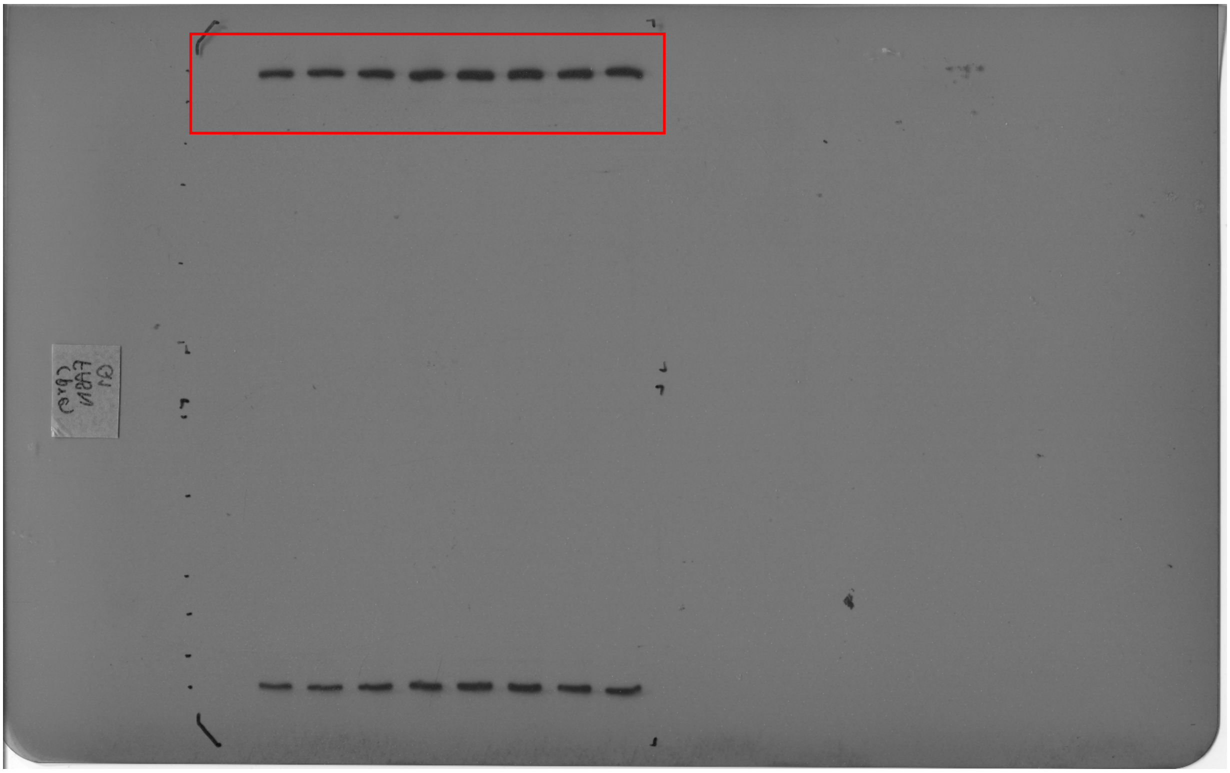

Figure 1A ; ZIKV_DENV_JEV-FASN (IP) #Replicate 2

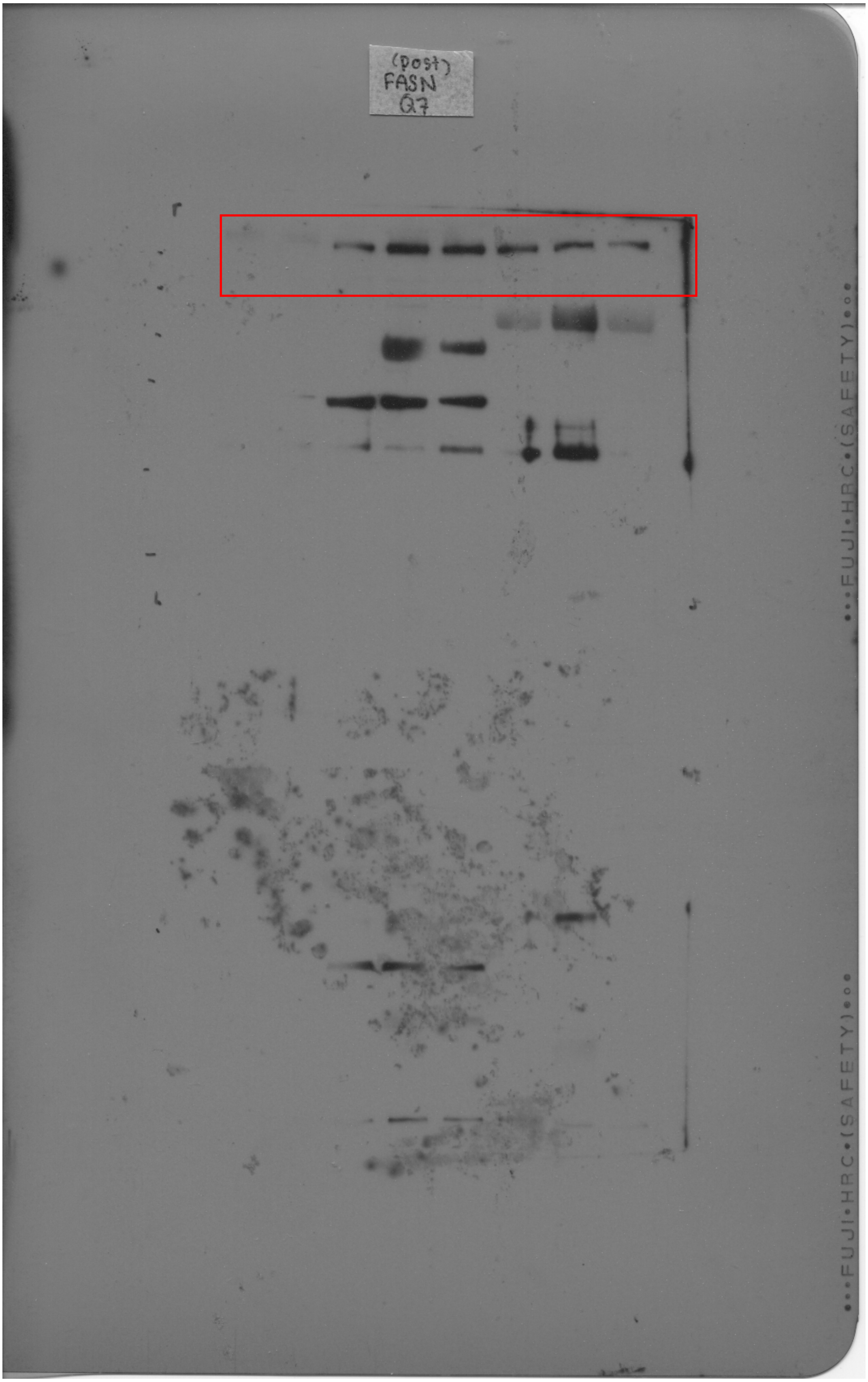

Figure 1A ; ZIKV_DENV_JEV-HSP90 (Input) #Replicate 2

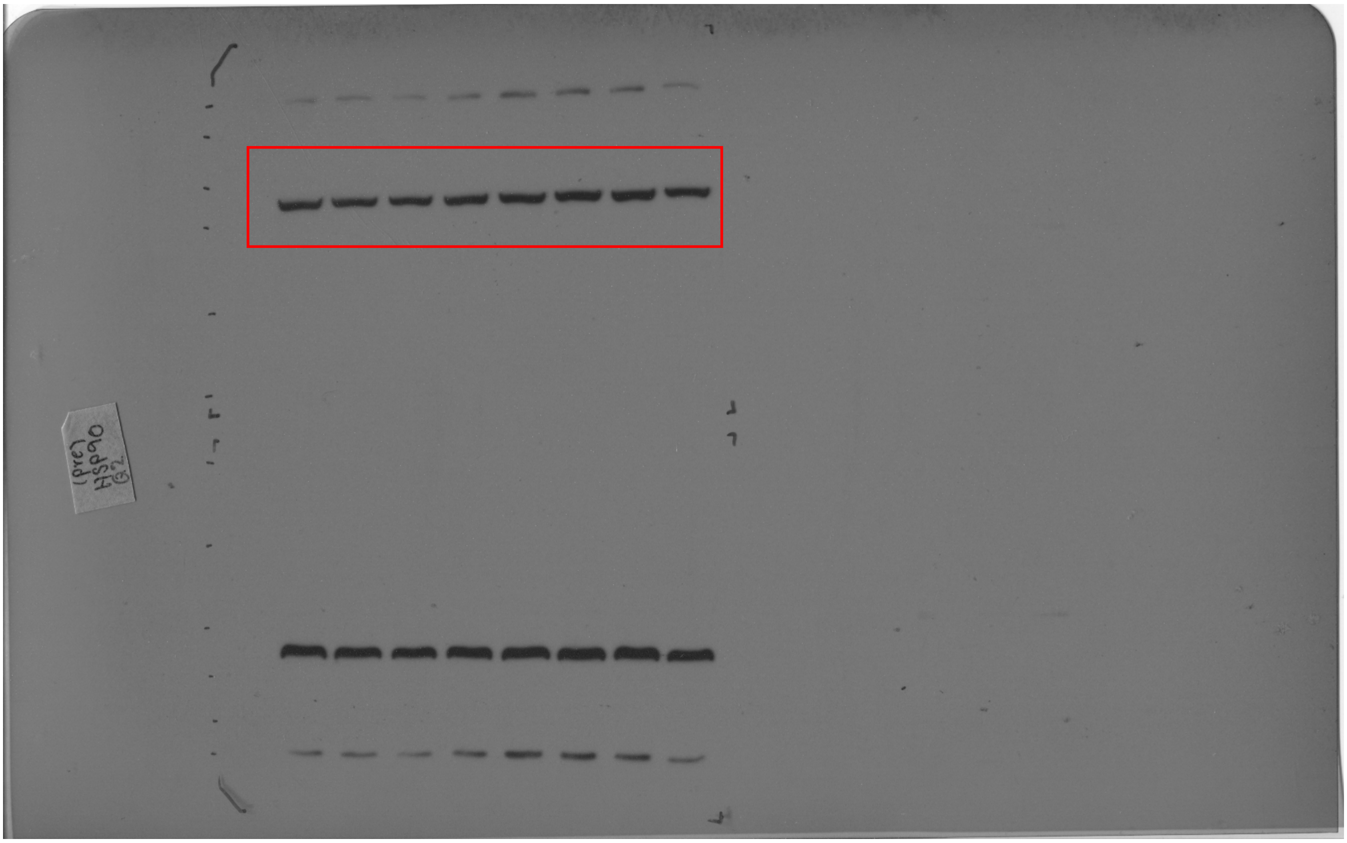

Figure 1A ; ZIKV_DENV_JEV-HSP90 (IP) #Replicate 2

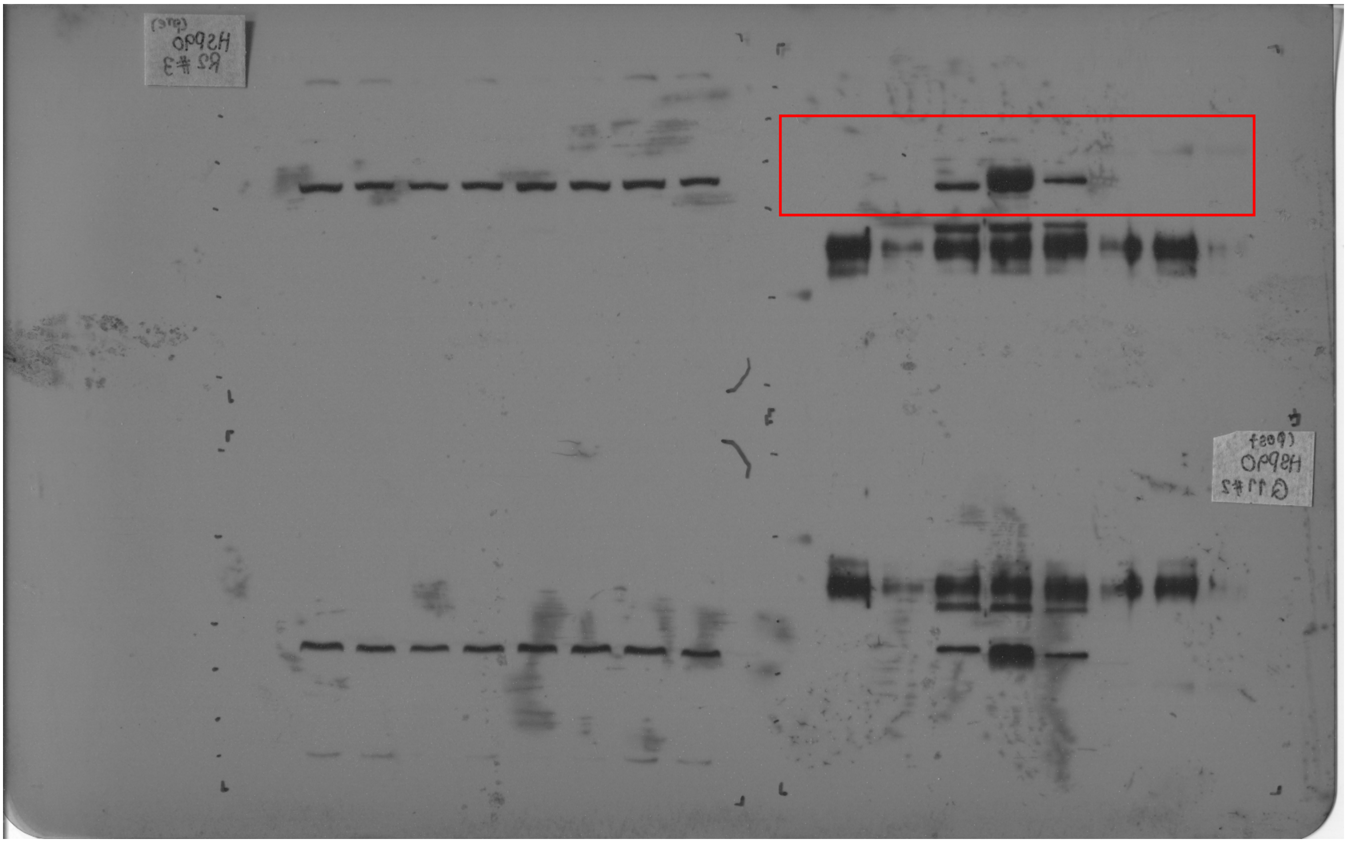

Figure 1A ; ZIKV_DENV_JEV-GFP (Input) #Replicate 2

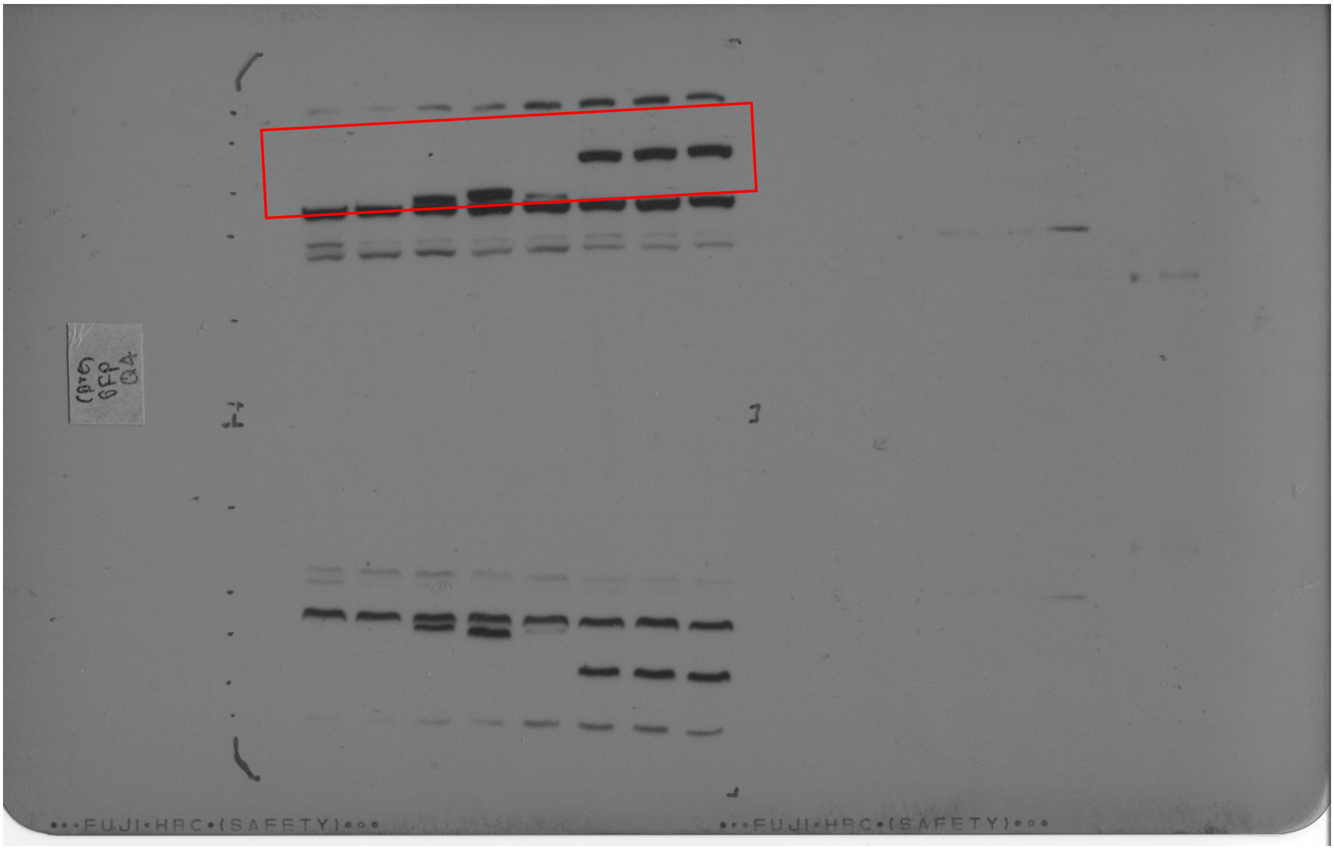

Figure 1A ; ZIKV_DENV_JEV-GFP (IP) #Replicate 2

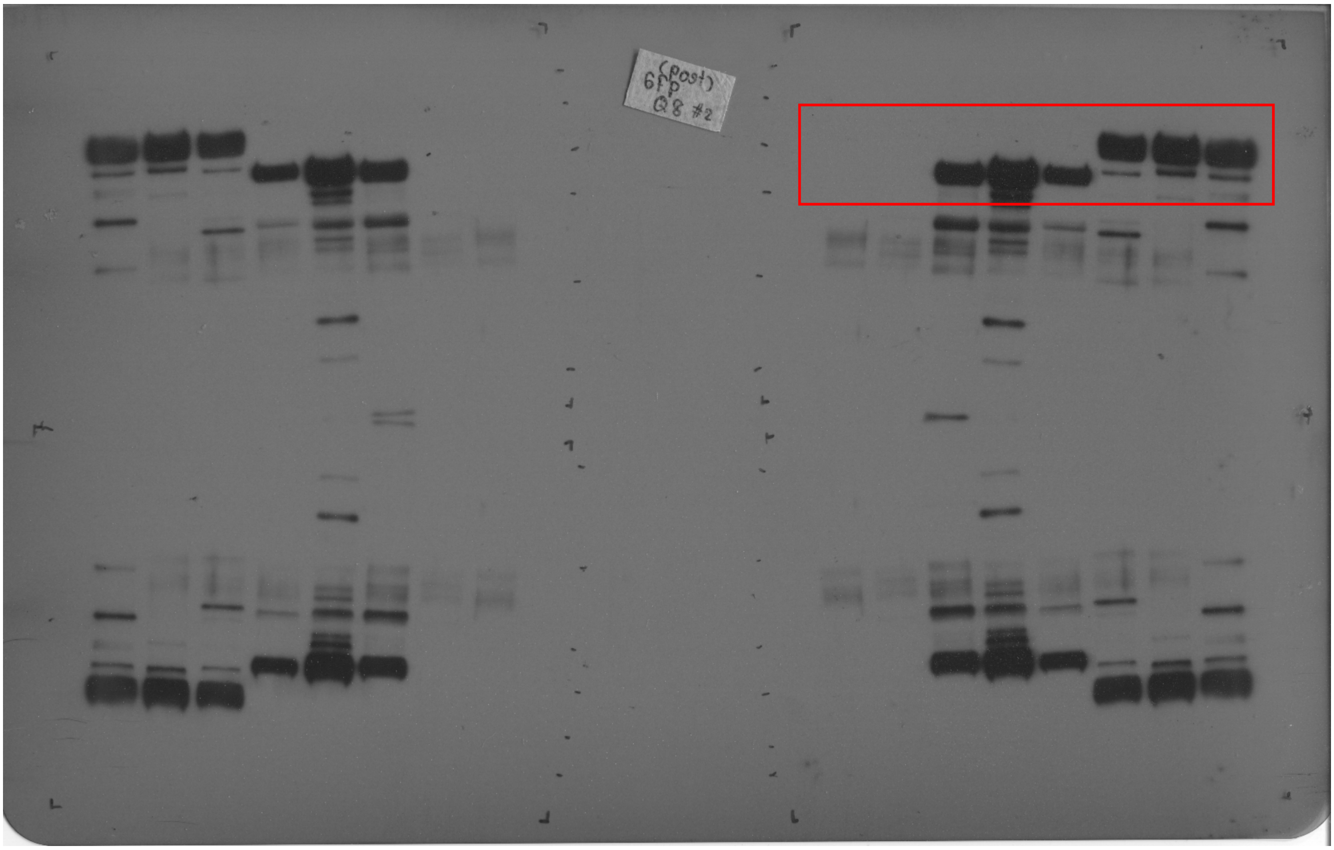

Figure 1A ; ZIKV_DENV_JEV-GRP78 (Input) #Replicate 2

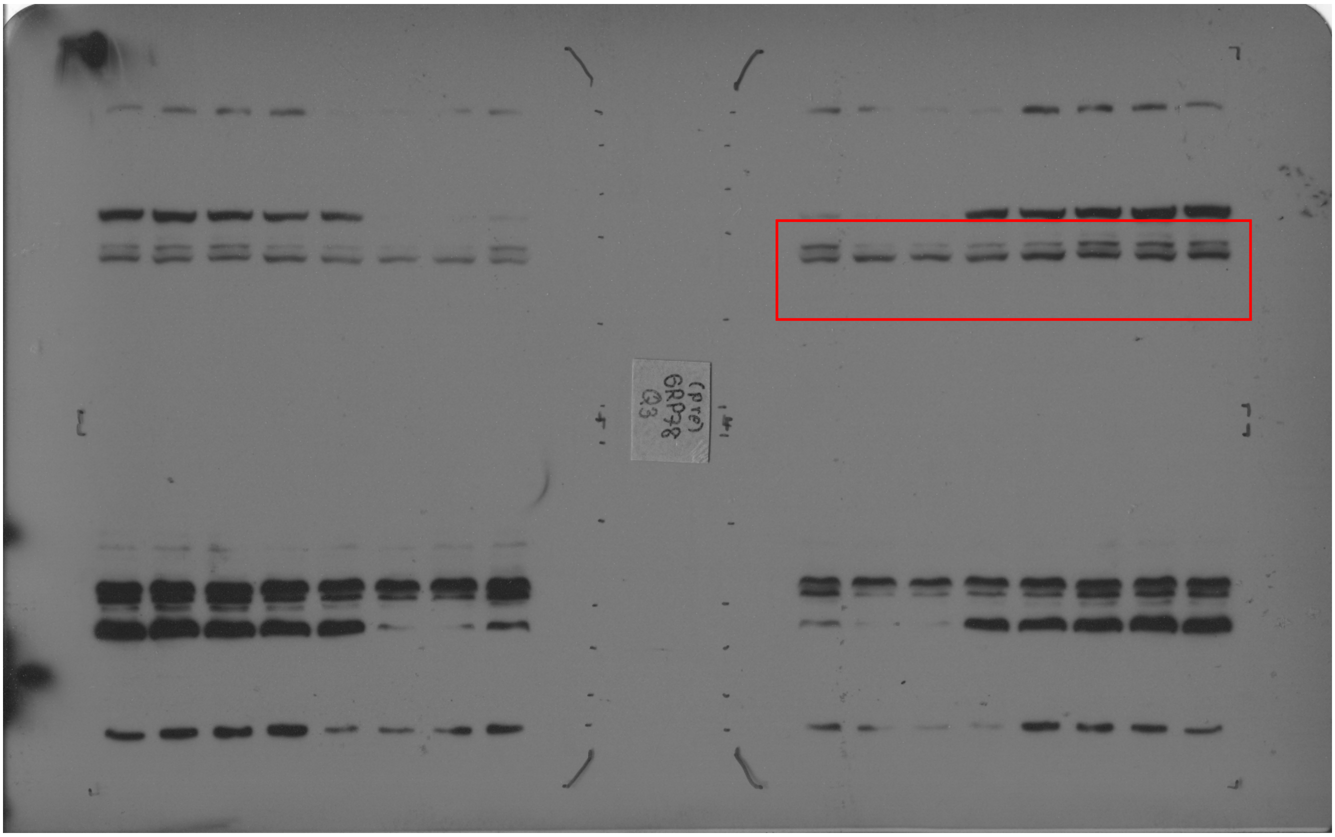

Figure 1A ; ZIKV_DENV_JEV-GRP78 (IP) #Replicate 2

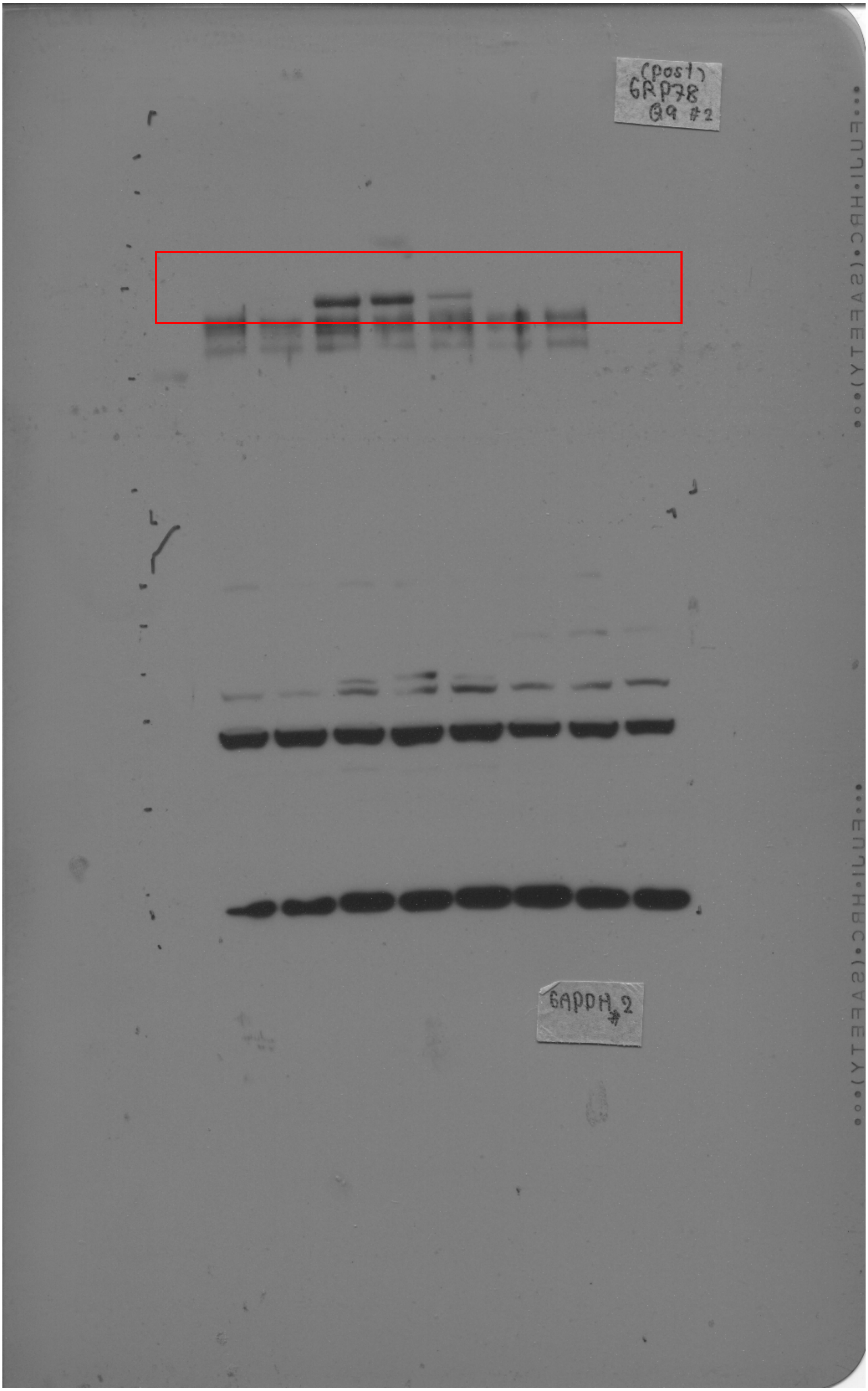

Figure 1A ; ZIKV_DENV_JEV-GAPDH (Input) #Replicate 2

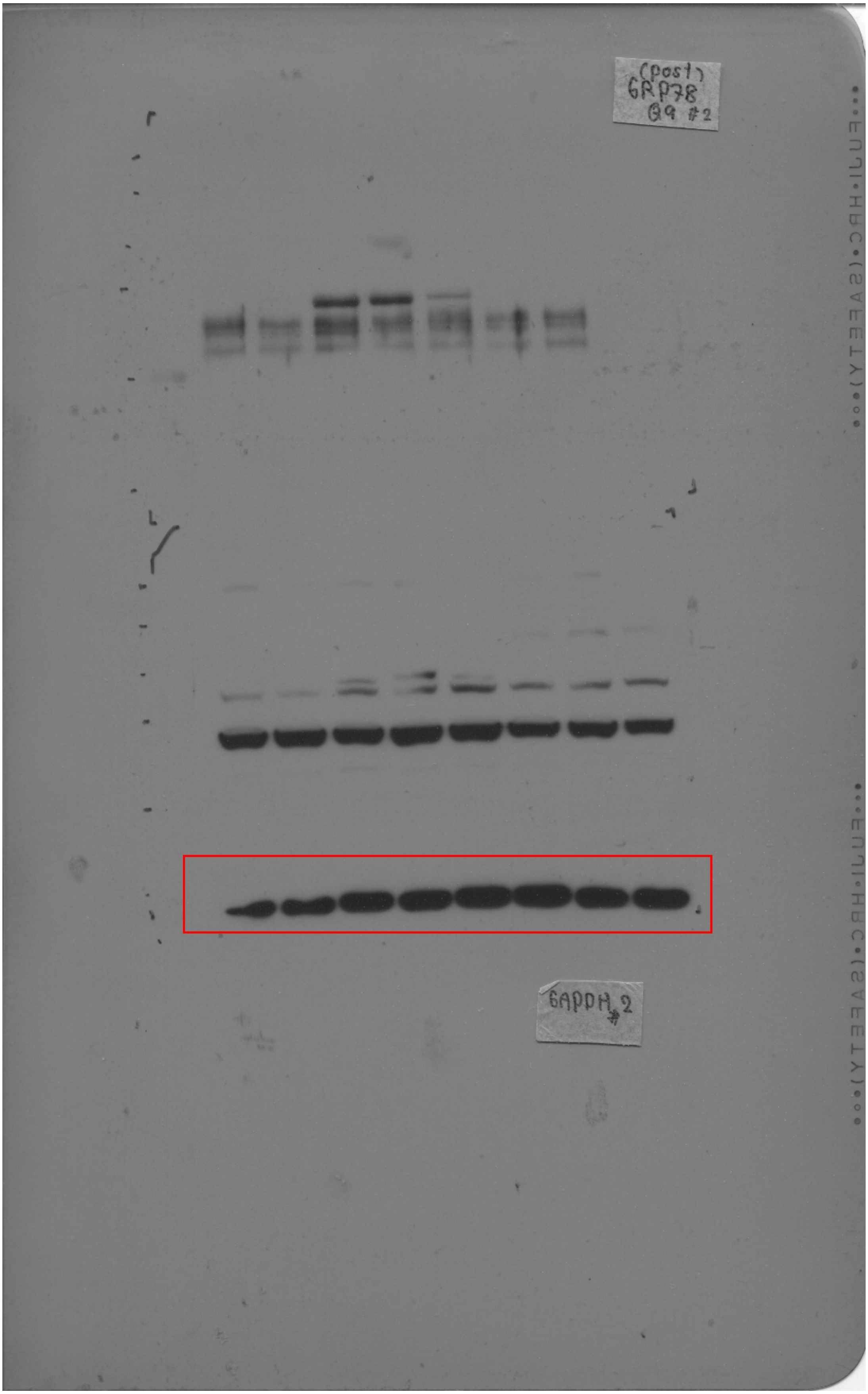

Figure 1A ; ZIKV_DENV_JEV-GAPDH (IP) #Replicate 2

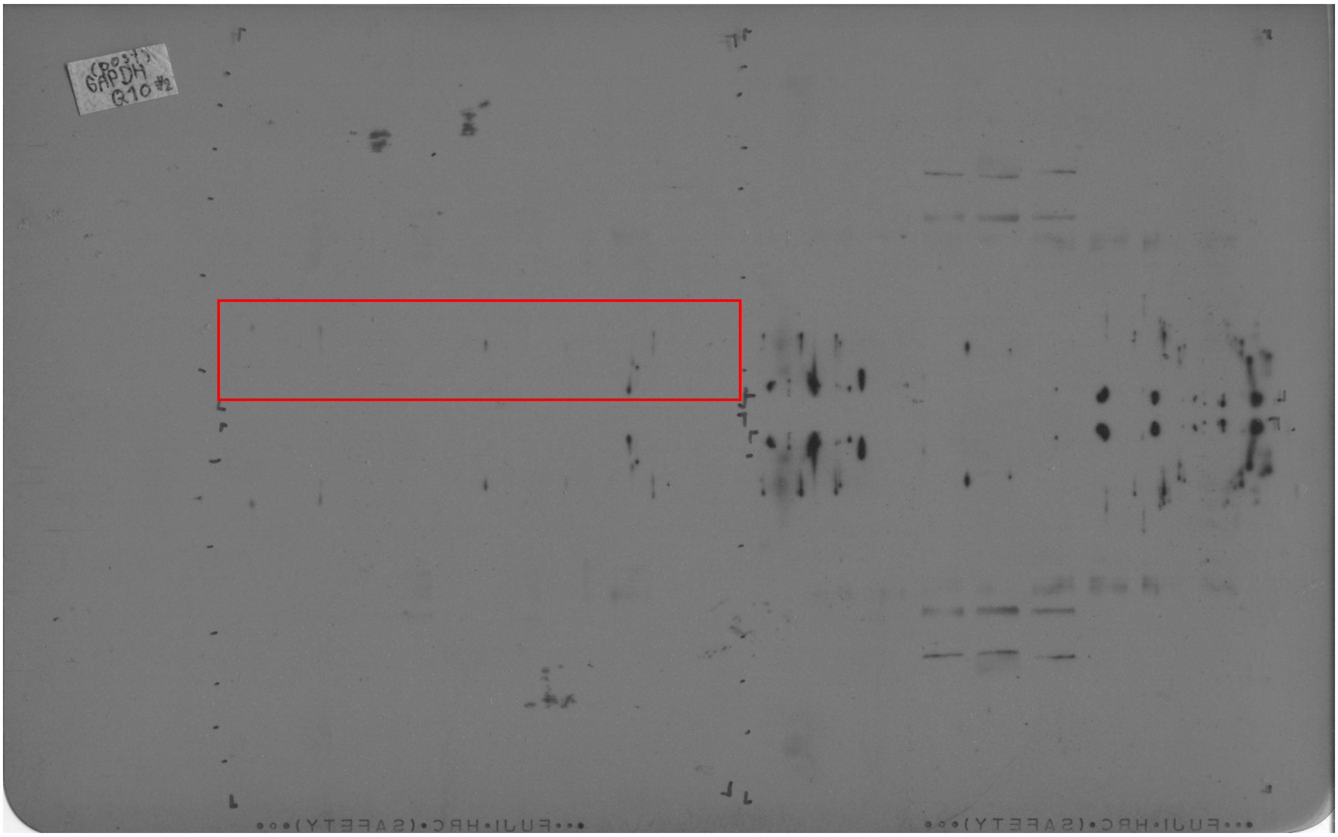

Figure 1A ; ZIKV_DENV_JEV-FASN (Input) #Replicate 3

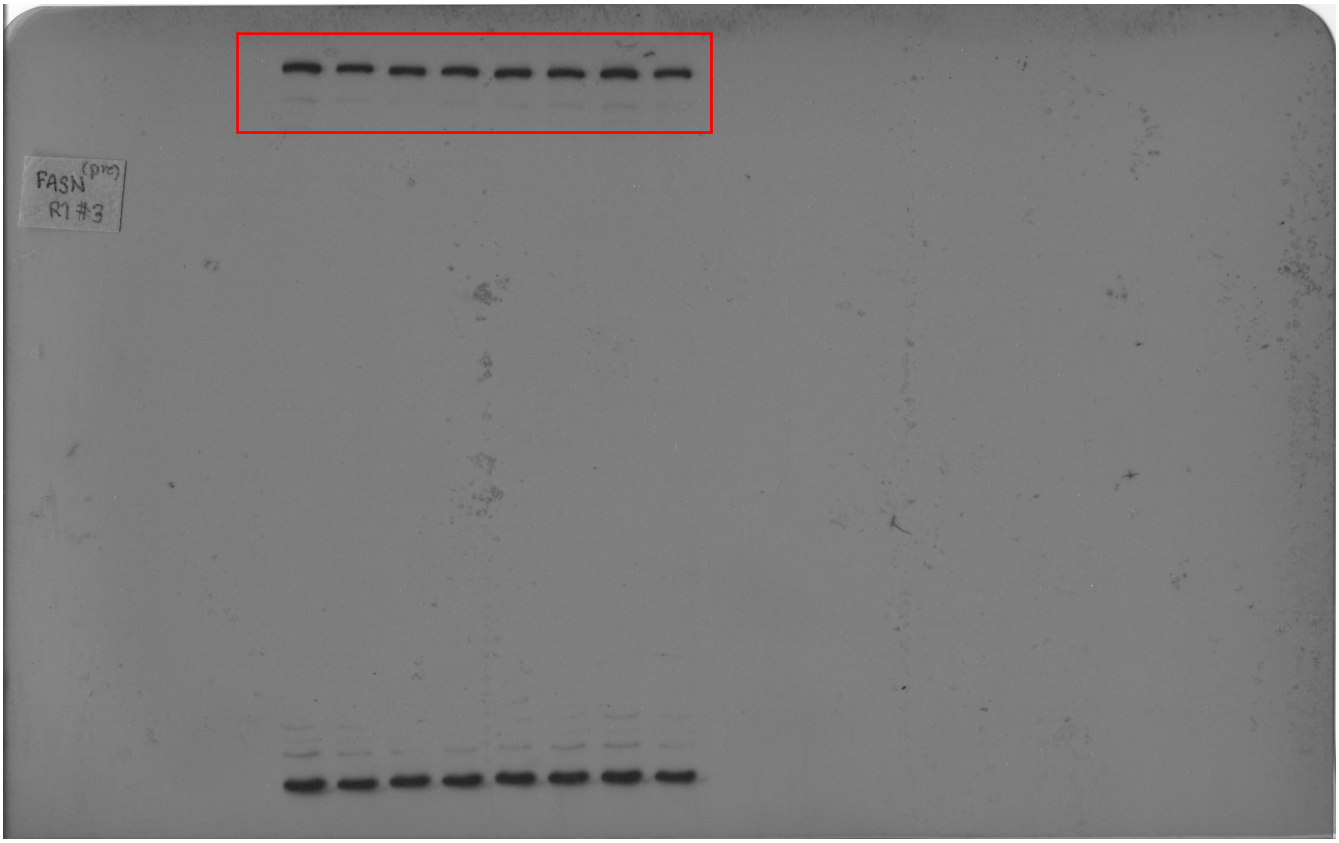

Figure 1A ; ZIKV_DENV_JEV-FASN (IP) #Replicate 3

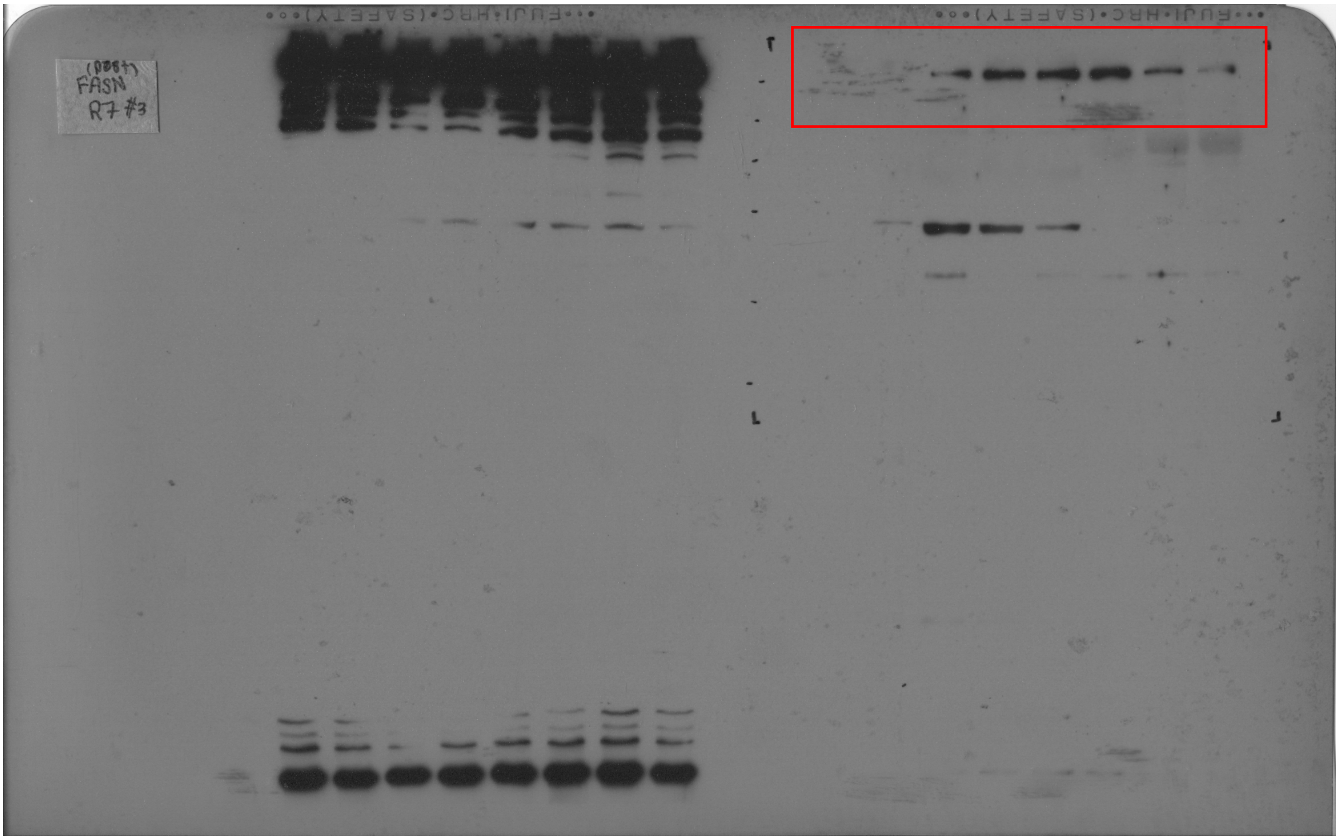

Figure 1A ; ZIKV_DENV_JEV-HSP90 (Input) #Replicate 3

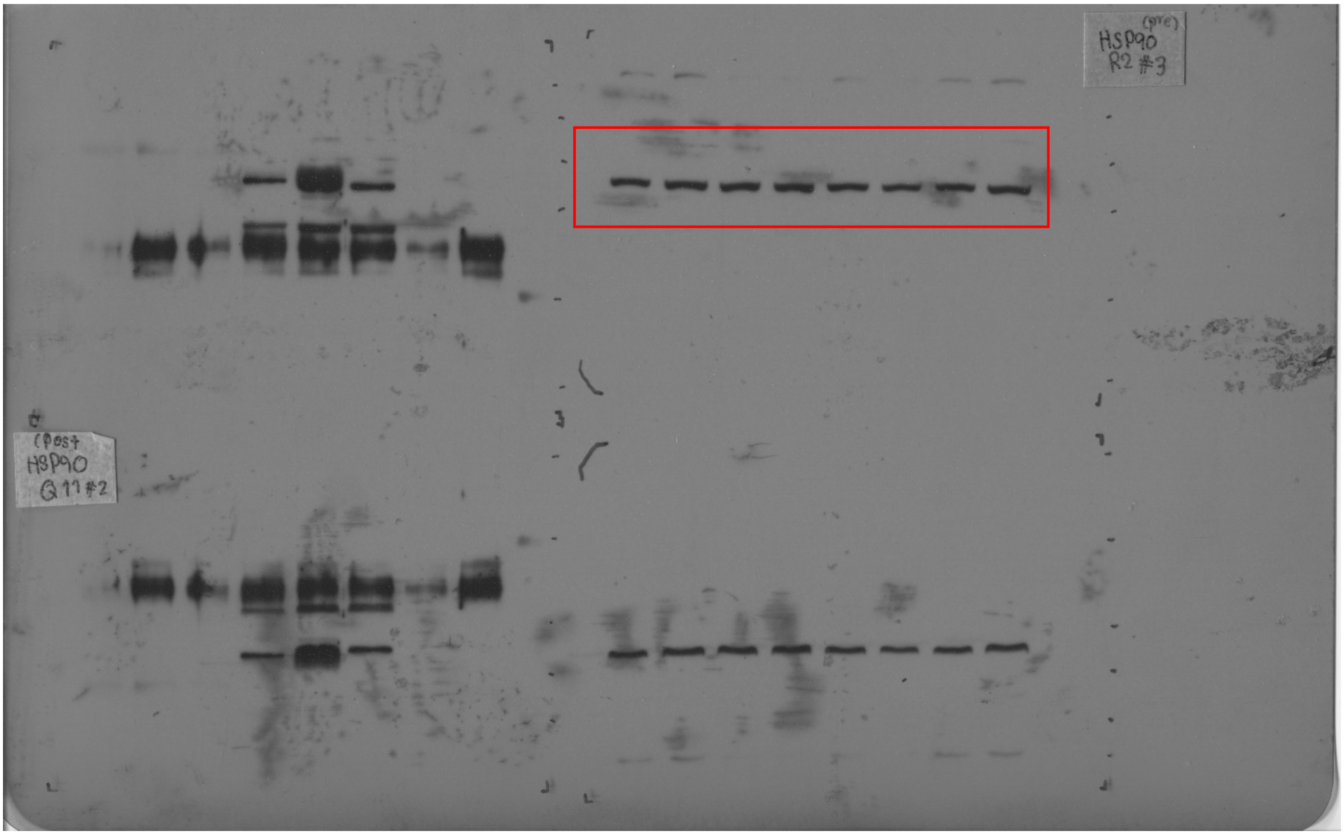

Figure 1A ; ZIKV_DENV_JEV-HSP90 (IP) #Replicate 3

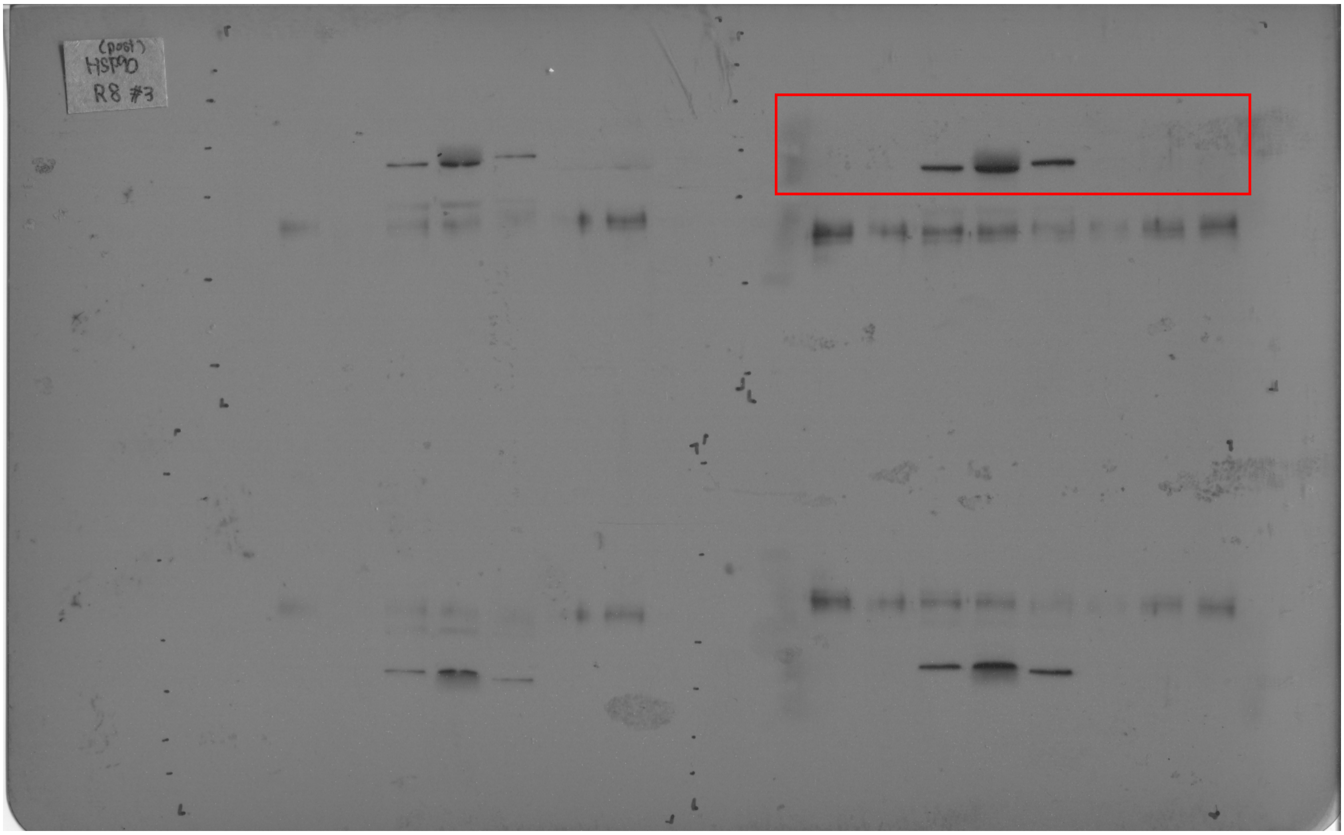

Figure 1A ; ZIKV_DENV_JEV-GRP78 (Input) #Replicate 3

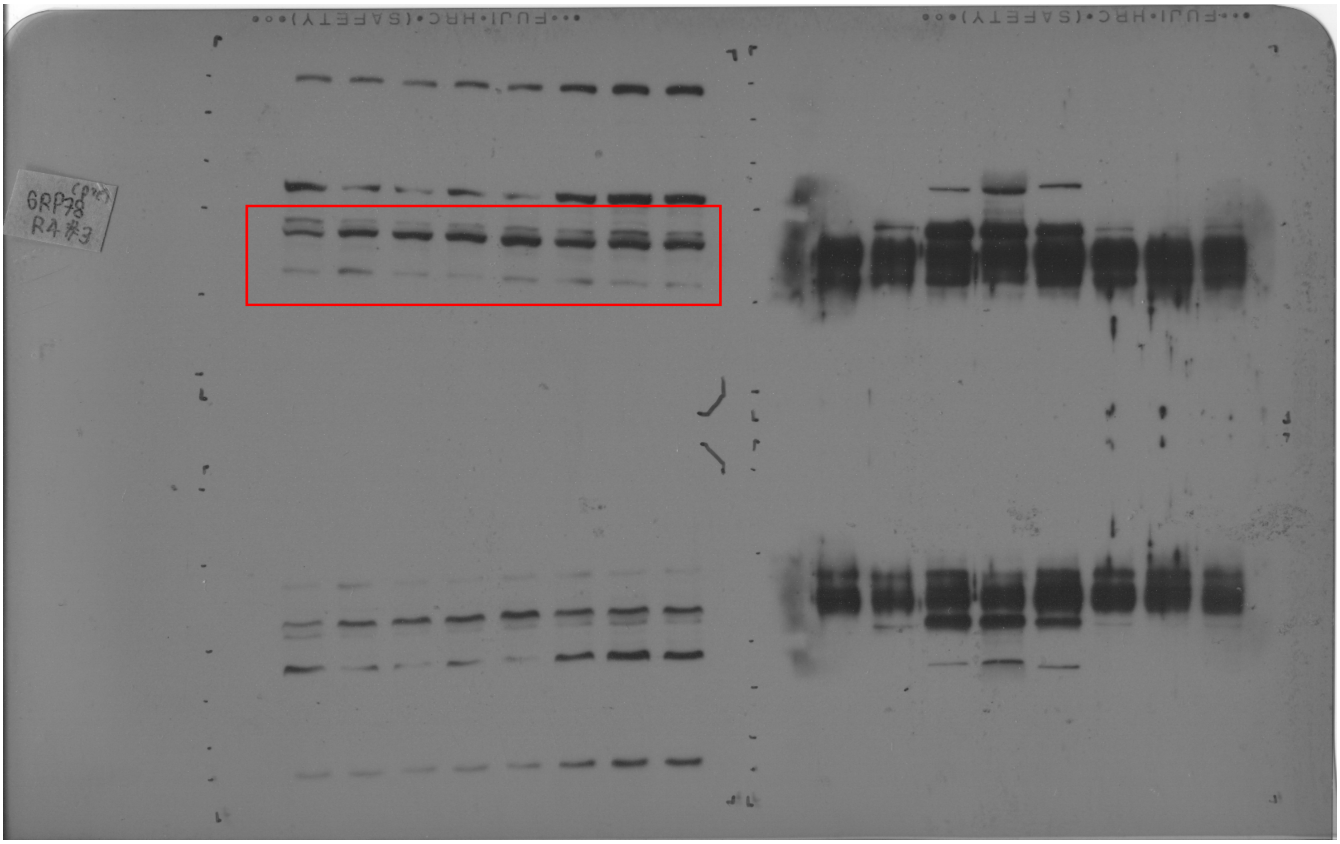

Figure 1A ; ZIKV_DENV_JEV-GRP78 (IP) #Replicate 3

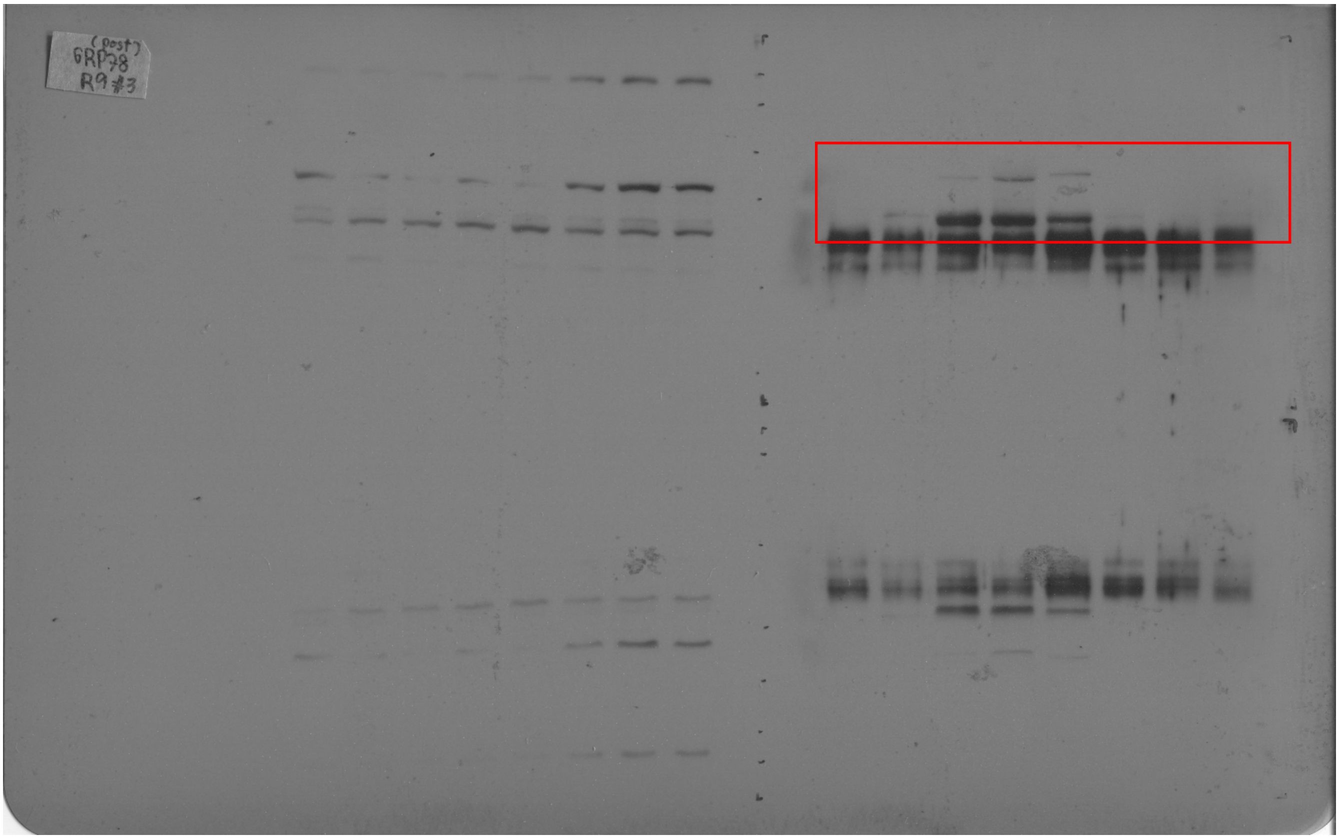

Figure 1A ; ZIKV_DENV_JEV-GFP (Input) #Replicate 3

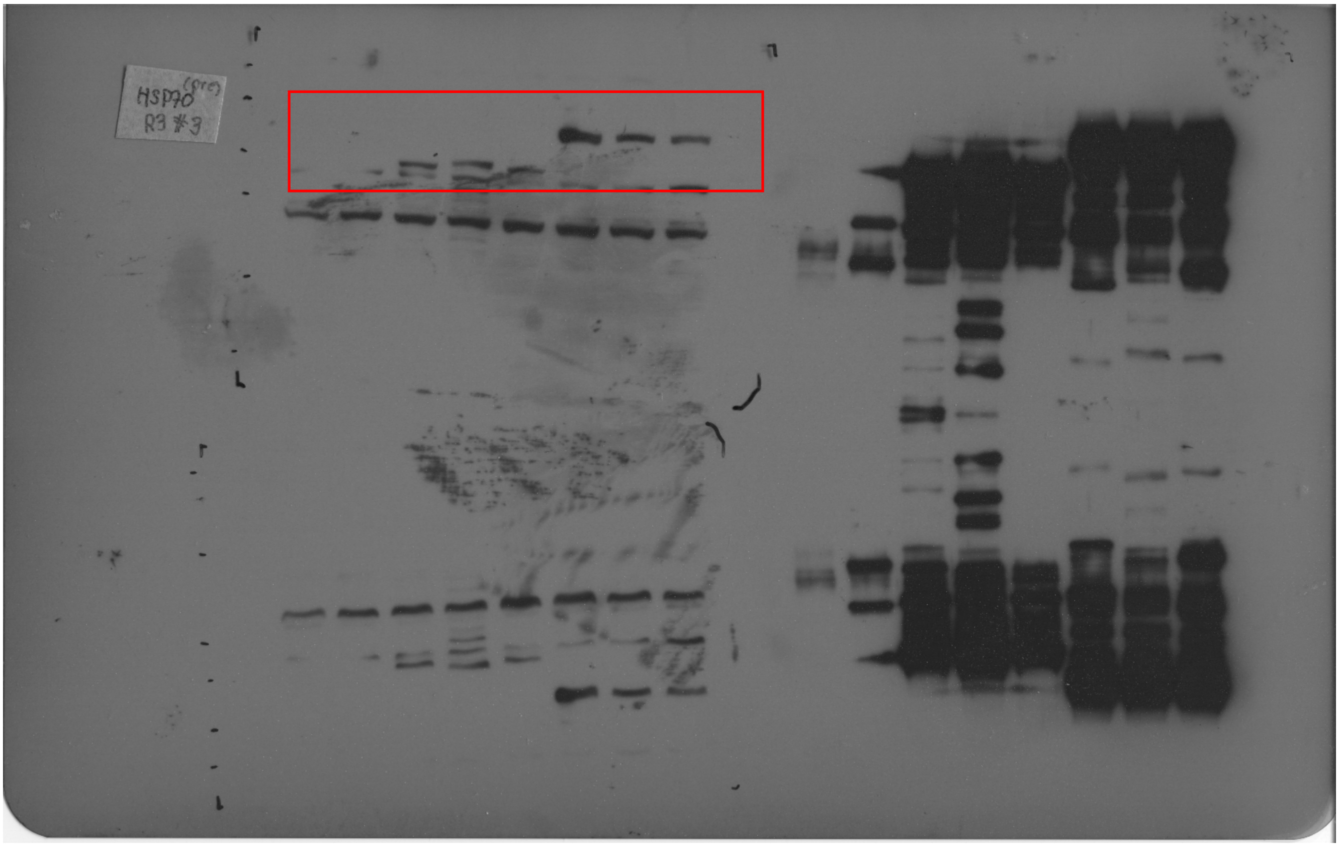

Figure 1A ; ZIKV_DENV_JEV-GFP (IP) #Replicate 3

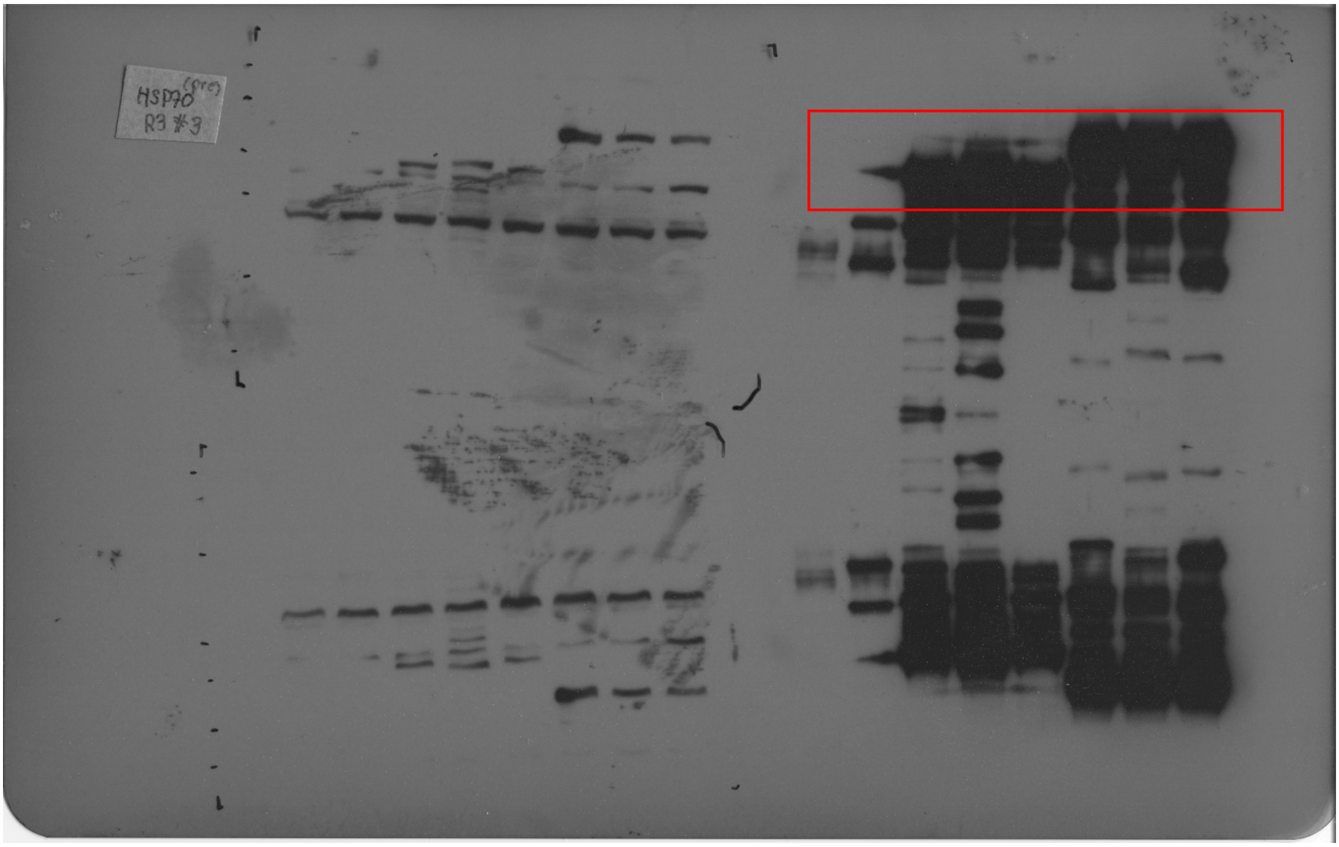

Figure 1A ; ZIKV_DENV_JEV-GAPDH (IP and Input) #Replicate 3

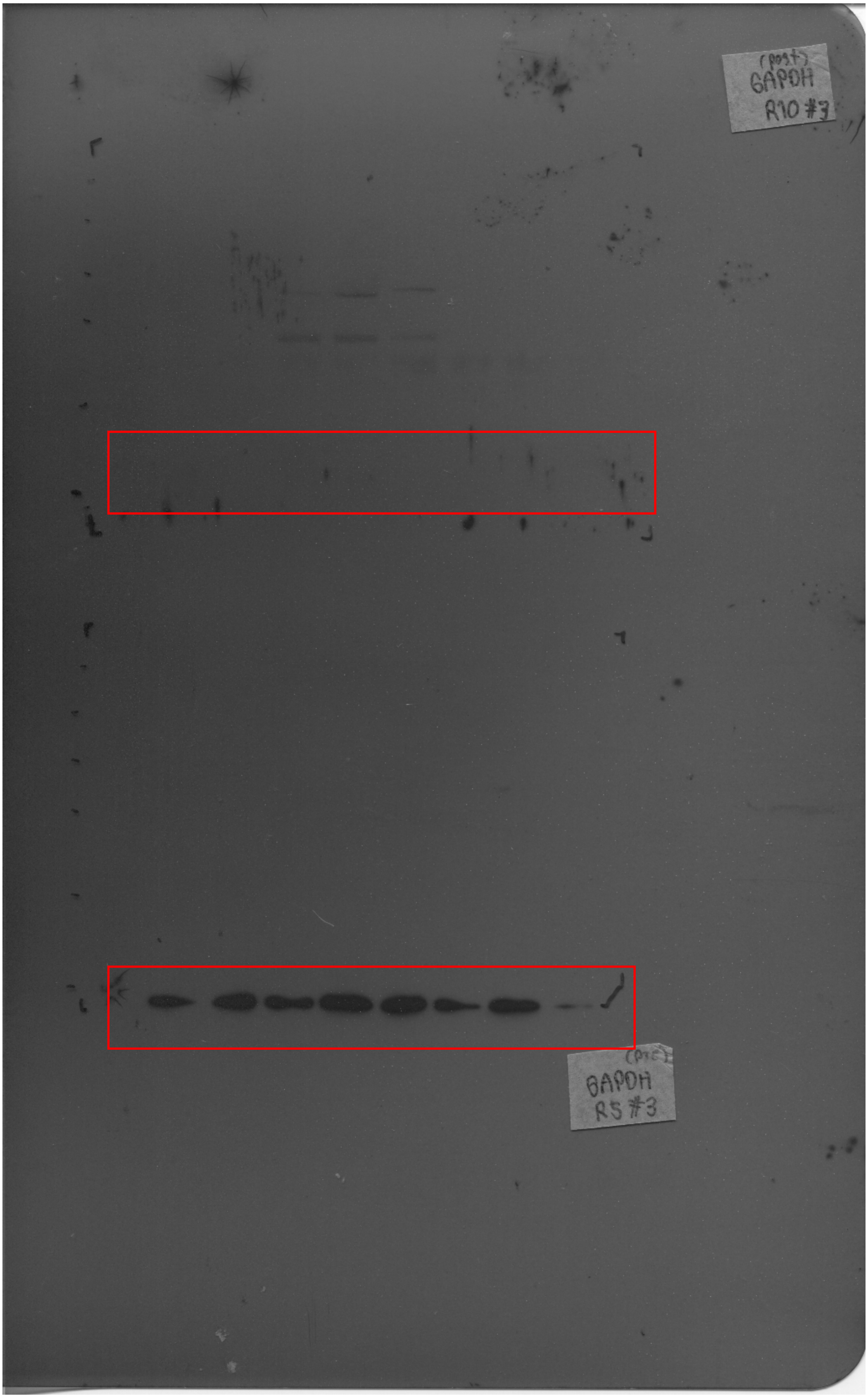

Figure 1B ; DENV-NS3 (IP)

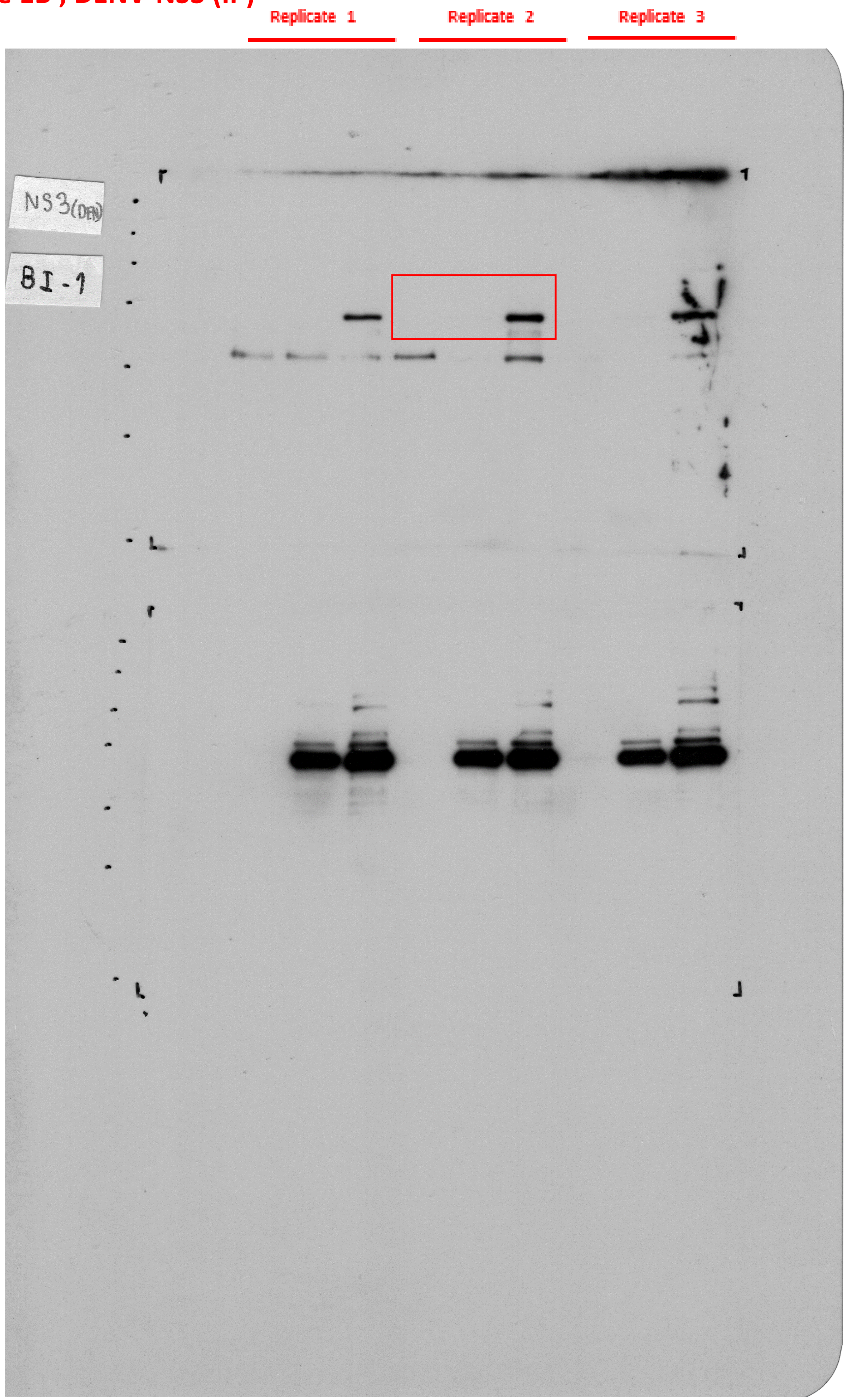

Figure 1B ; JEV-NS3 (IP)

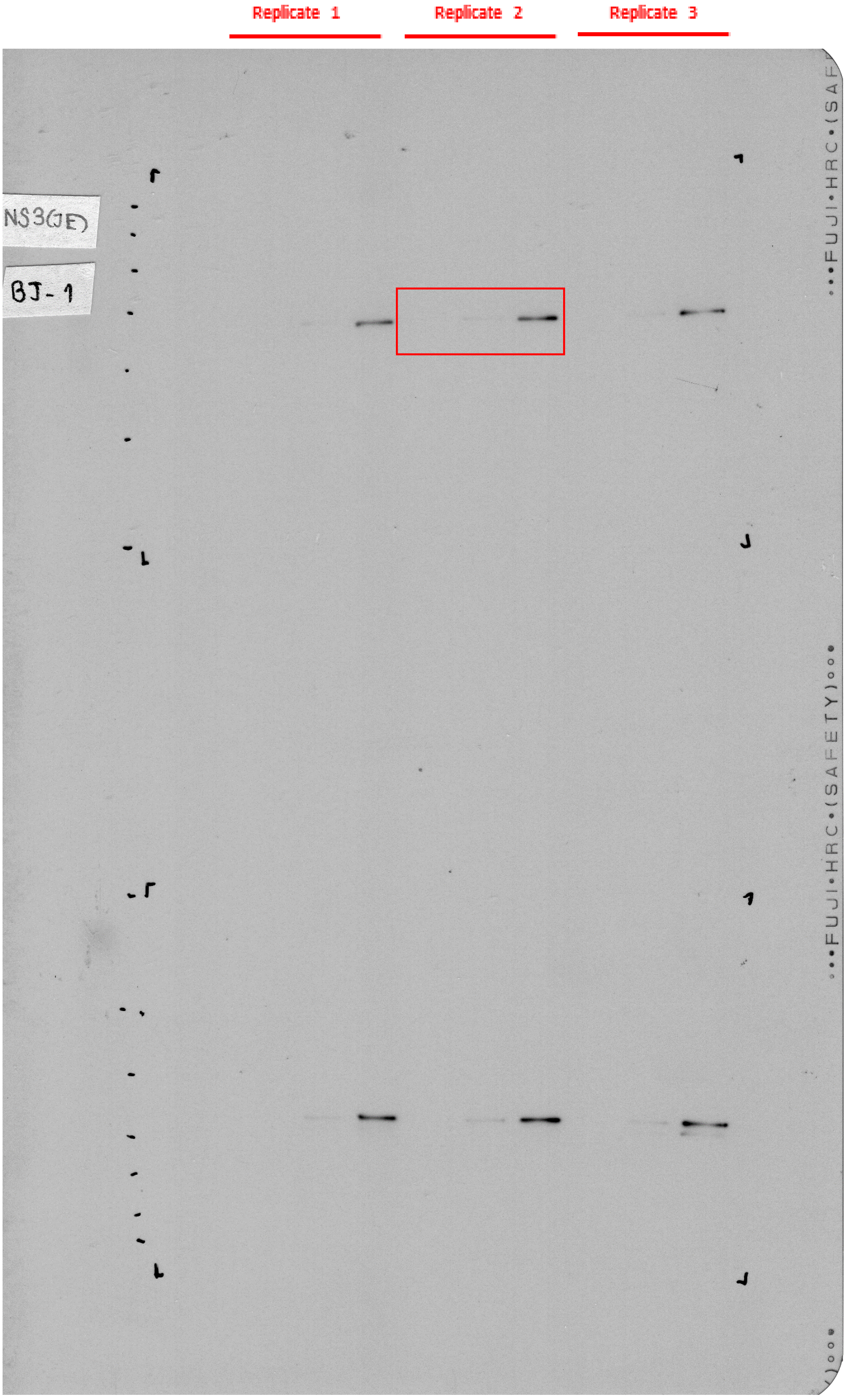

Figure 1B ; ZIKV-NS3 (IP)

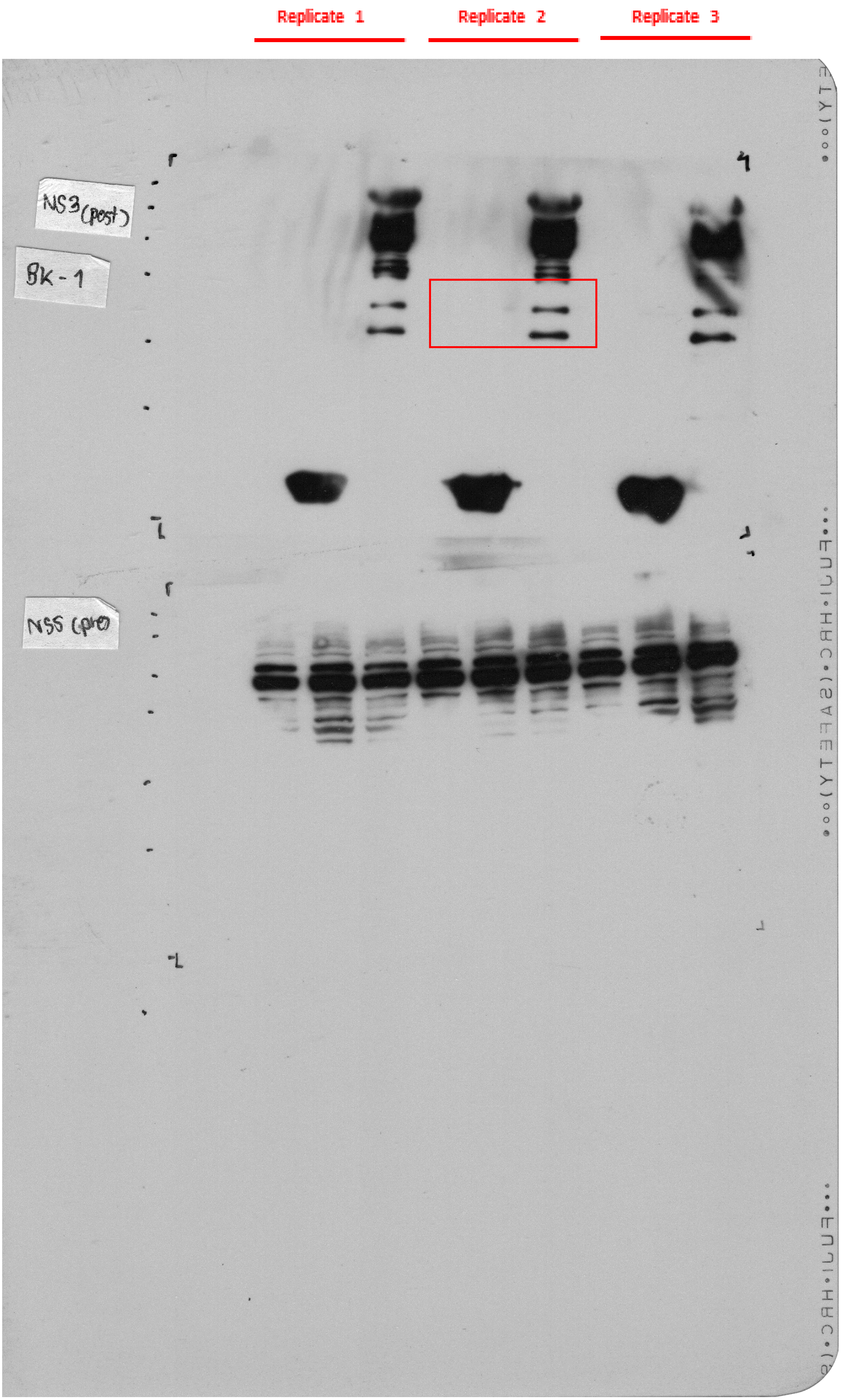

Figure 1B ; DENV-NS3 (Input)

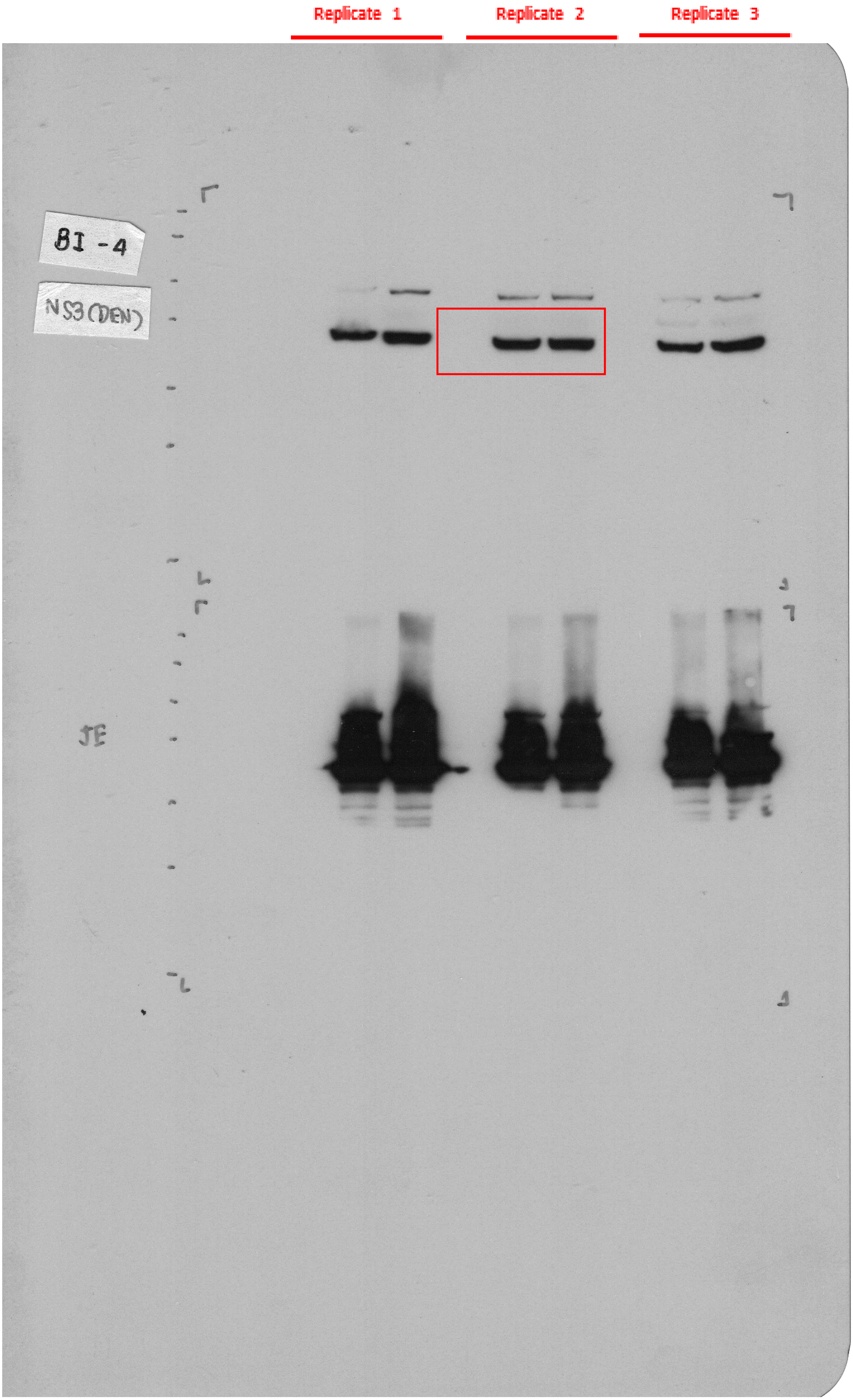

Figure 1B ; JEV-NS3 (Input)

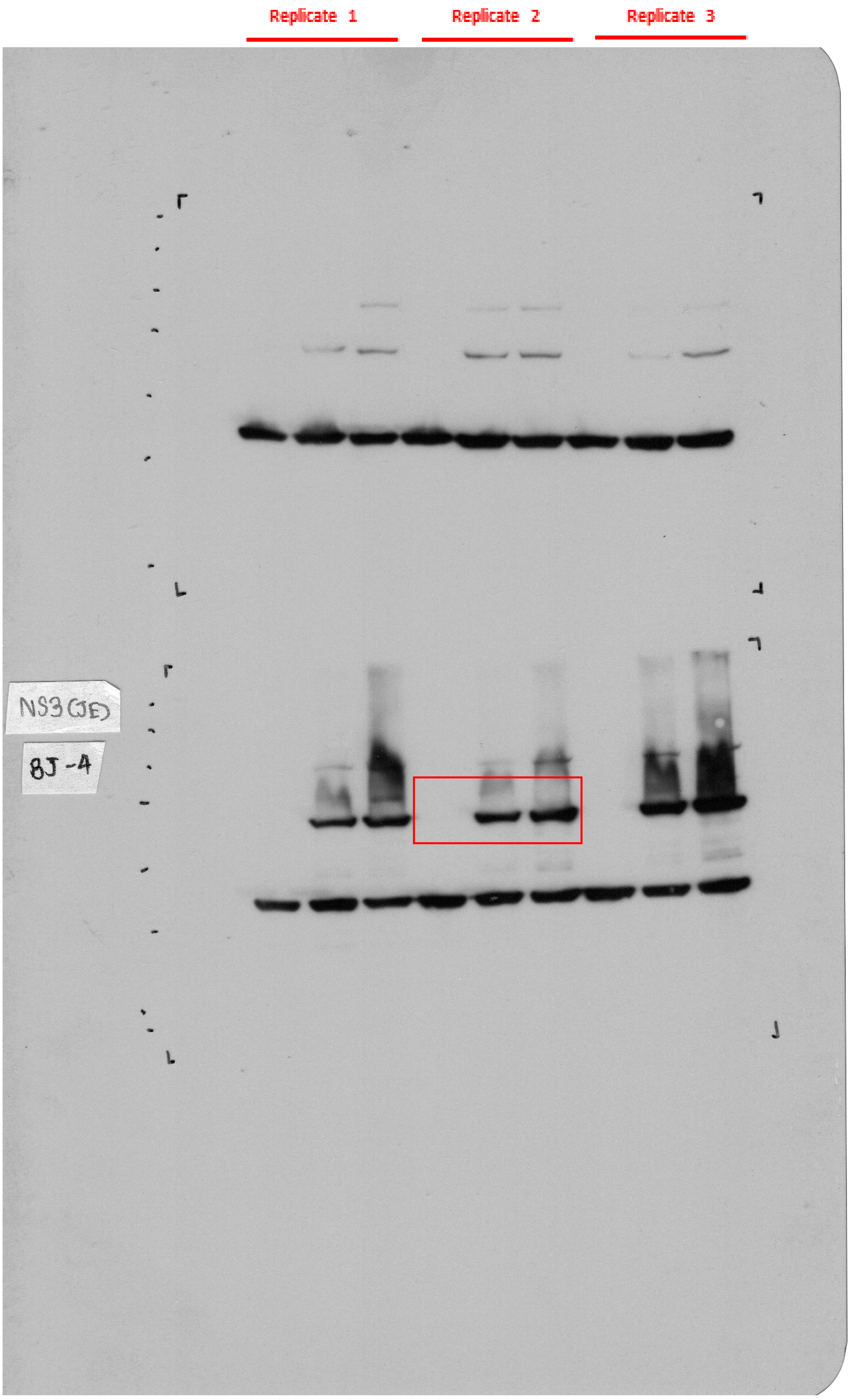

Figure 1B ; ZIKV-NS3 (Input)

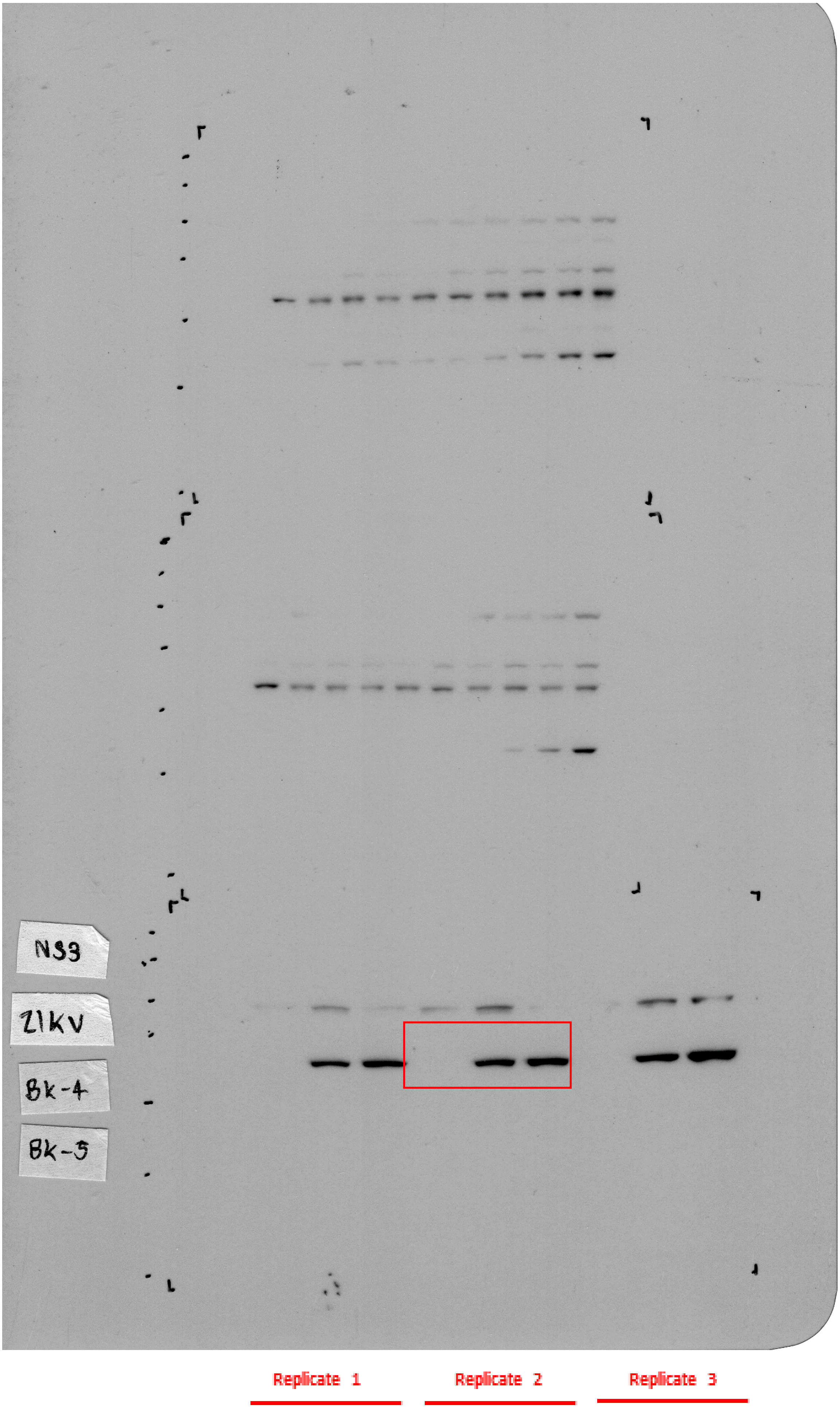

Figure 1B ; DENV-NS5 (upper, IP) and JEV-NS5 (lower, IP)

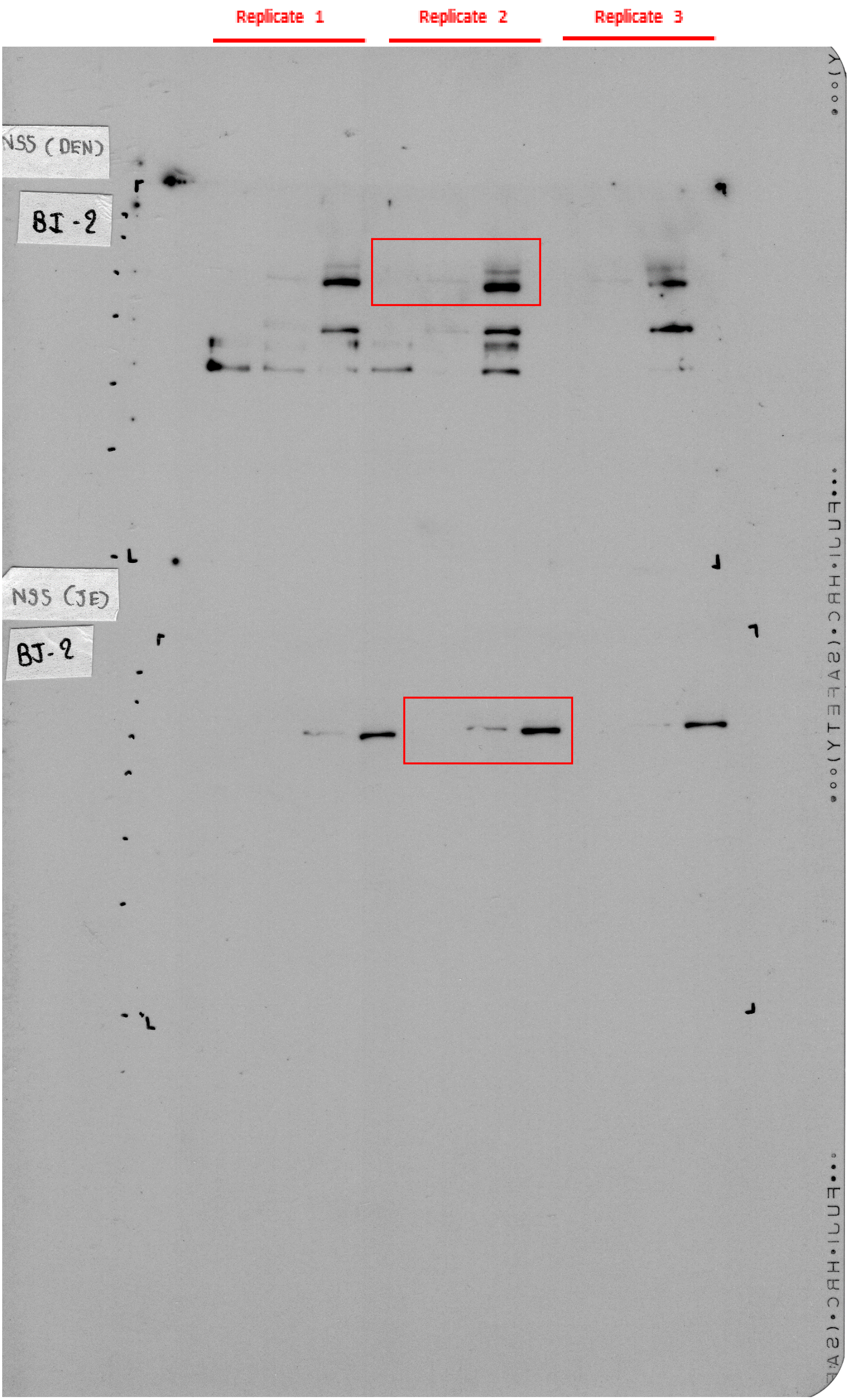

Figure 1B ; ZIKV-NS5 (IP)

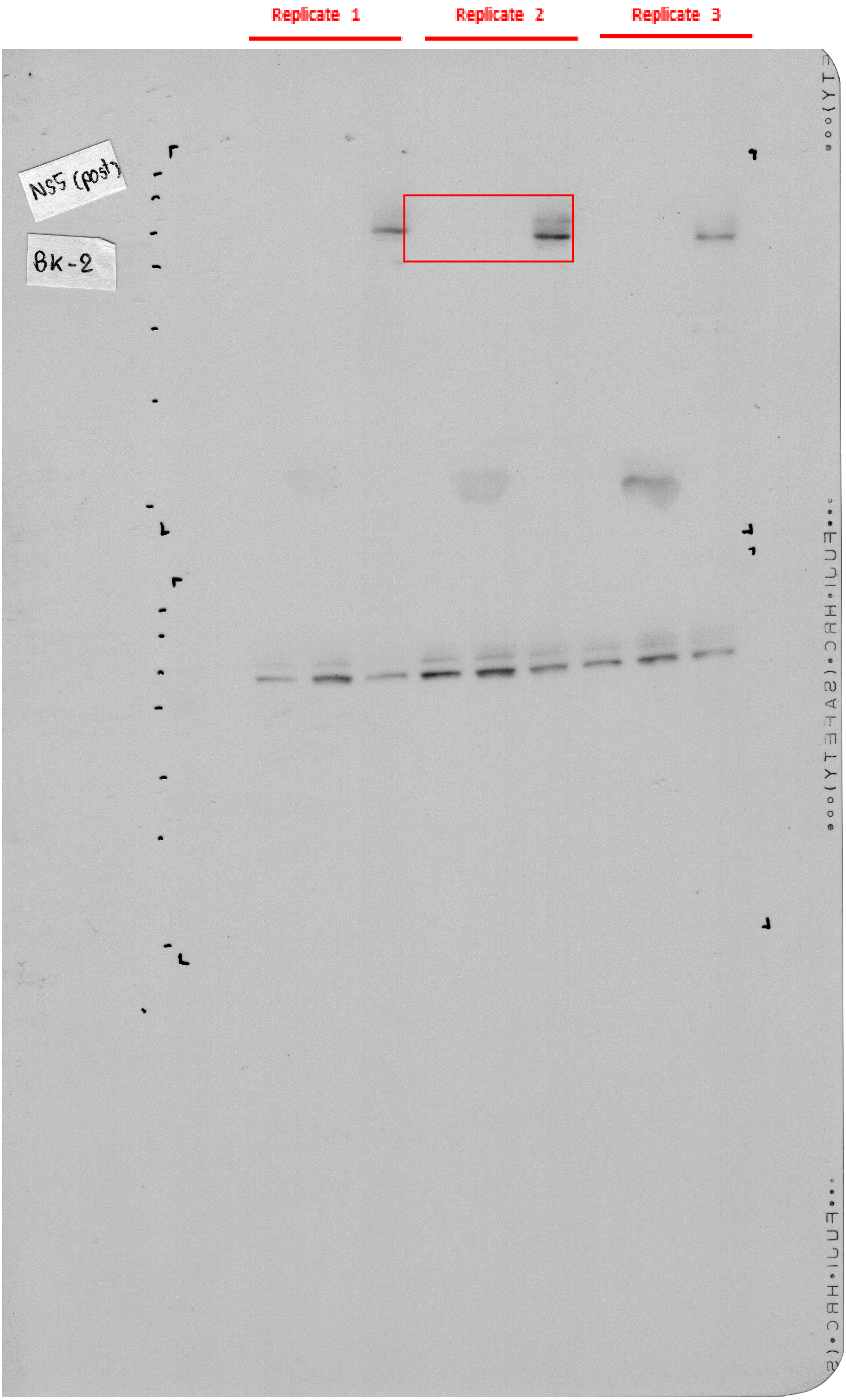

Figure 1B ; DENV-NS5 (upper, Input) and JEV-NS5 (lower, Input)

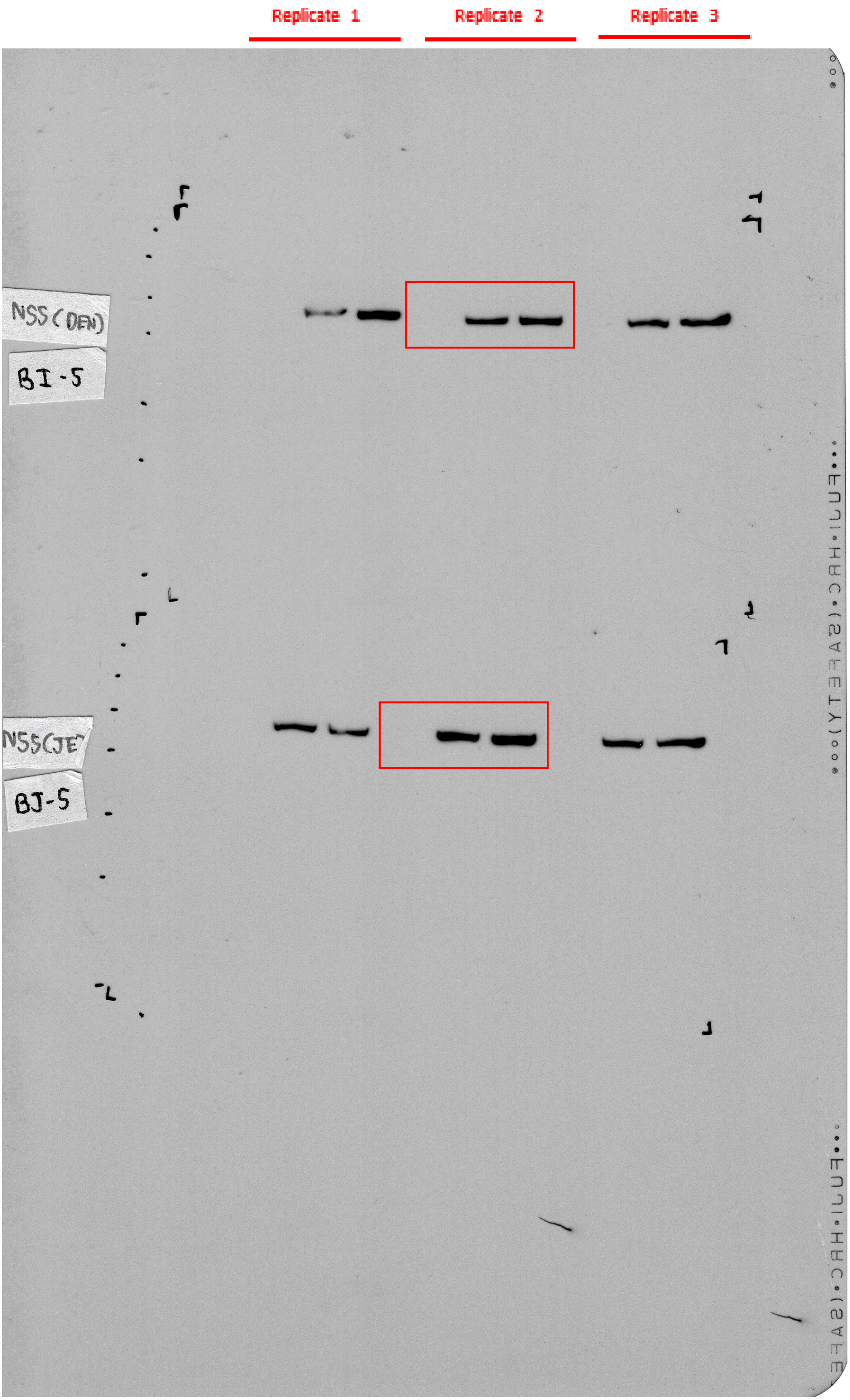

Figure 1B ; ZIKV-NS5 (Input)

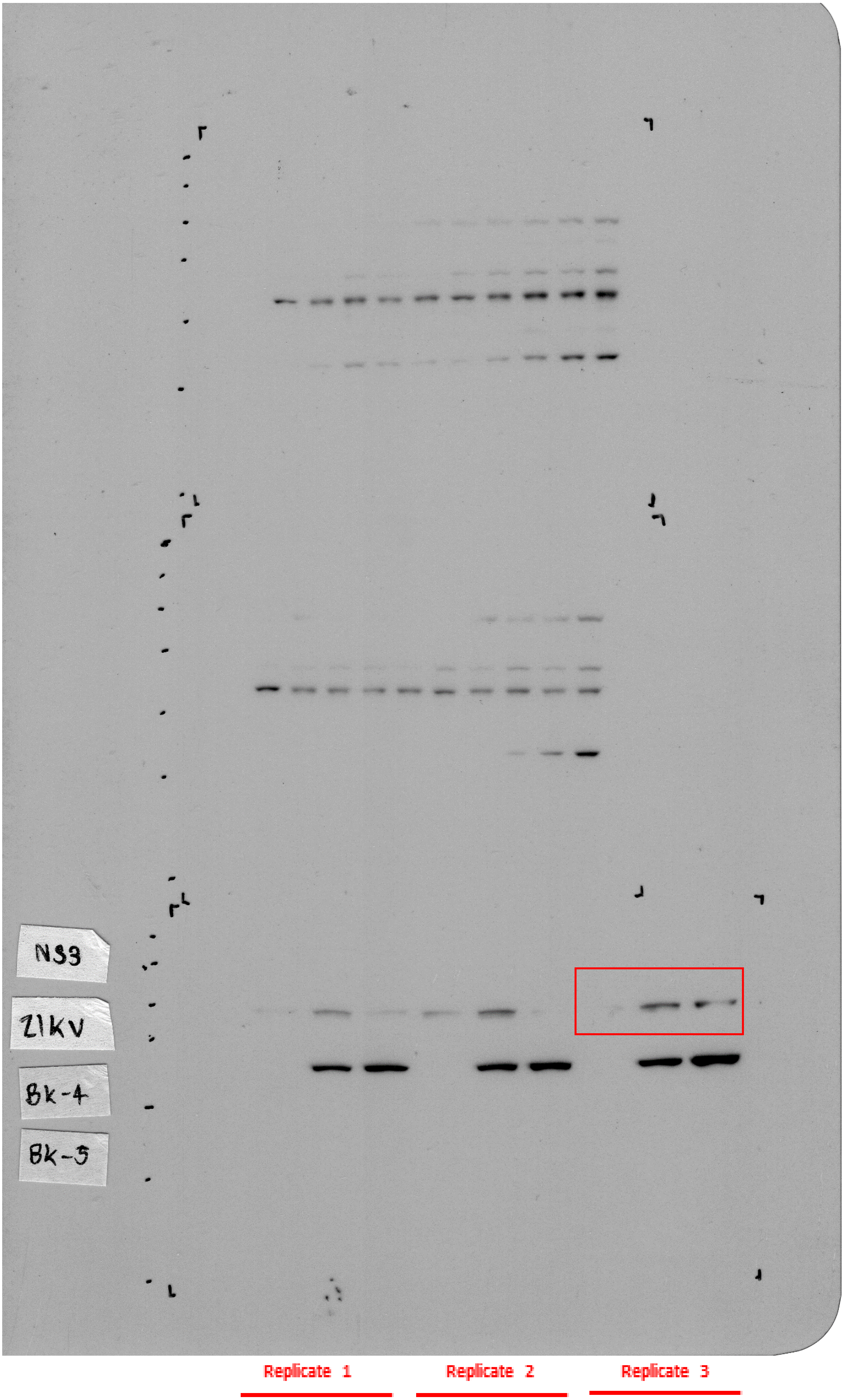

Figure 1B ; DENV-GFP (upper, IP) and JEV-GFP (lower, IP)

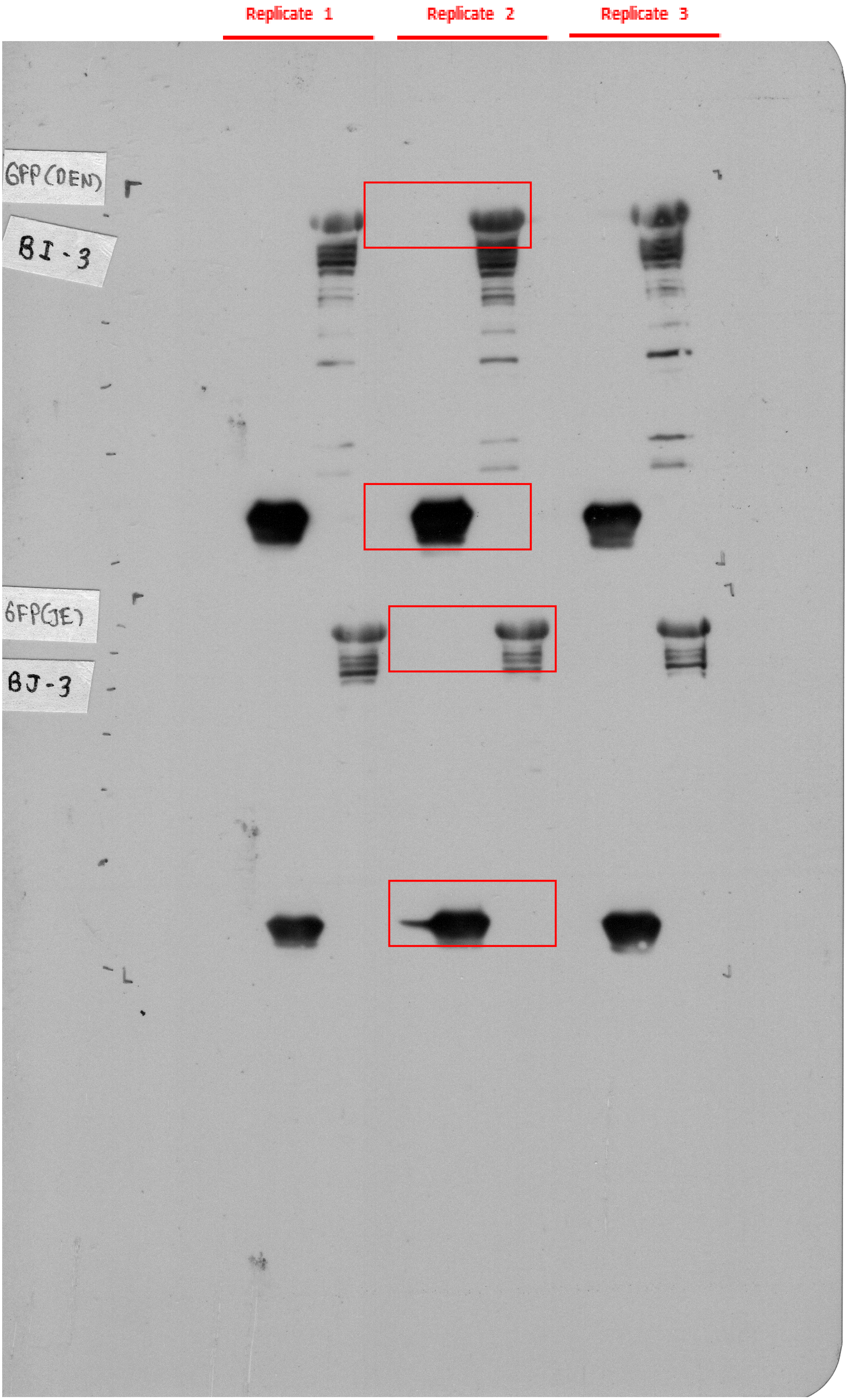

Figure 1B ; ZIKV-GFP (IP)

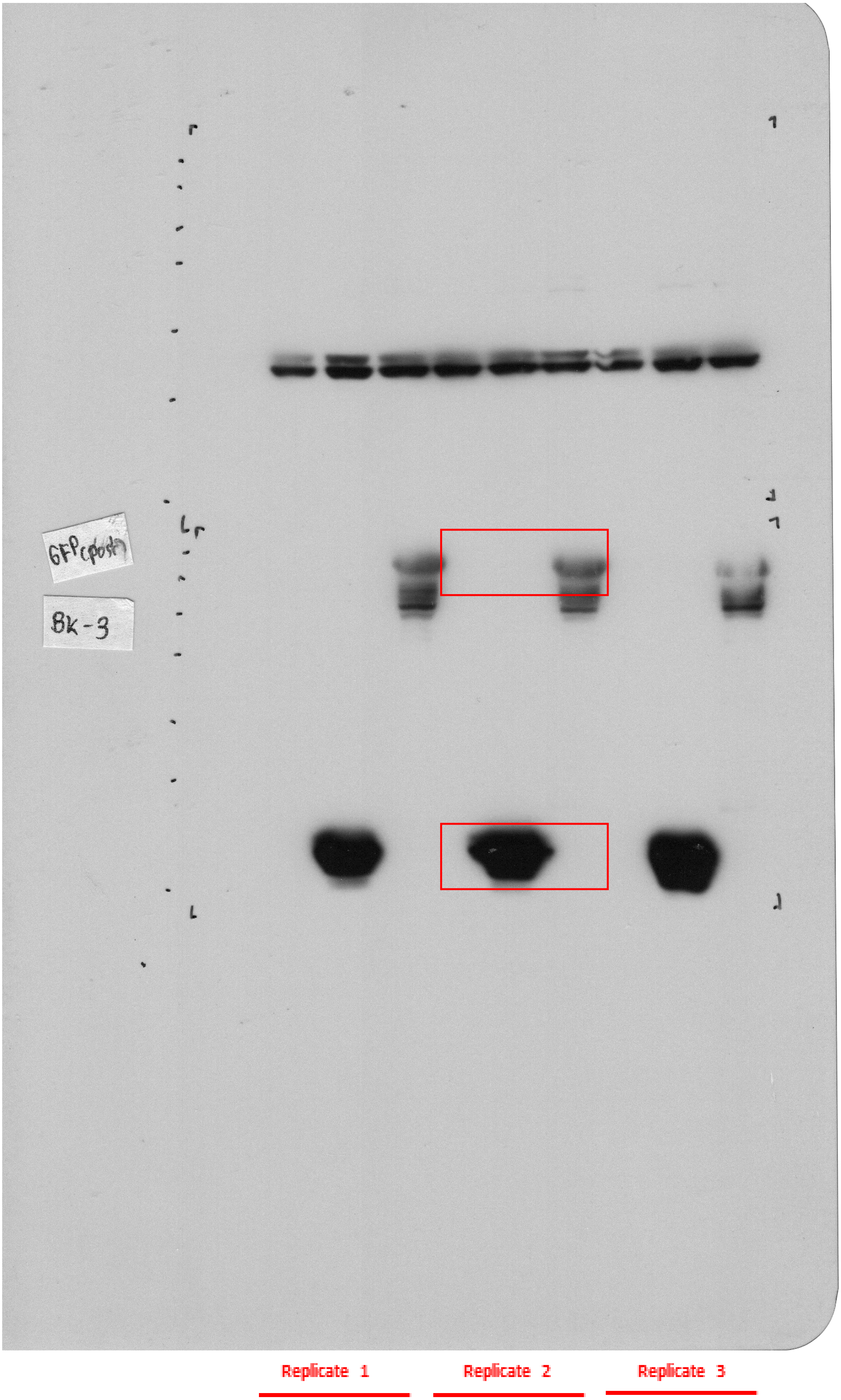

Figure 1B ; DENV-GFP (upper, Input) and JEV-GFP (lower, Input)

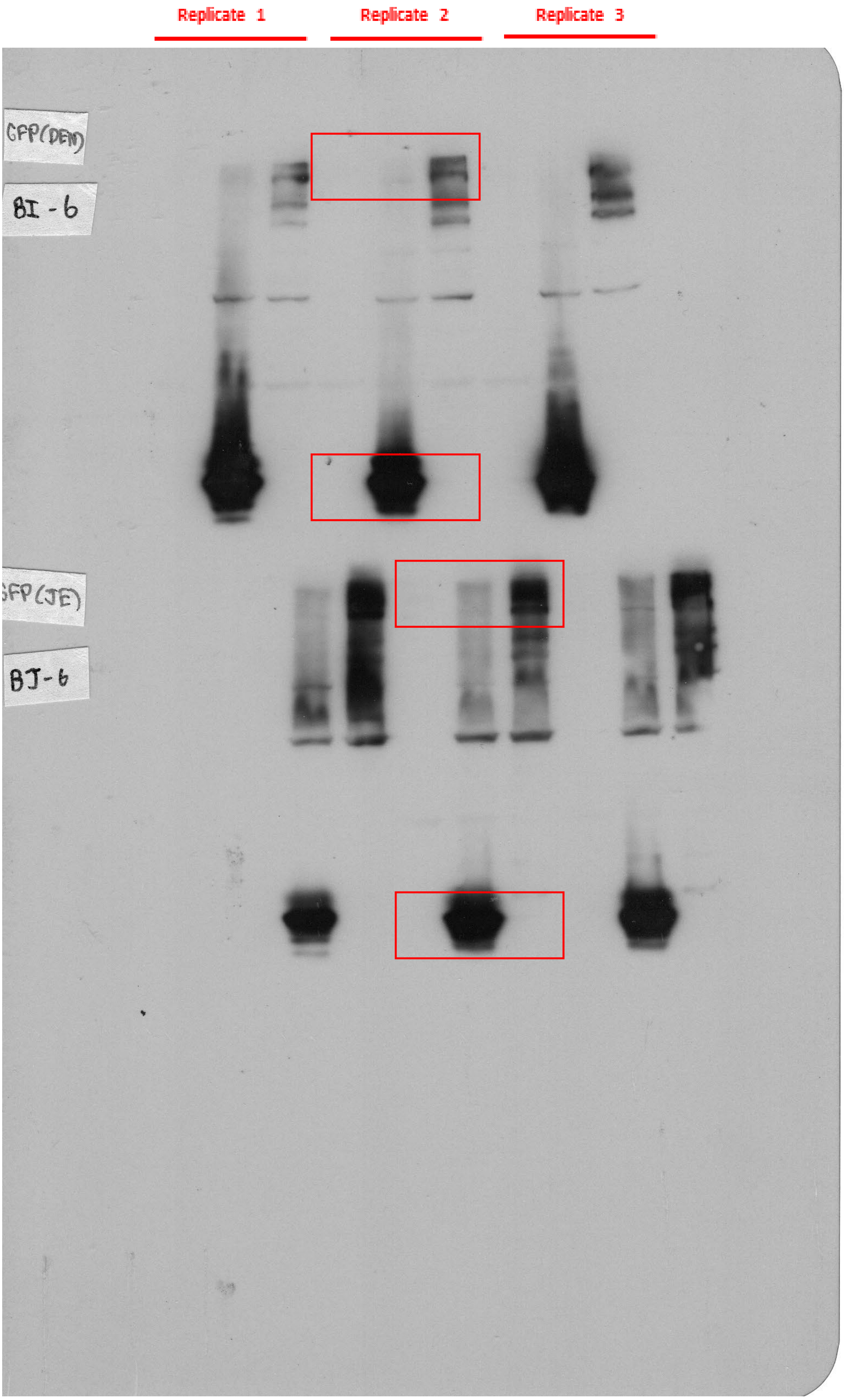

Figure 1B ; ZIKV-GFP (Input)

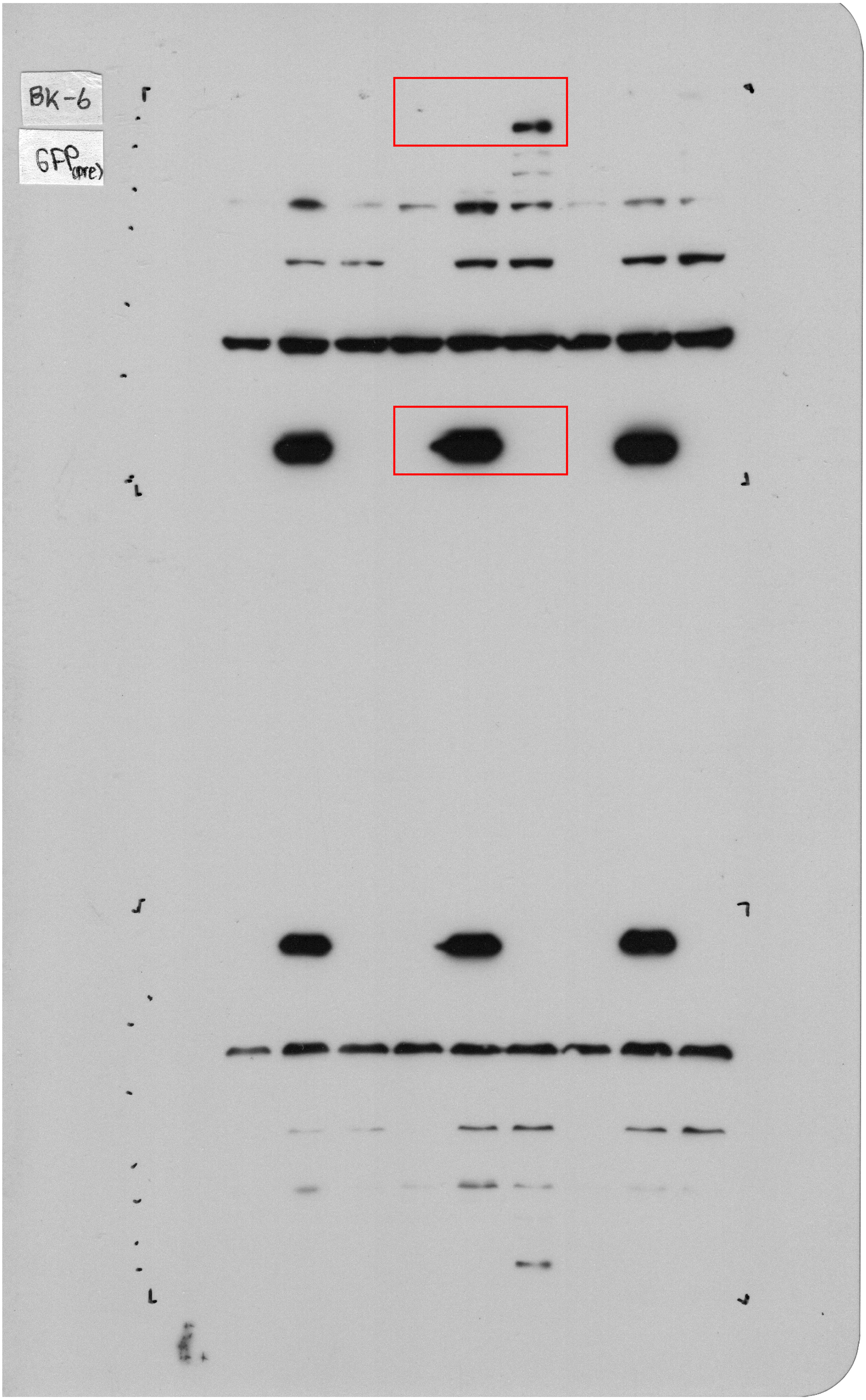

Figure 1B ; ZIKV-GFP (Input) # Replicate 1 and 3

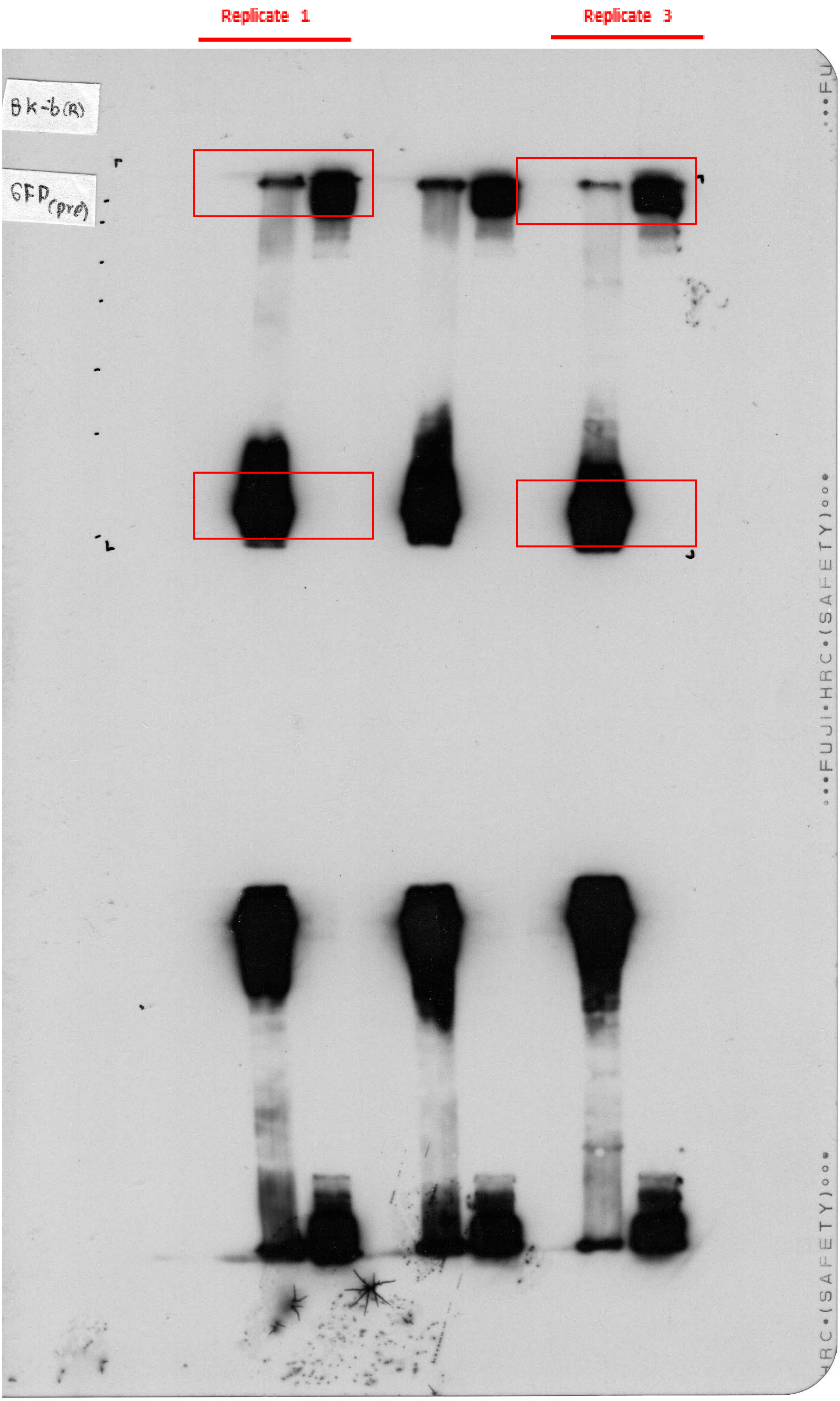

Figure 1B ; DENV-actin (Input)

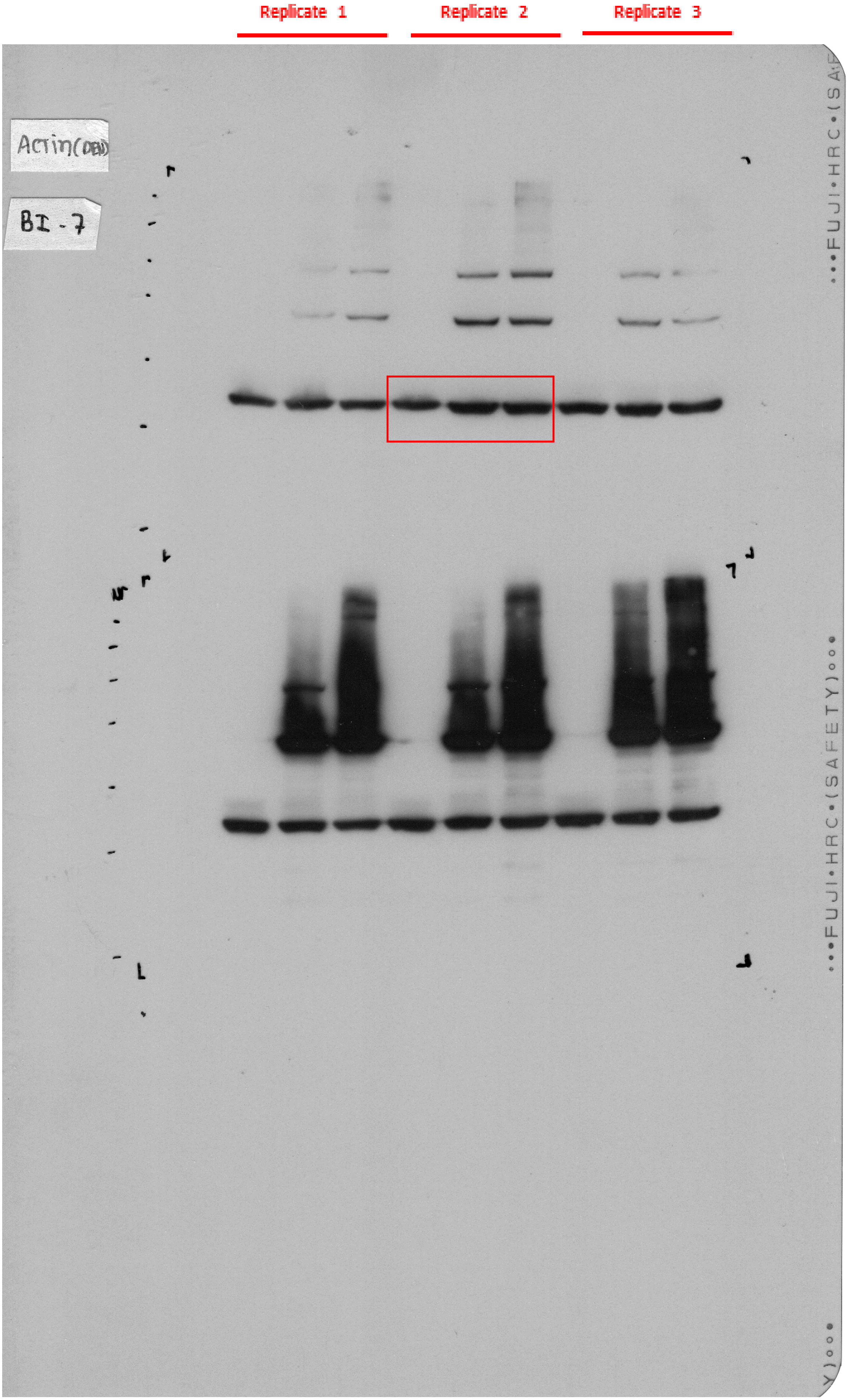

Figure 1B ; JEV-actin (Input)

Replicate 1 Replicate 2 Replicate 3

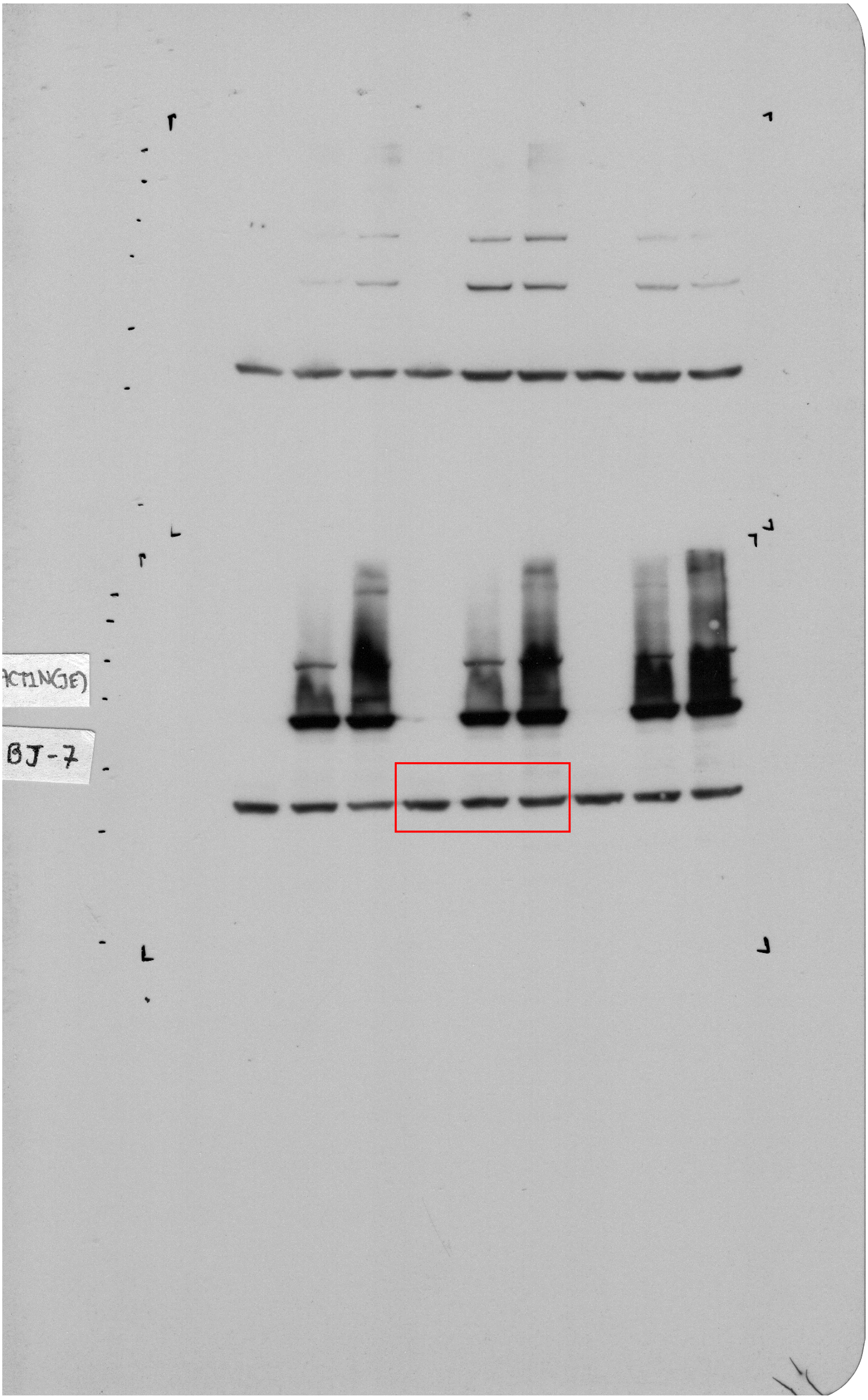

Figure 1B ; ZIKV-actin (Input)

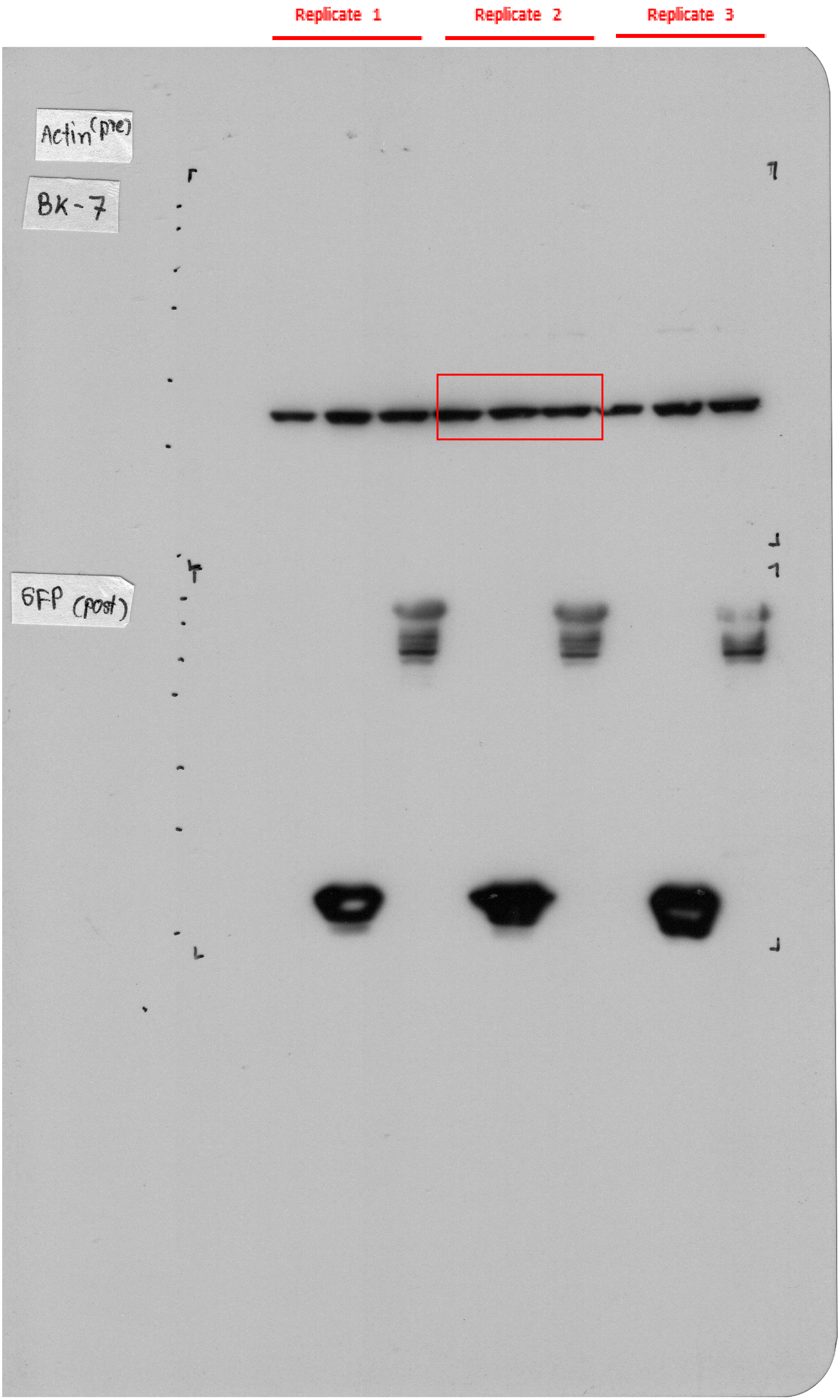

Figure 2A ; DENV_FASN (Input)

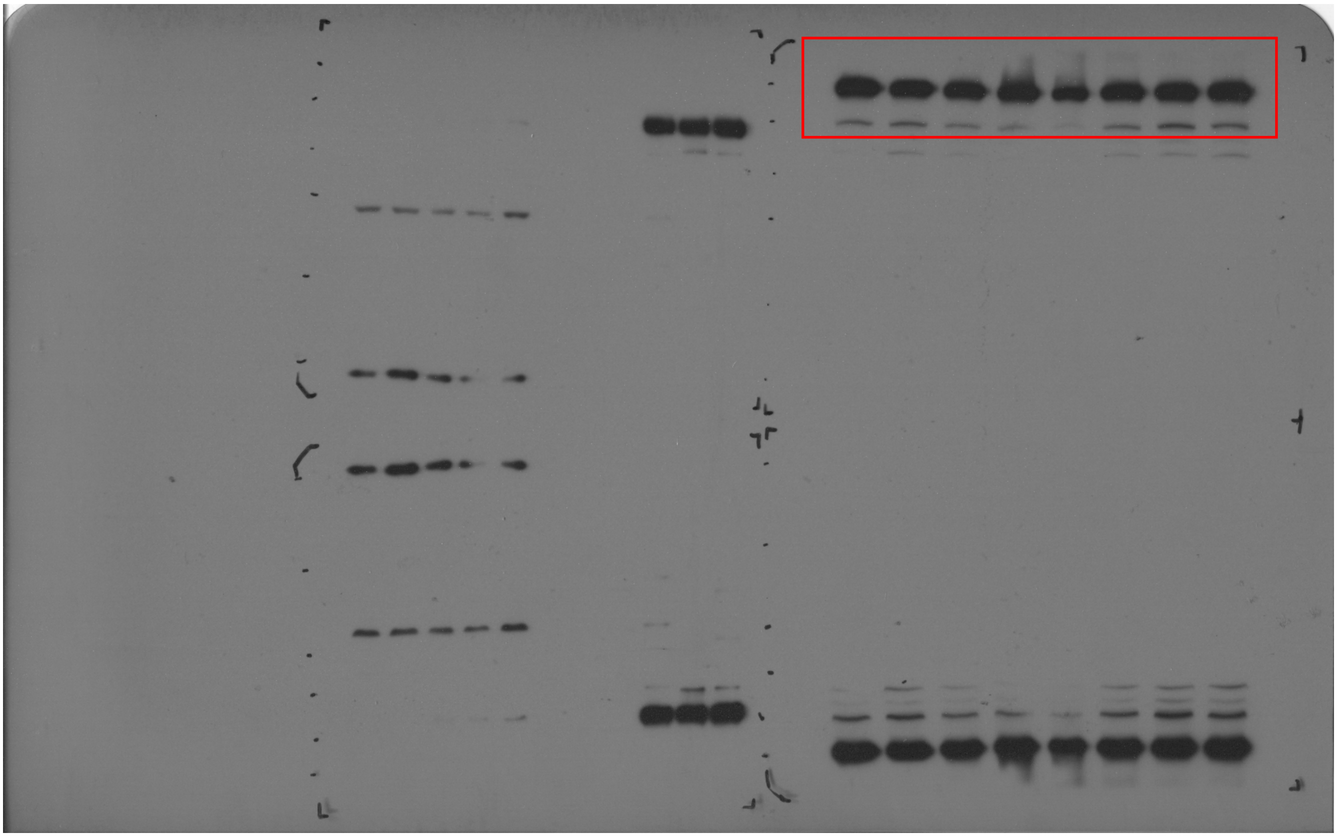

Figure 2A ; DENV_FASN (IP)

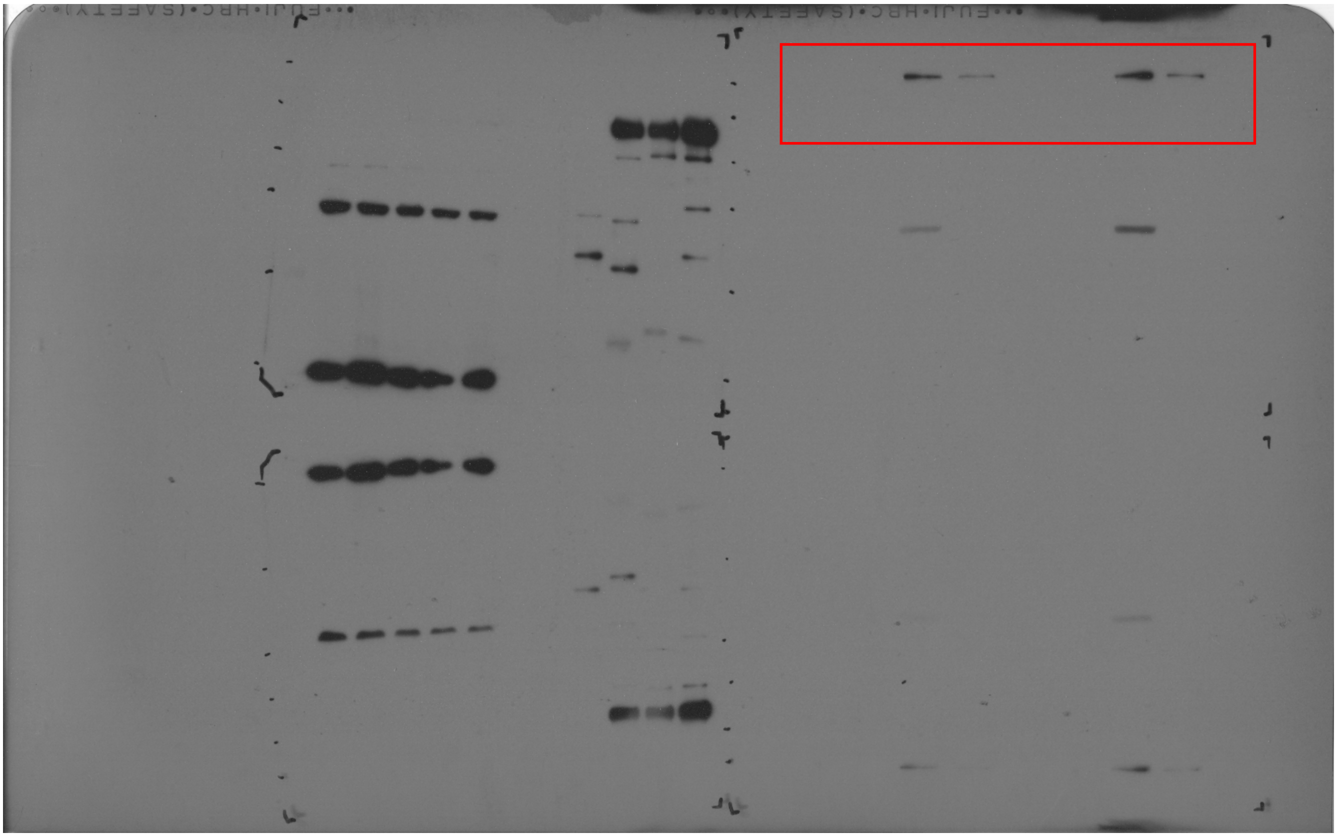

Figure 2A ; DENV_GFP (Input)

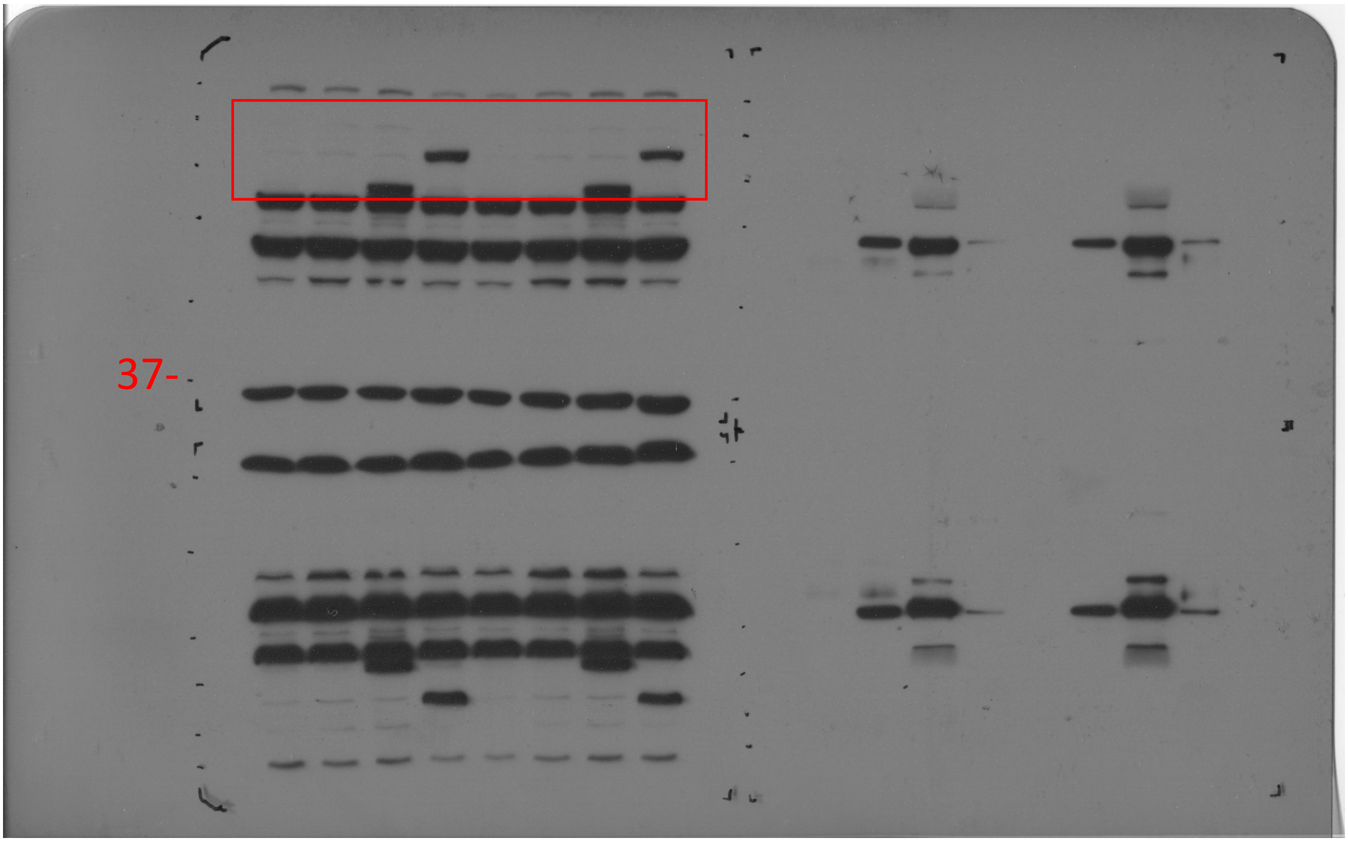

Figure 2A ; DENV_GFP (IP)

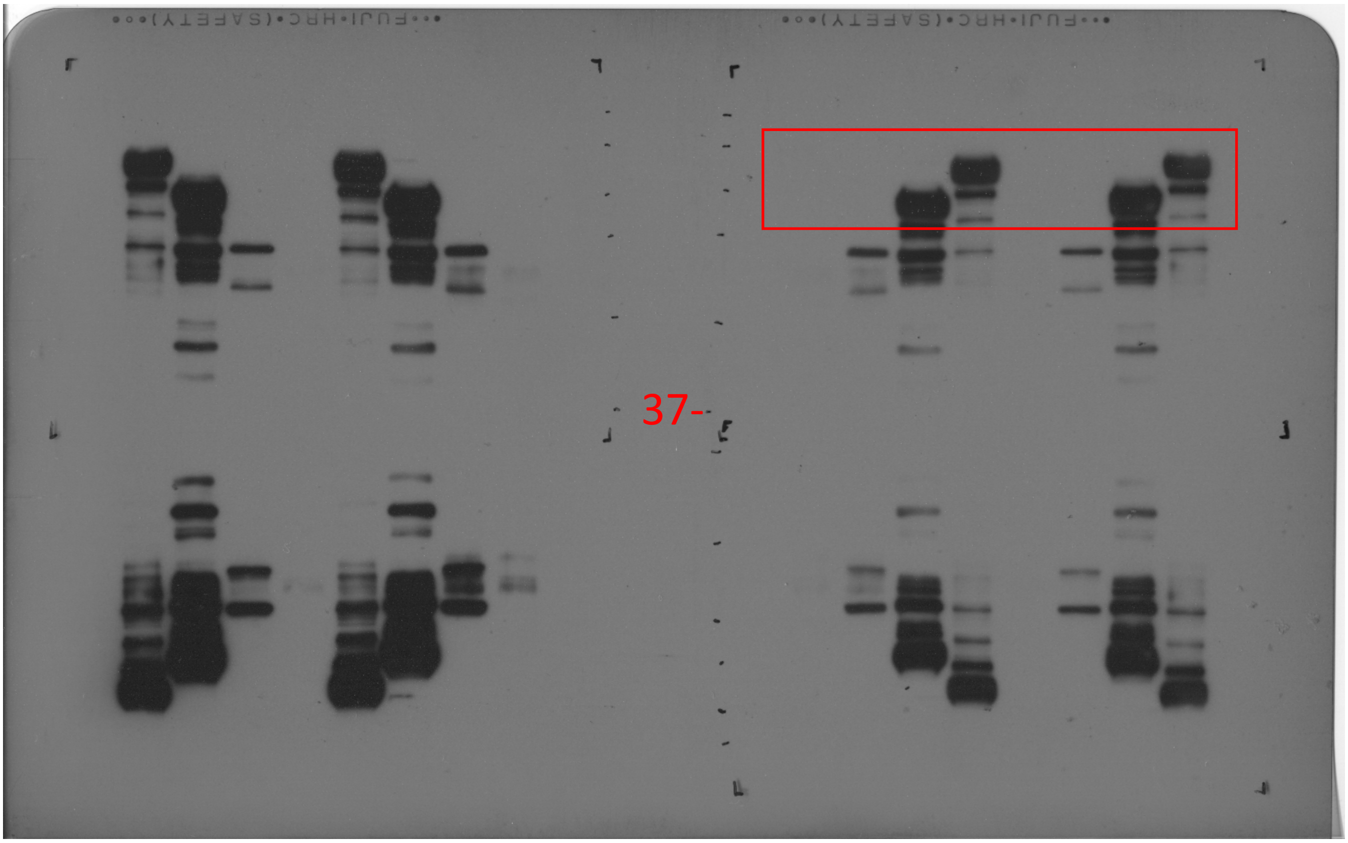

Figure 2A ; DENV_GFP (IP)

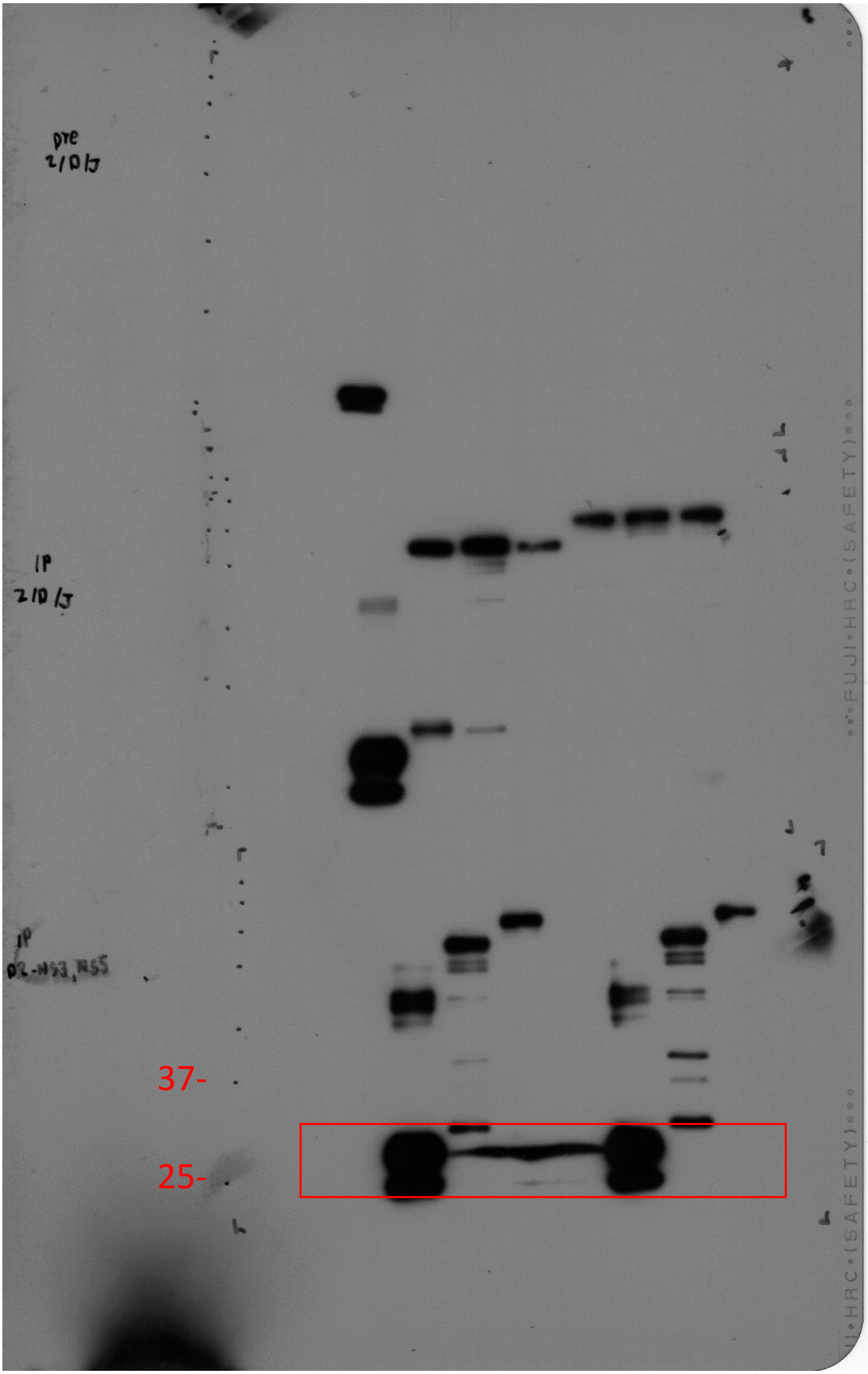

Figure 2A ; DENV_GFP (IP)

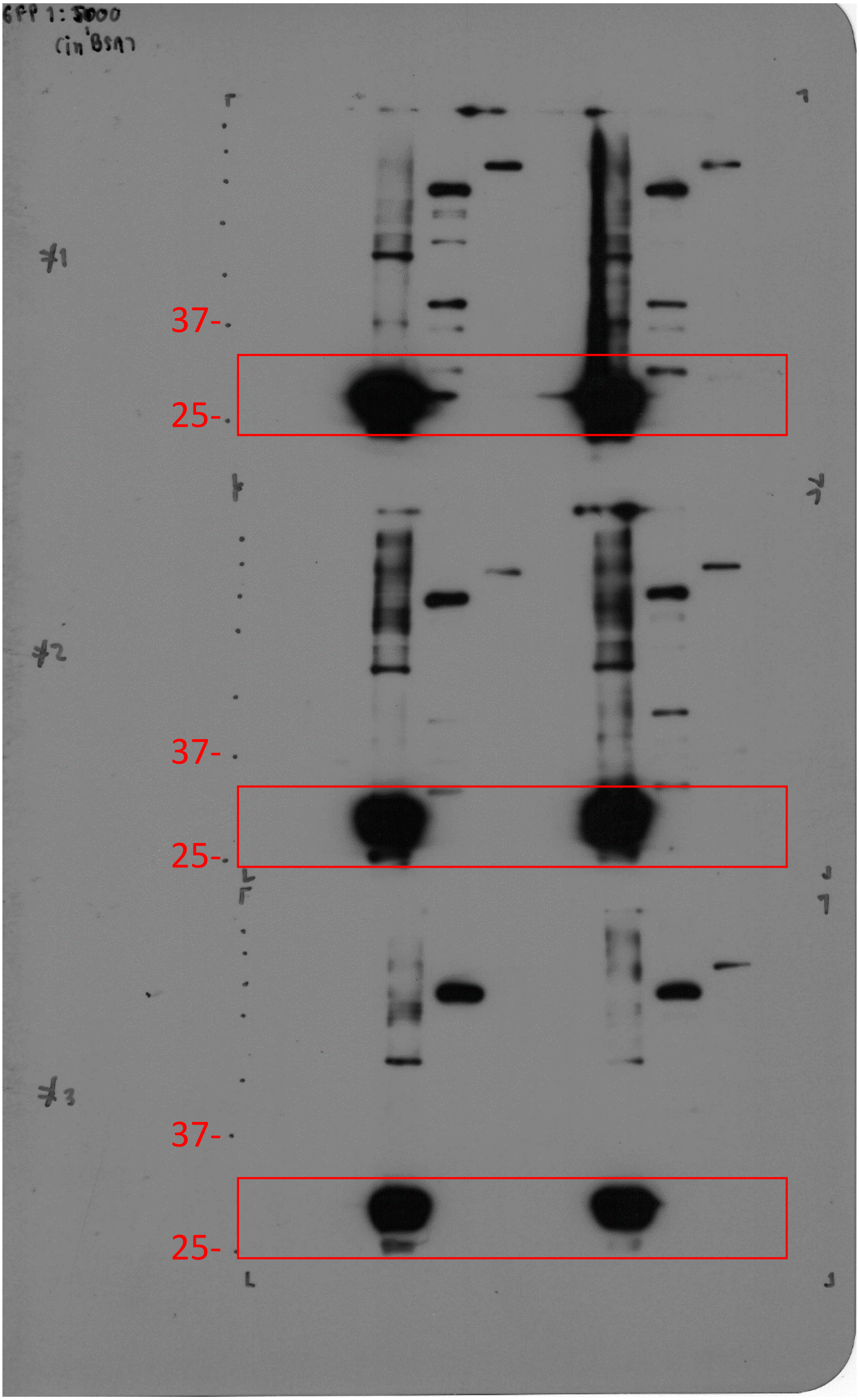

Figure 2A ; DENV_GAPDH (Input)

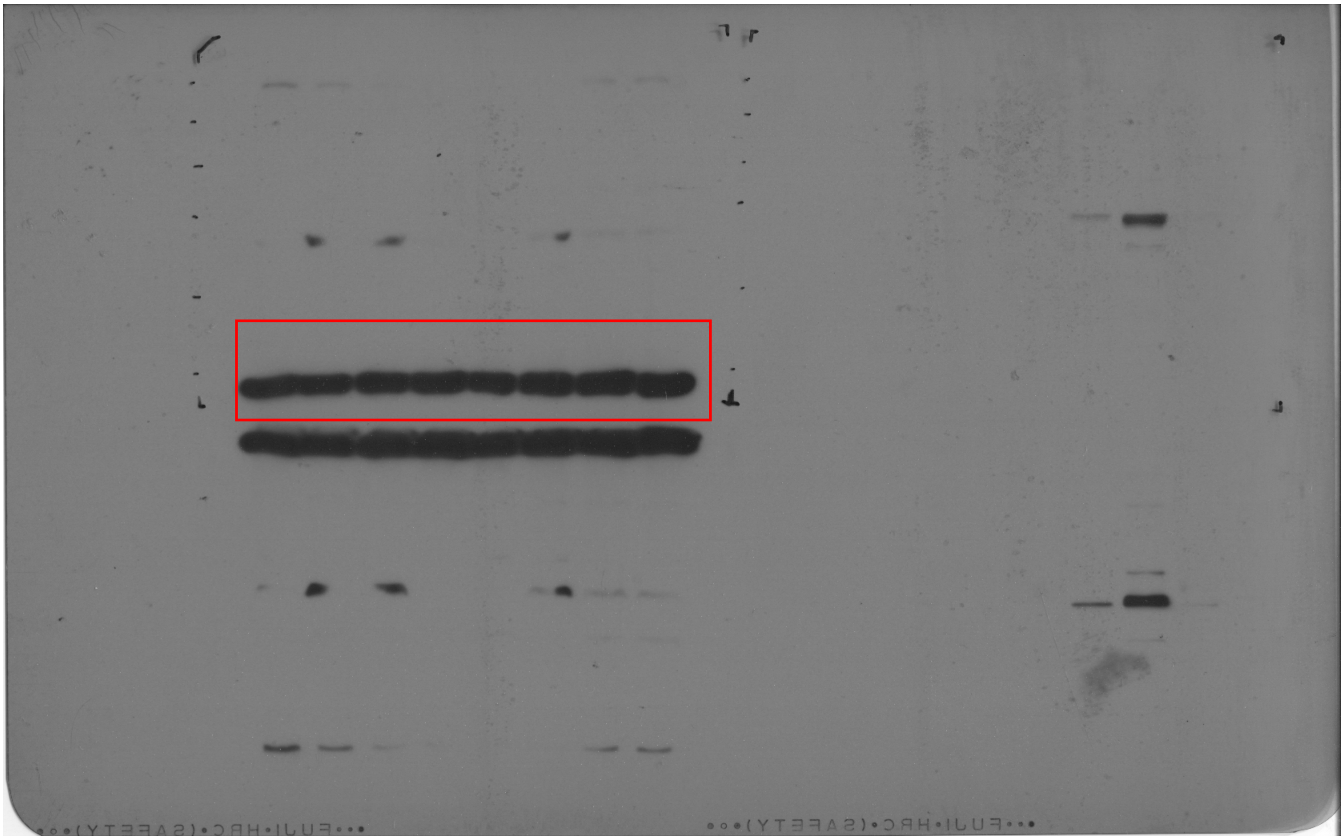

Figure 2A ; DENV_GAPDH (IP)

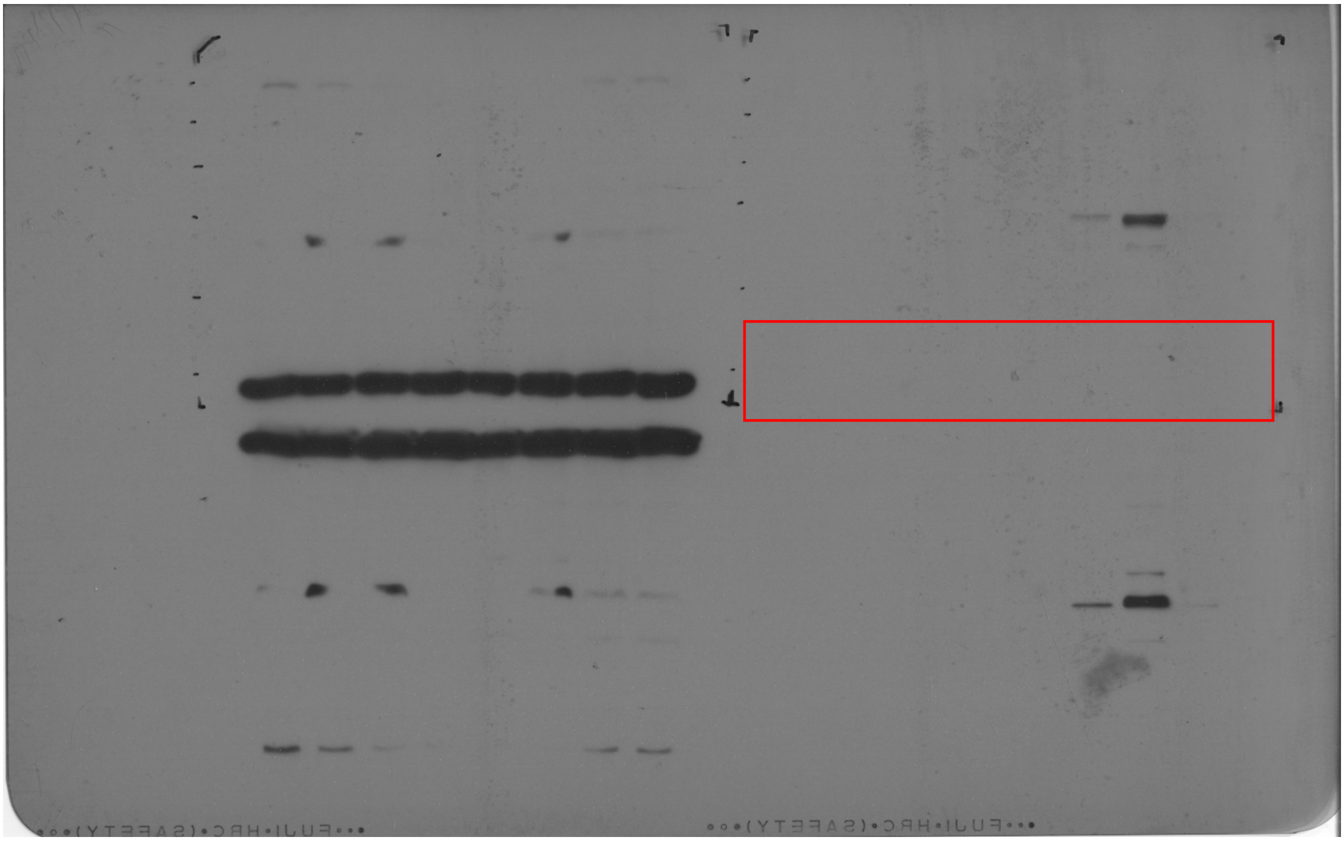

Figure 2A ; DENV_ENV (lysate)

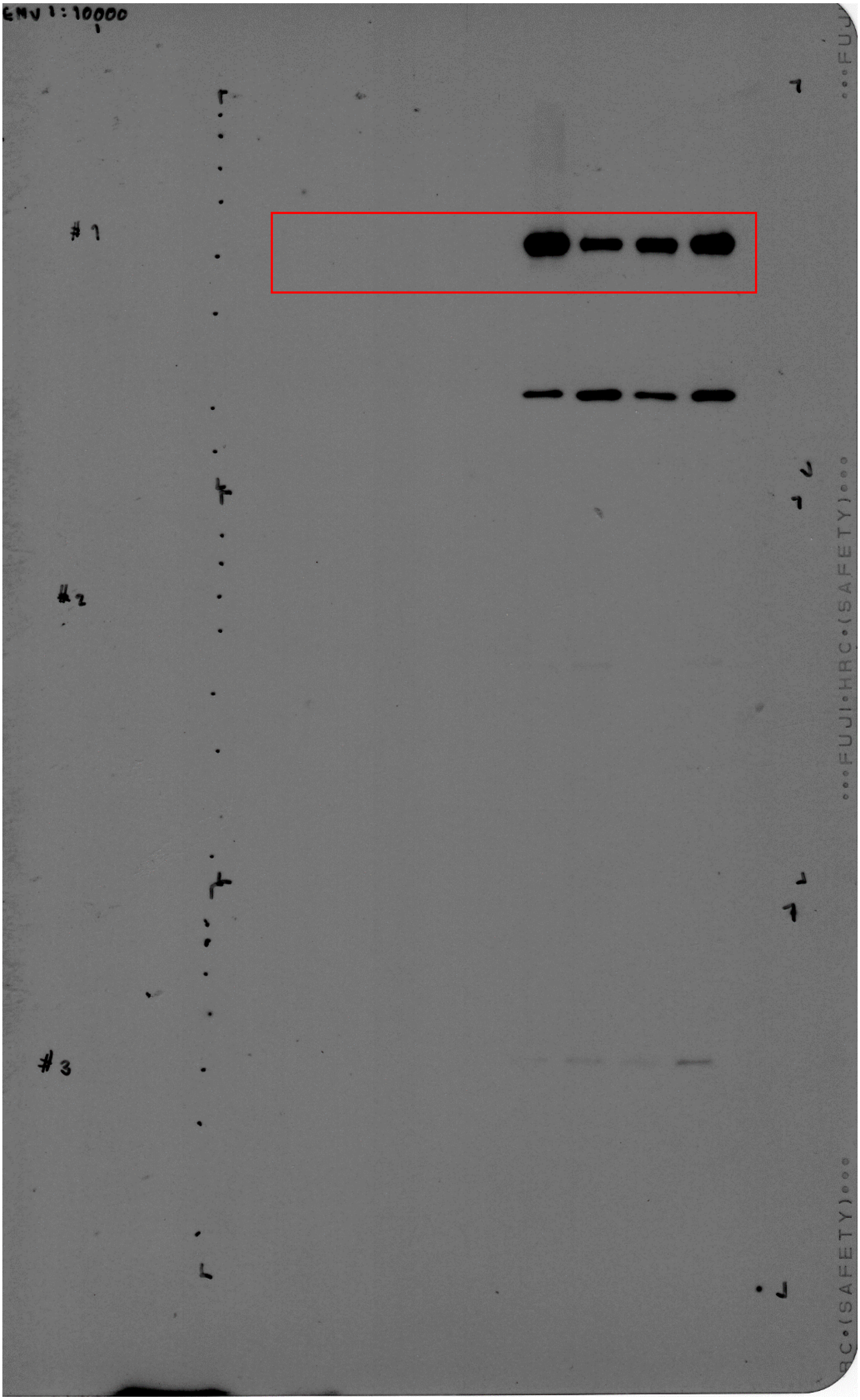

Figure 2A ; DENV_FASN (Input) #Replicate 2

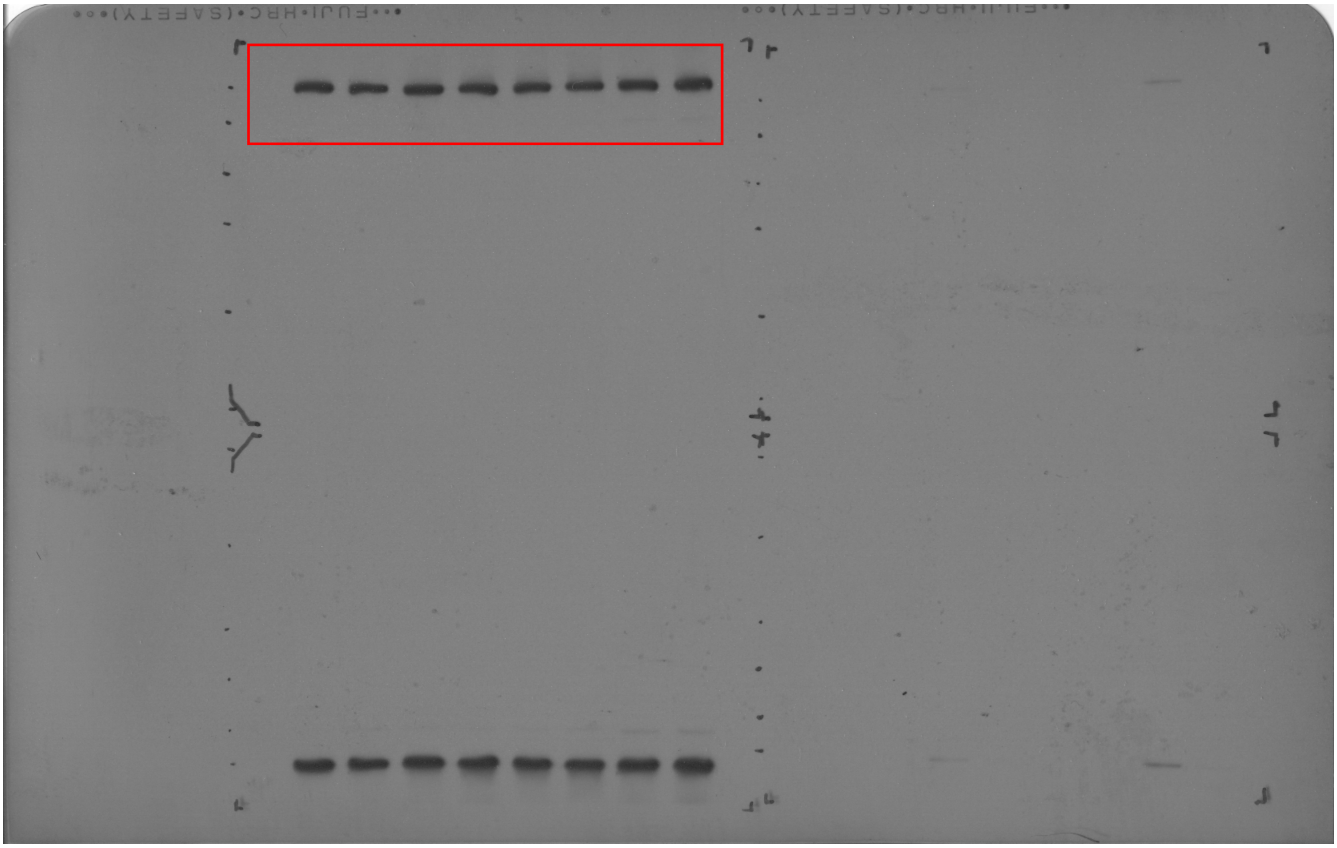

Figure 2A ; DENV_FASN (IP) #Replicate 2

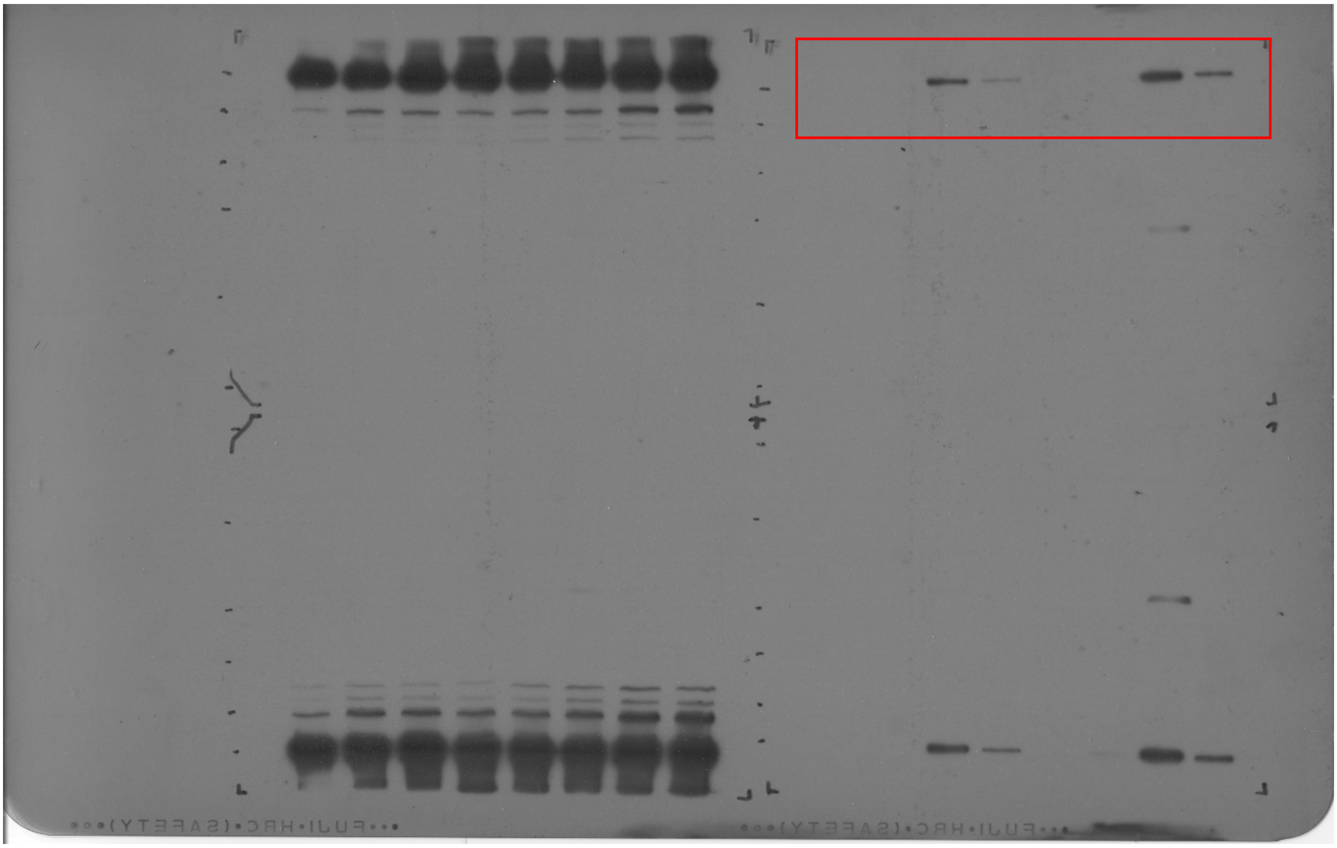

Figure 2A ; DENV_GFP (Input) #Replicate 2

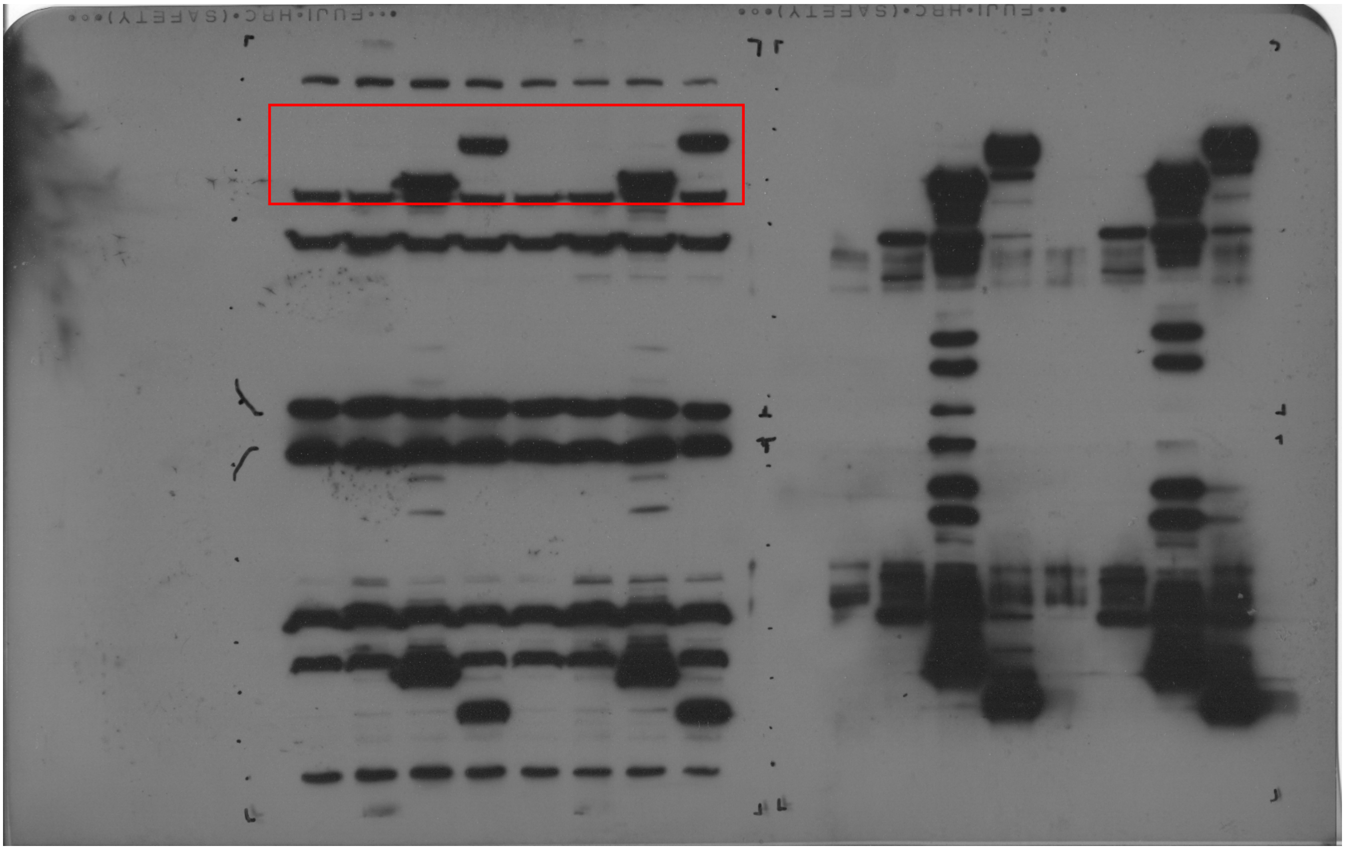

Figure 2A ; DENV_GFP (IP) #Replicate 2

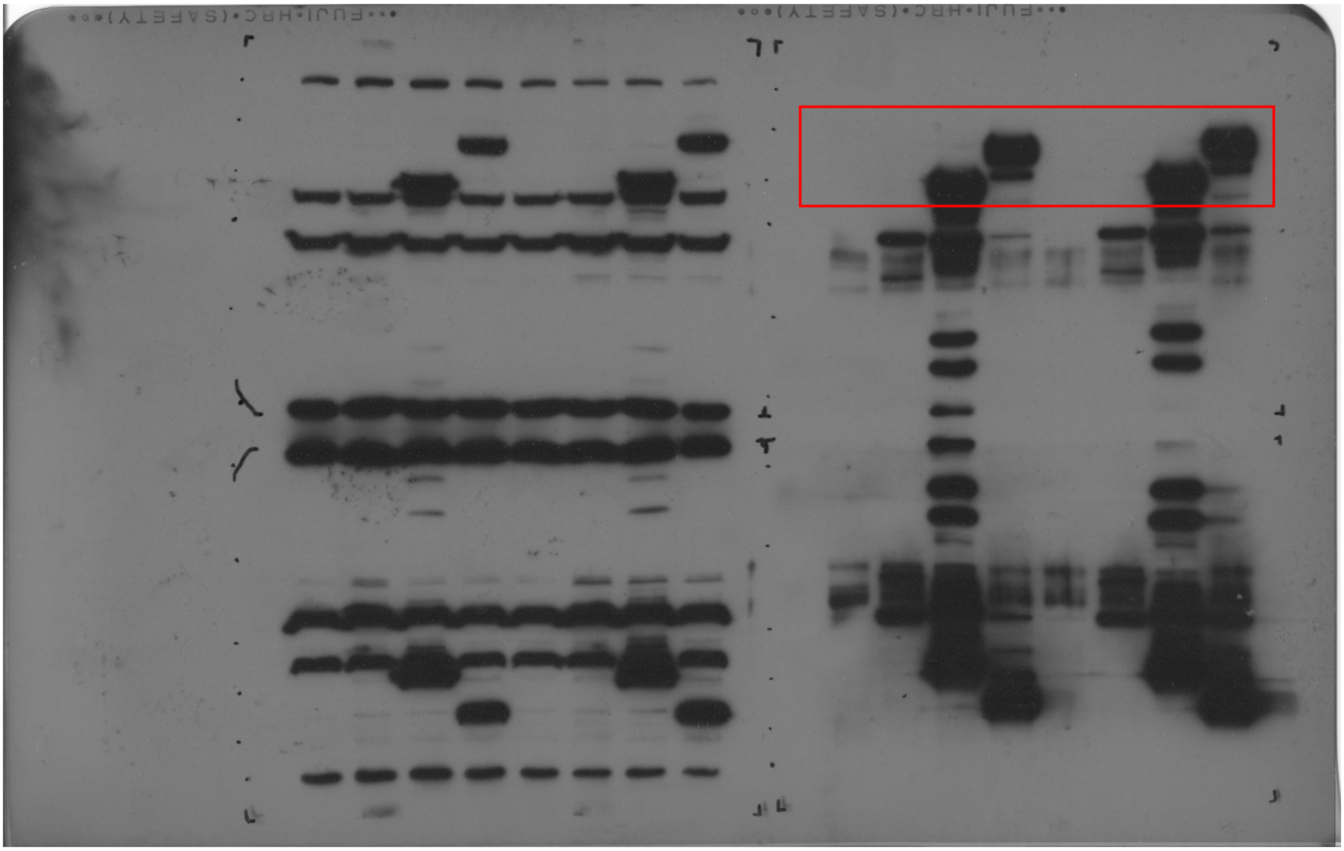

Figure 2A ; DENV_GAPDH (Input) #Replicate 2

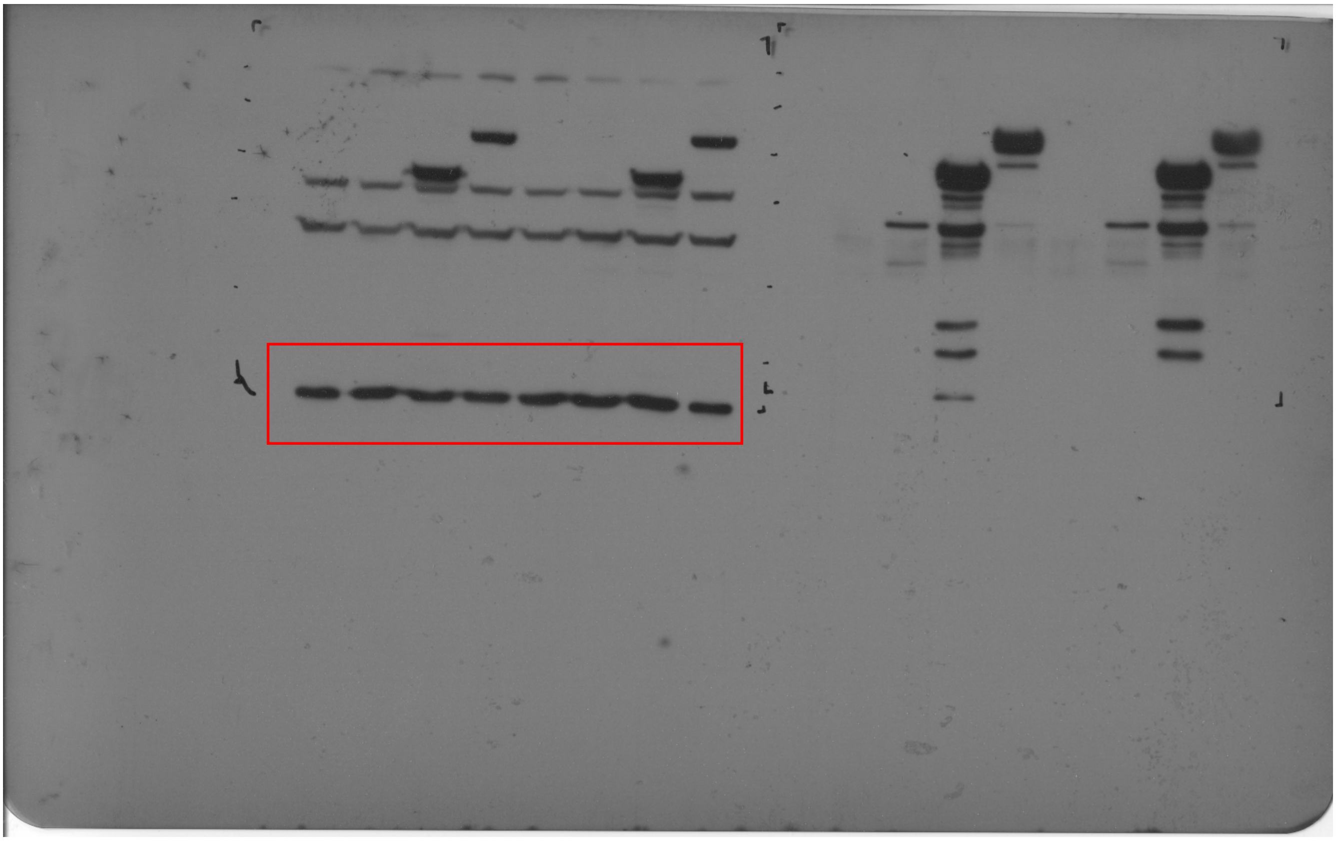

Figure 2A ; DENV_GAPDH (IP) #Replicate 2

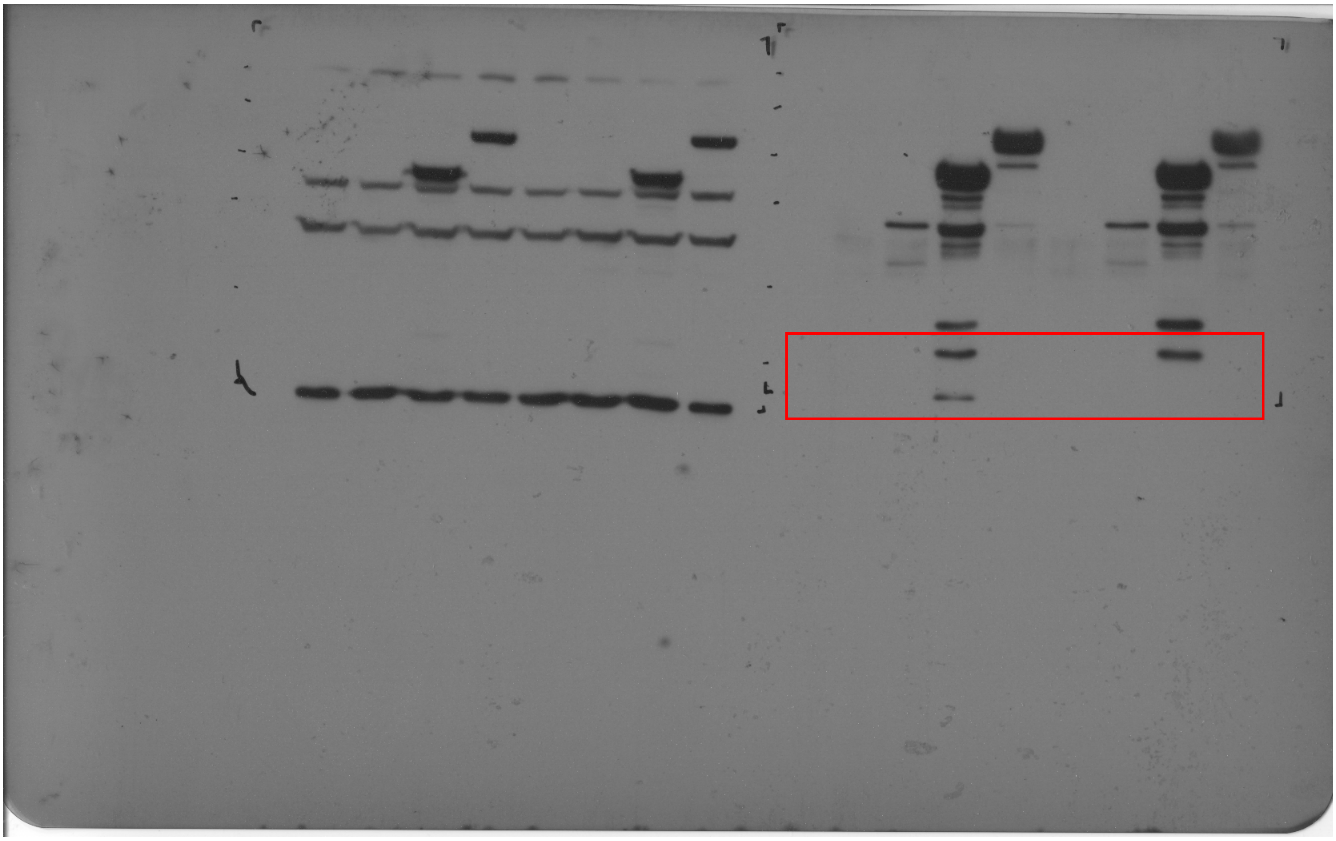

Figure 2A ; DENV_FASN (Input) #Replicate 3

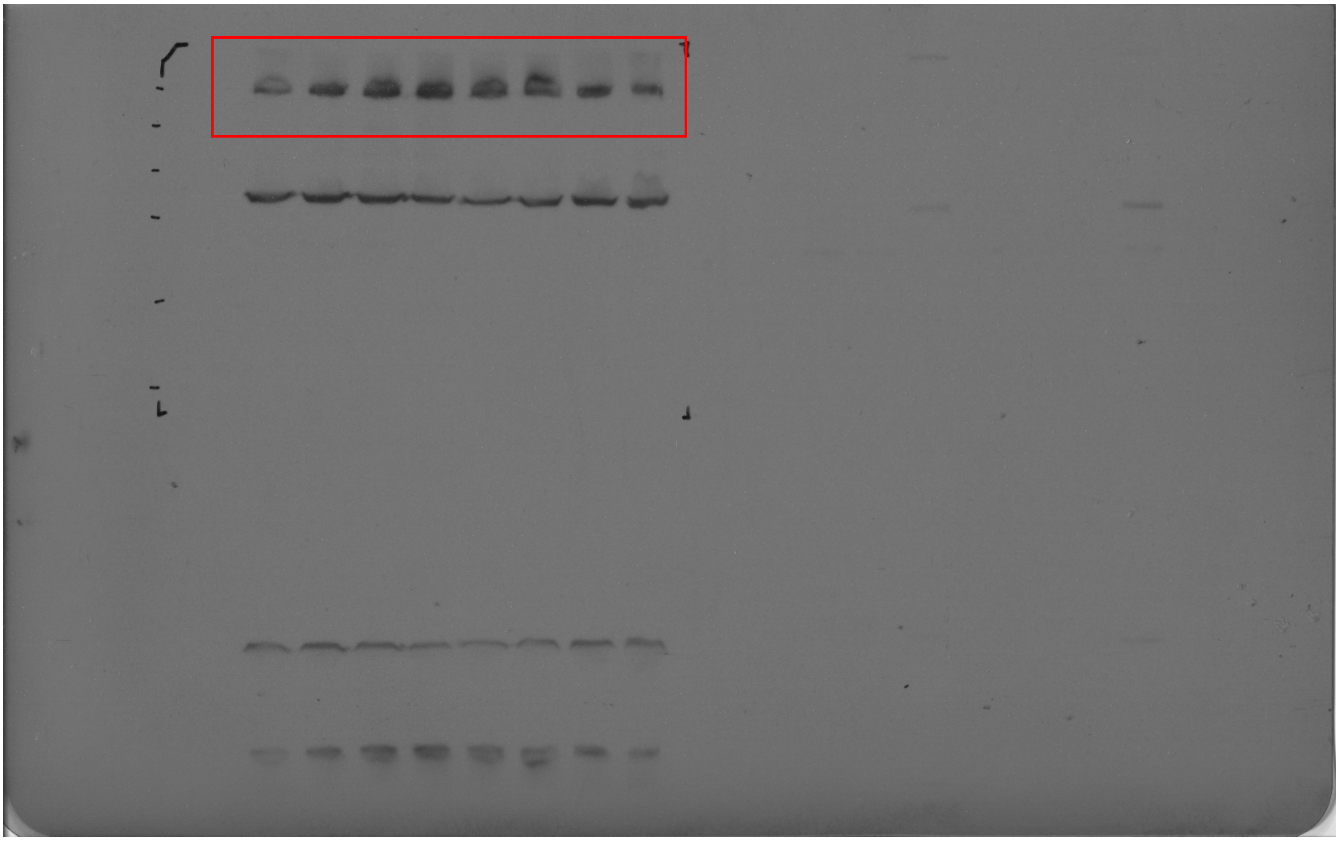

Figure 2A ; DENV_FASN (IP) #Replicate 3

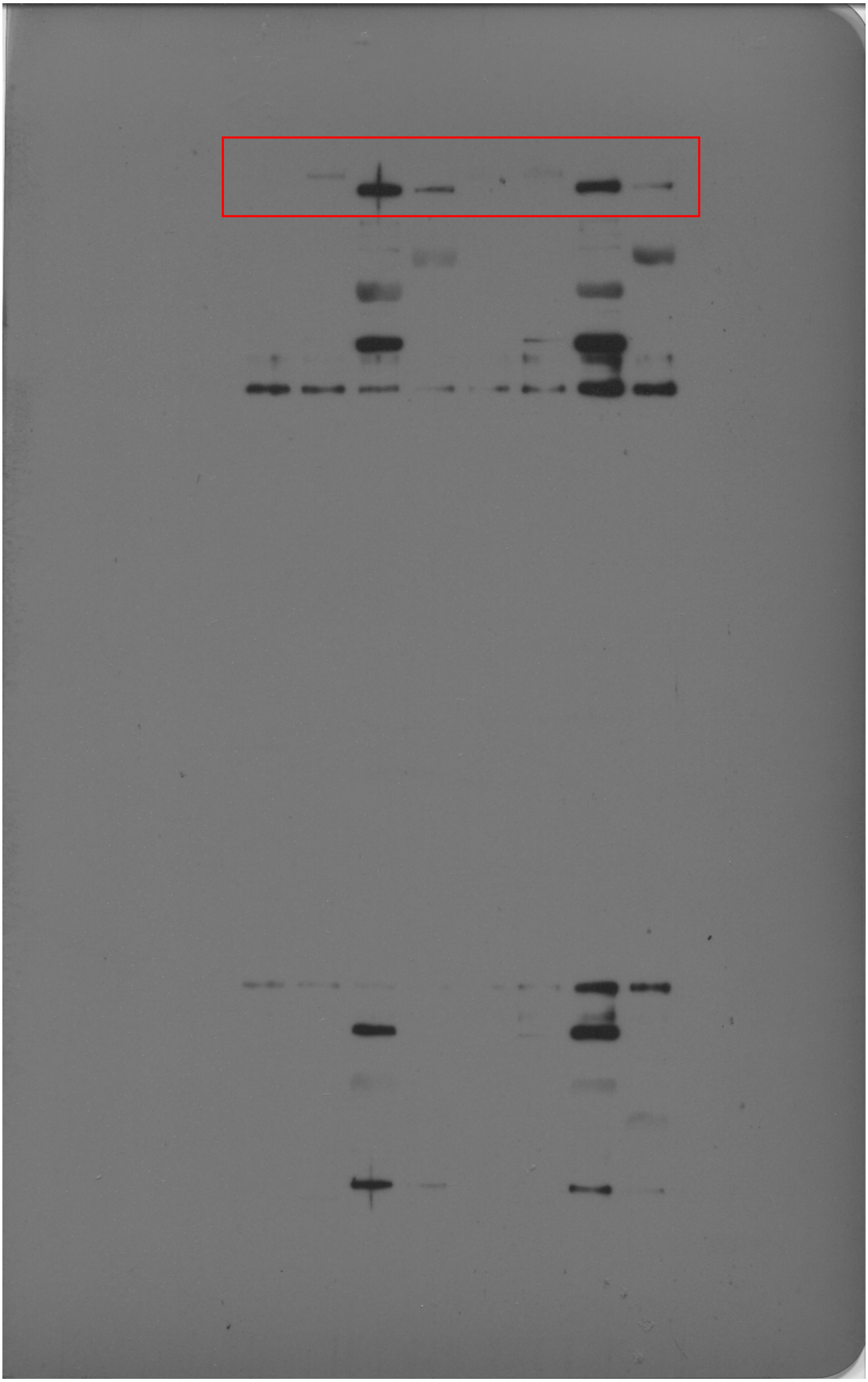

Figure 2A ; DENV_GFP (Input) #Replicate 3

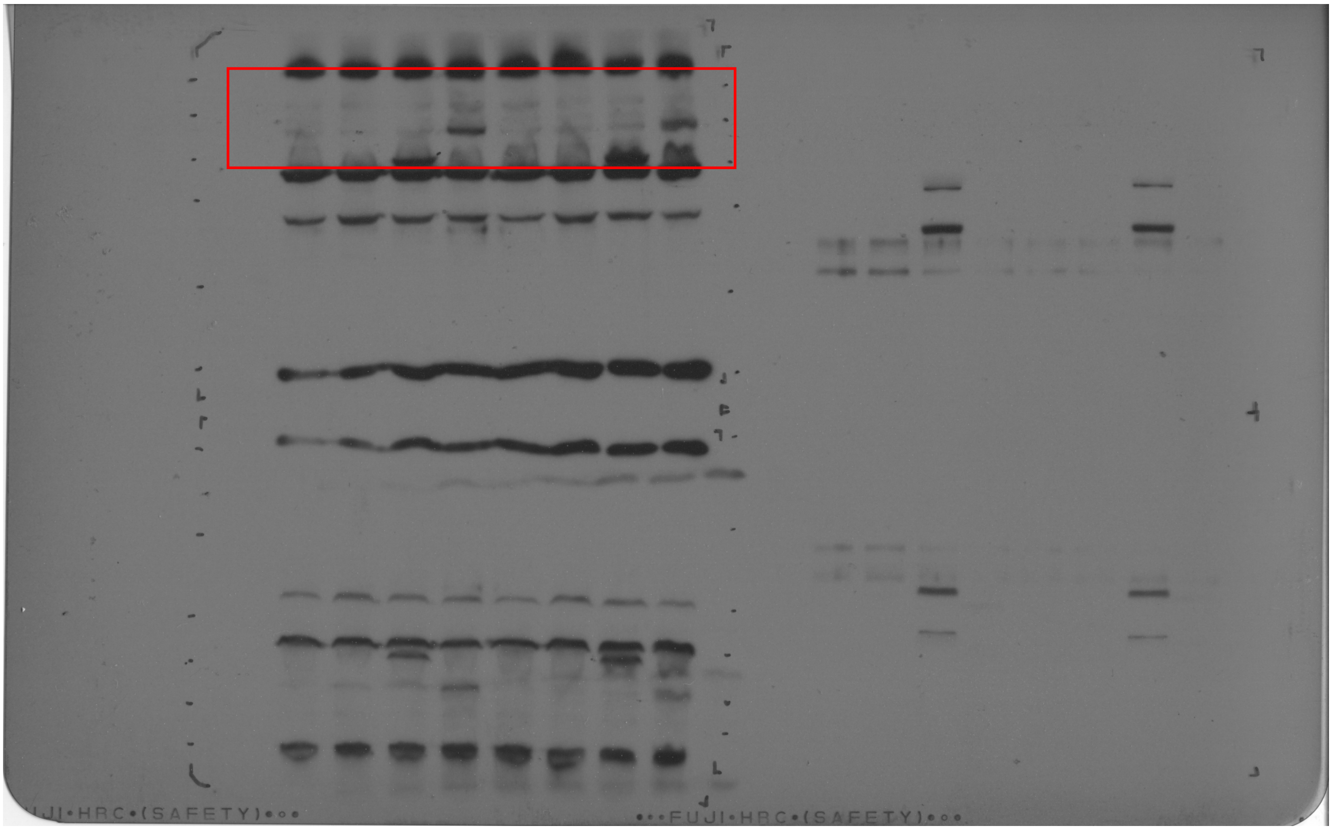

Figure 2A ; DENV_GFP (IP) #Replicate 3

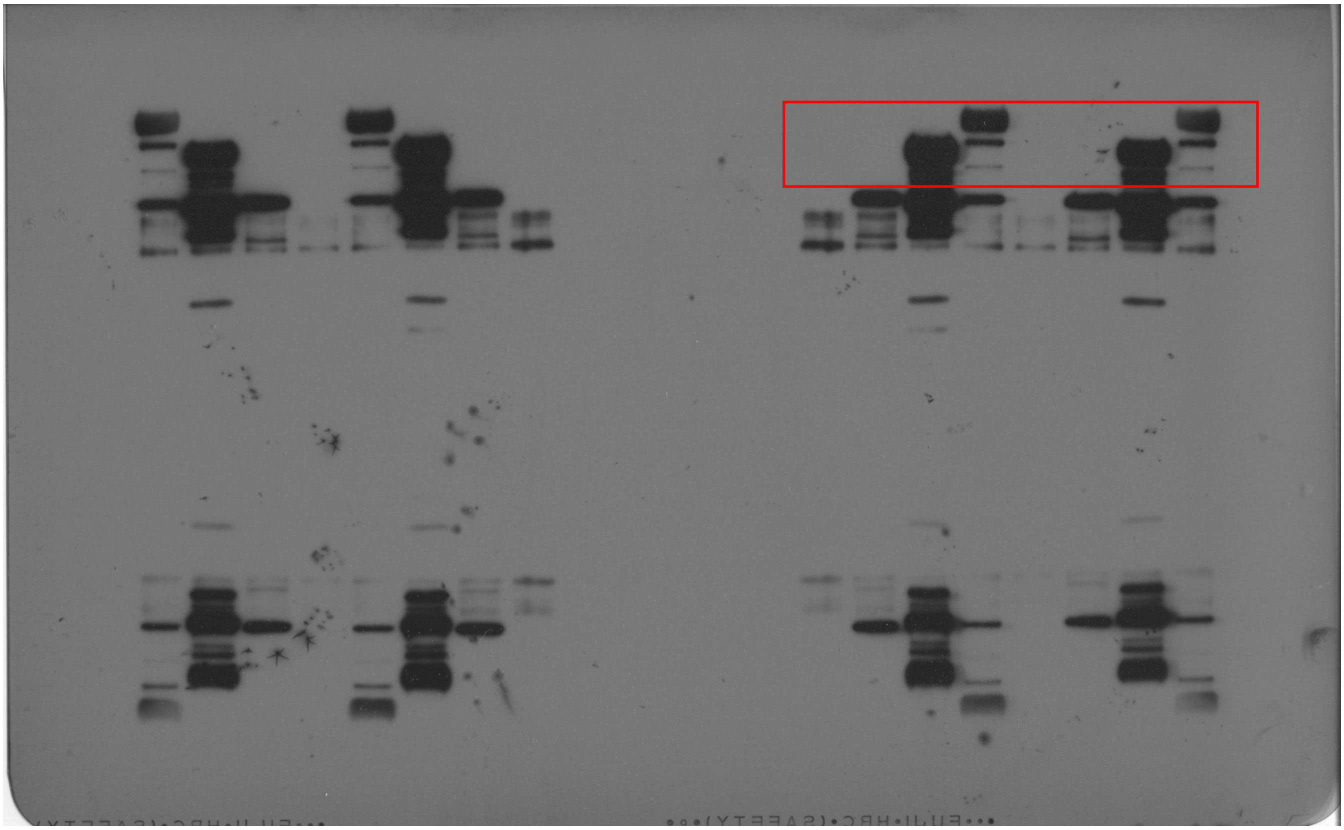

Figure 2A ; DENV_GAPDH (Input) #Replicate 3

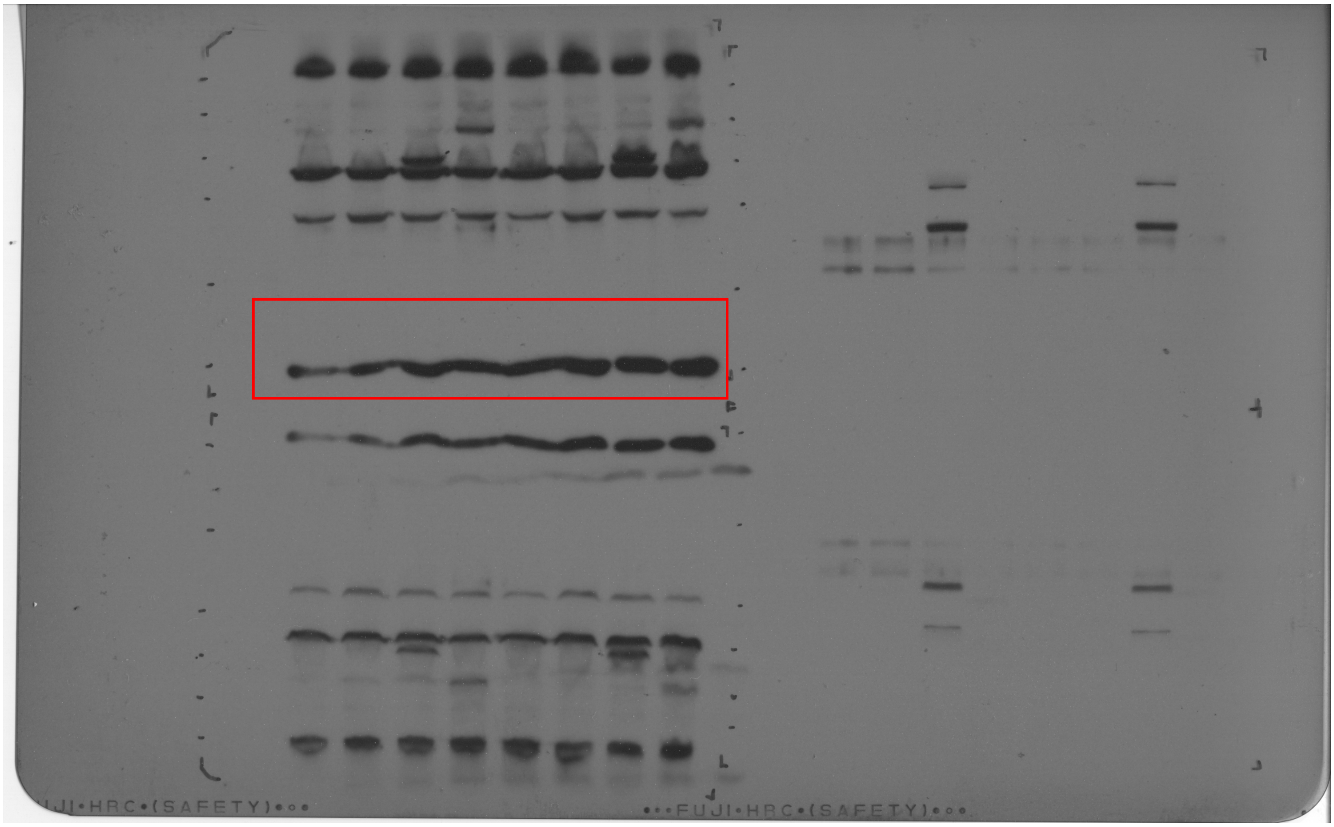

Figure 2A ; DENV_GAPDH (IP) #Replicate 3

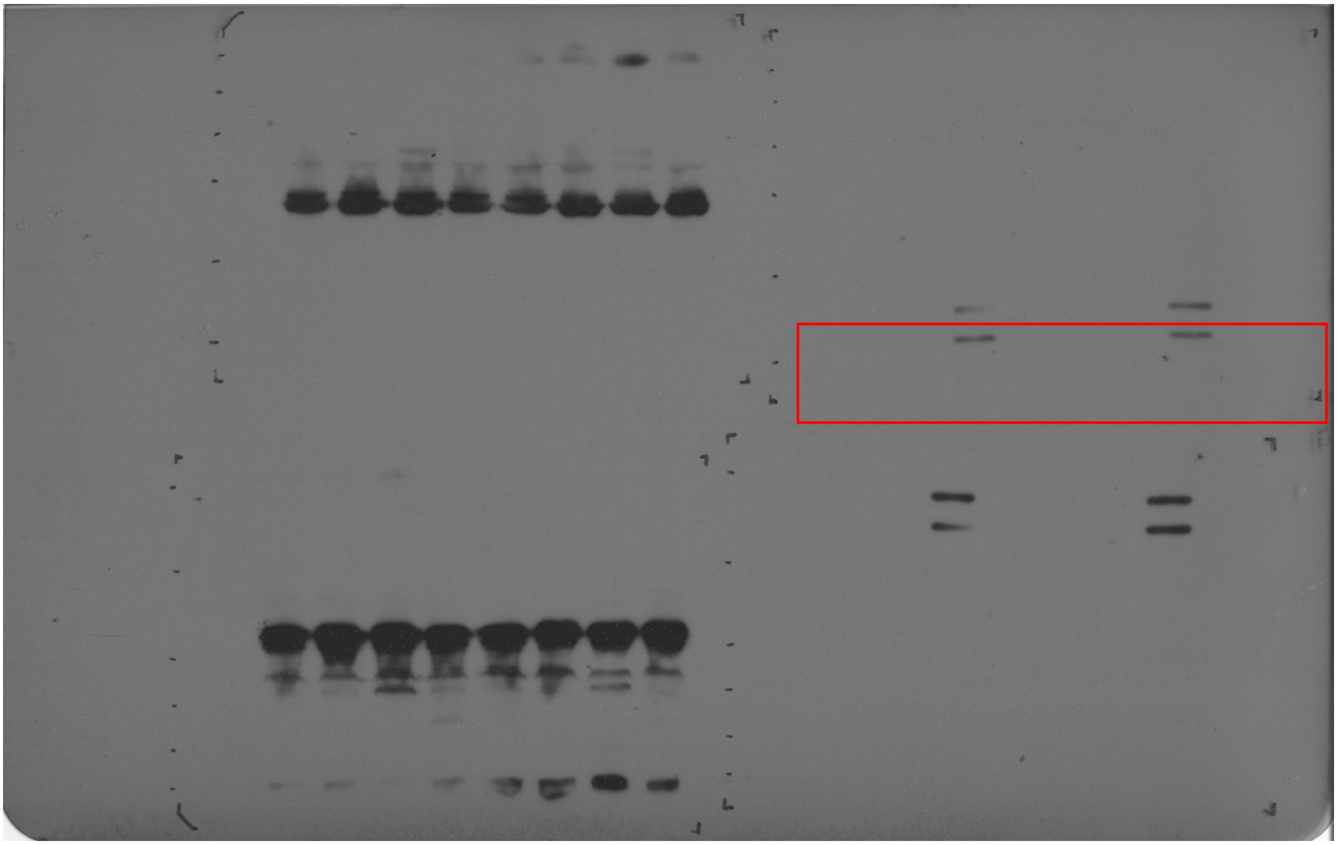

Figure 2A ; DENV_ENV (lysate) #Replicate2, 3

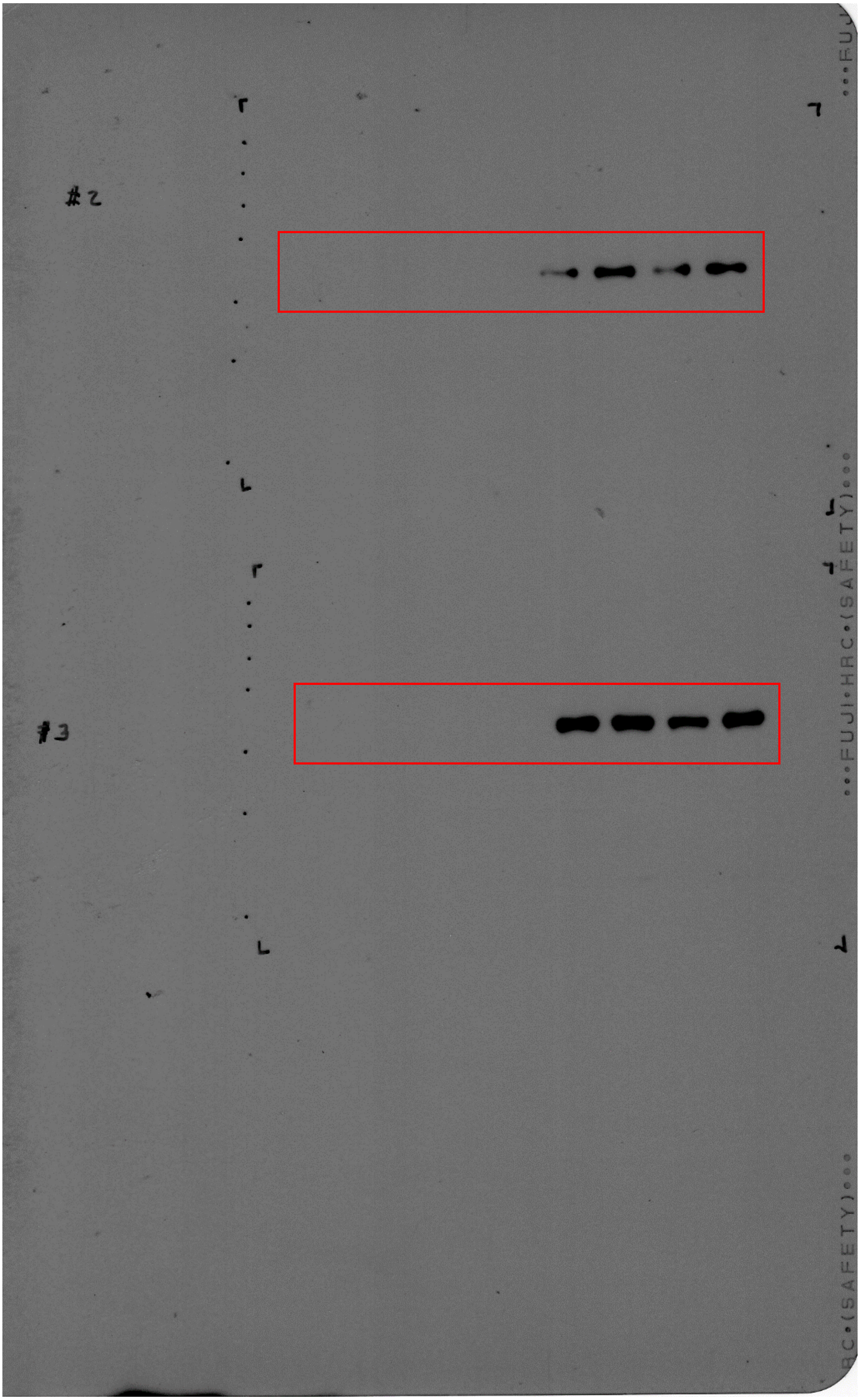

Figure 2C ; ZIKV_FASN (IP) #1, 2, 3

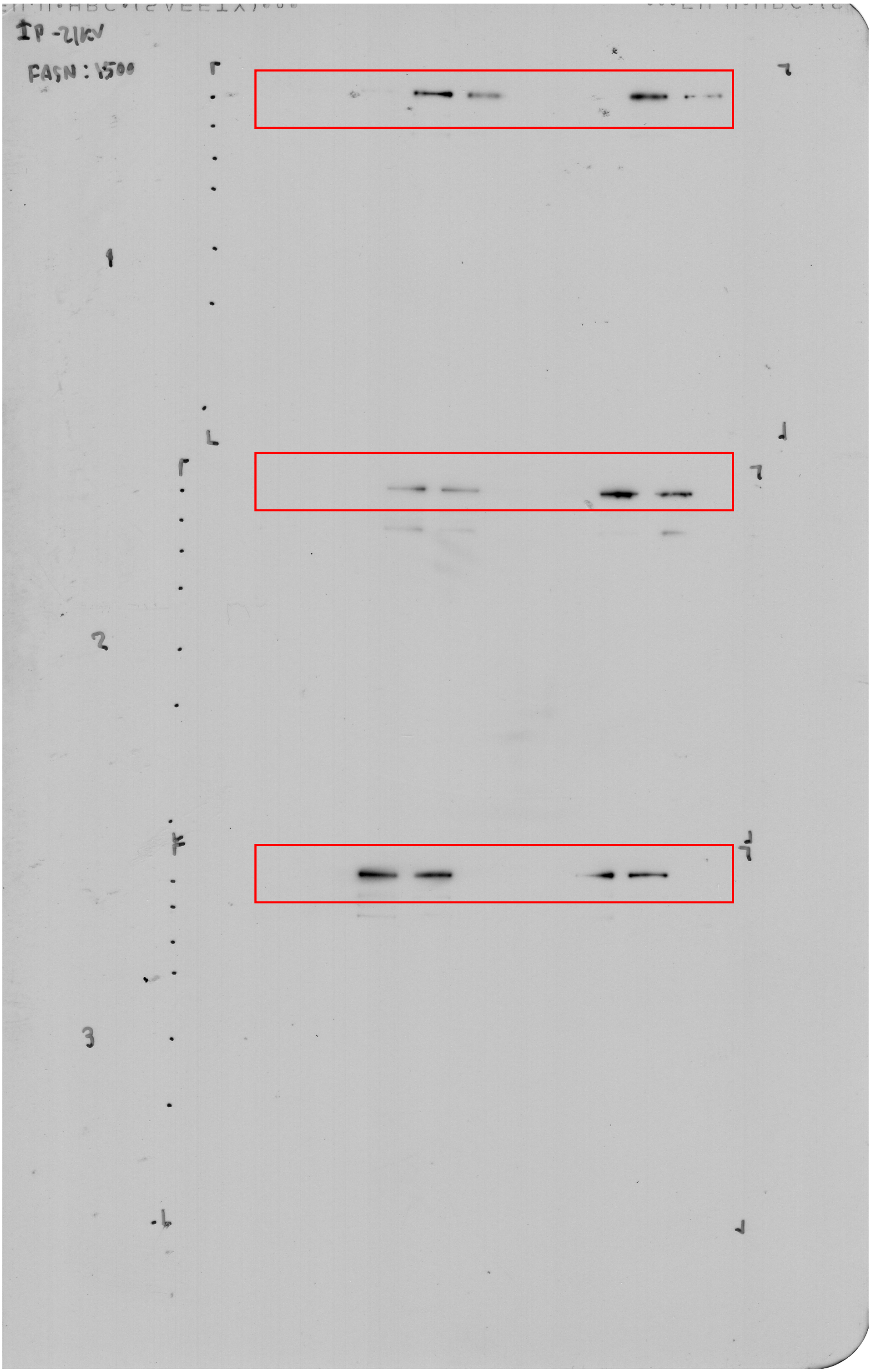

Figure 2C ; ZIKV_FASN (lysate)

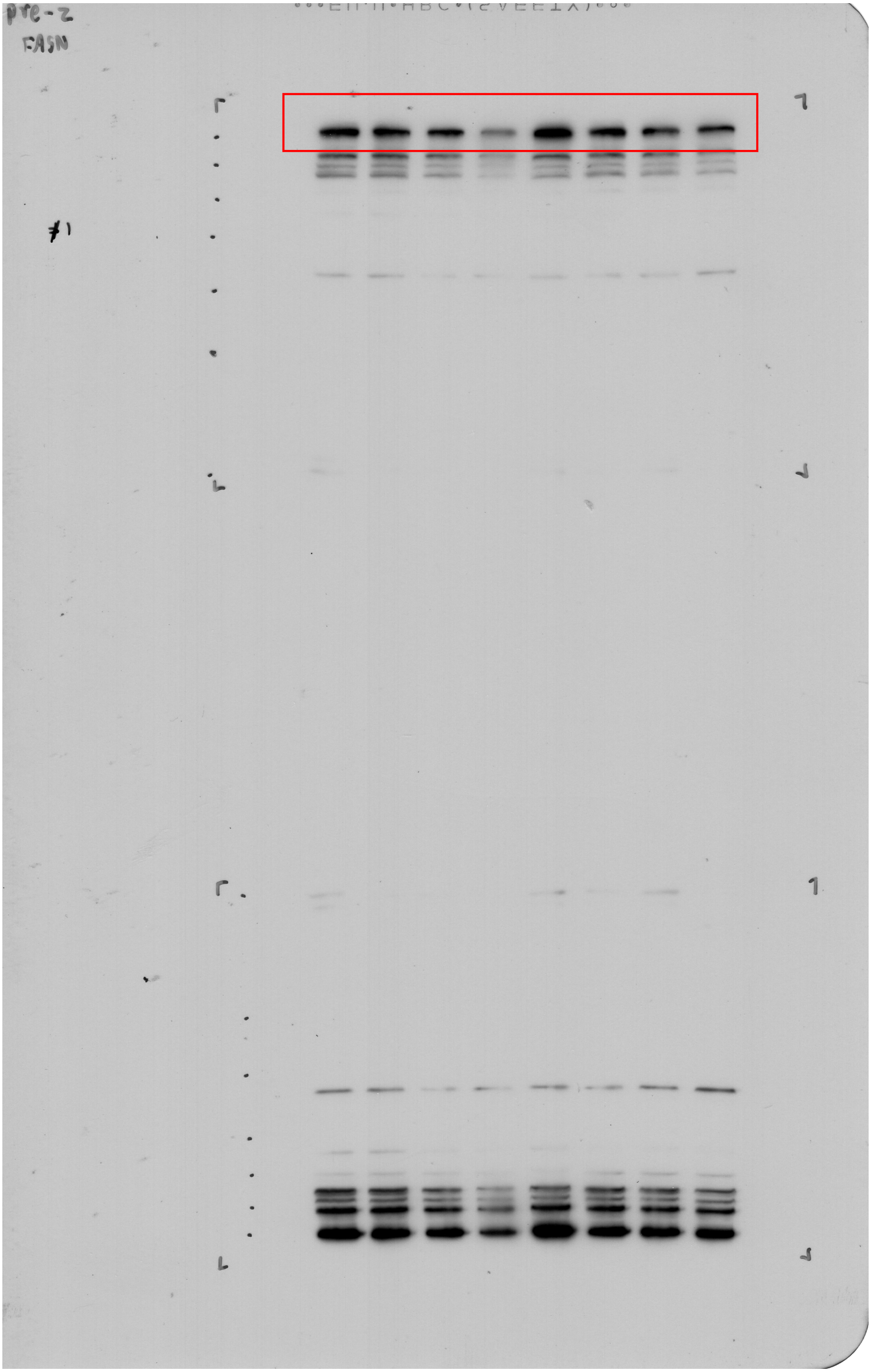

Figure 2C ; ZIKV_FASN (lysate) #2, 3

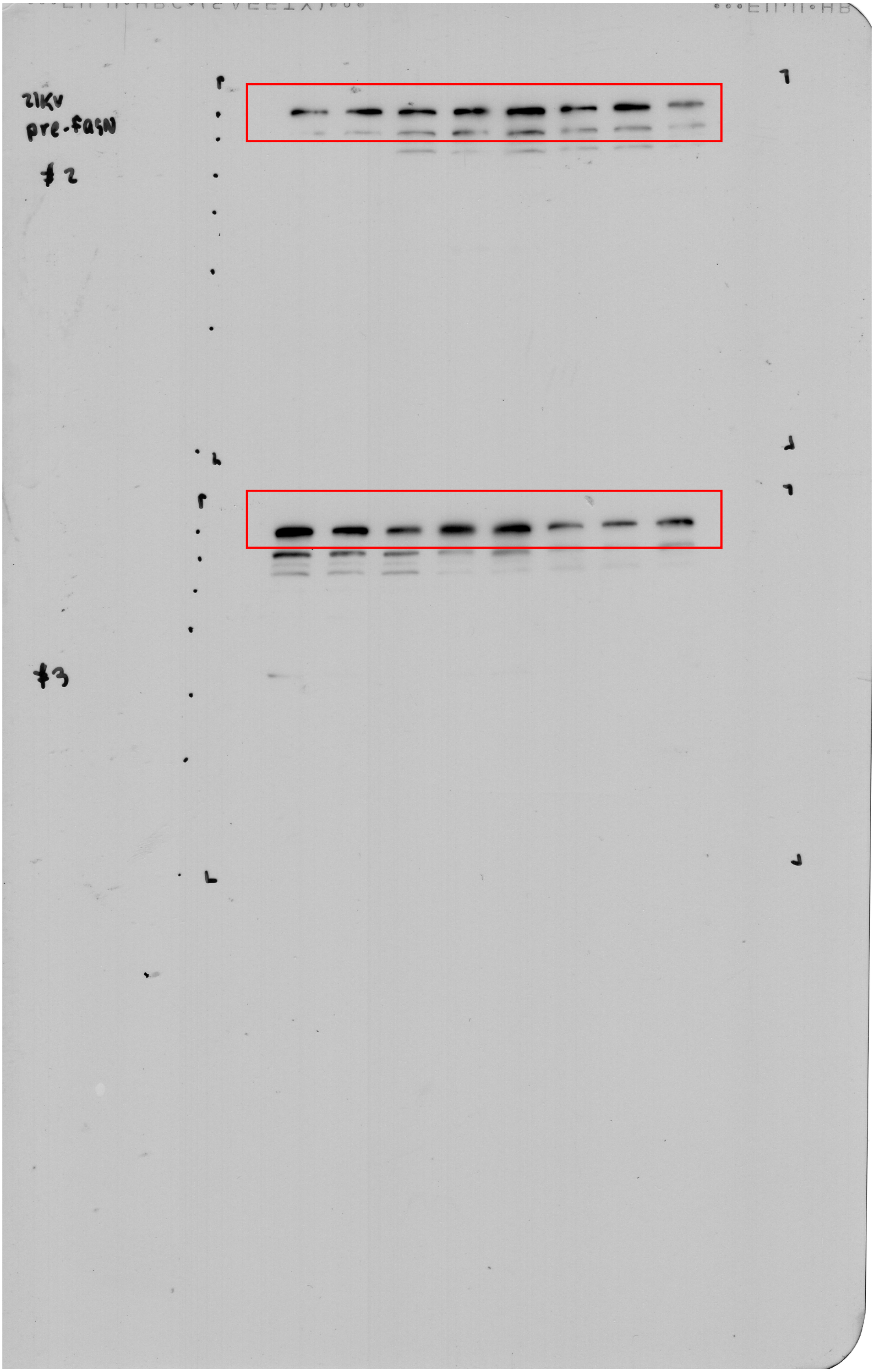

Figure 2C ; ZIKV_GFP (IP) #1,2, 3

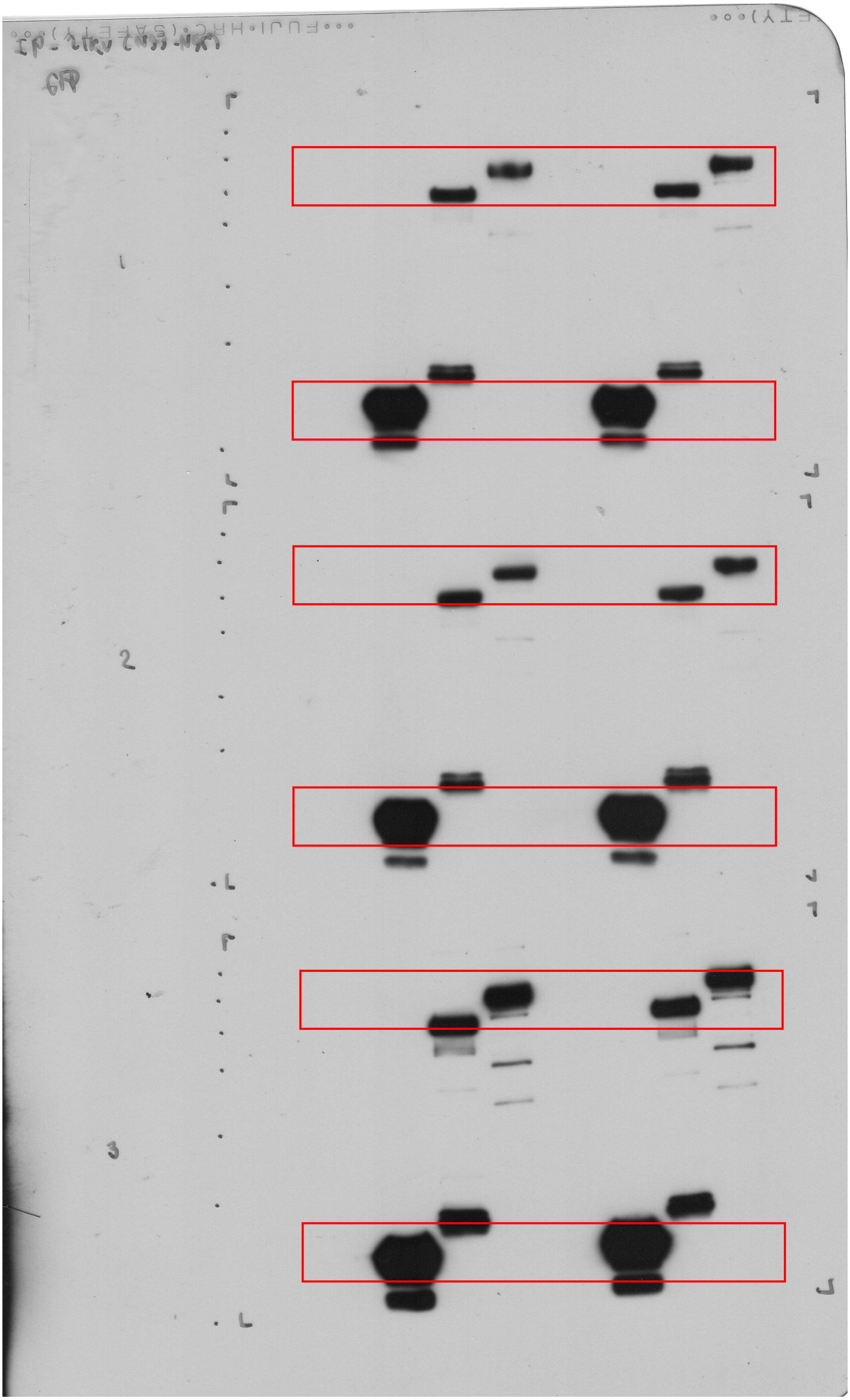

Figure 2C ; ZIKV_GFP (lysate) #1,2, 3

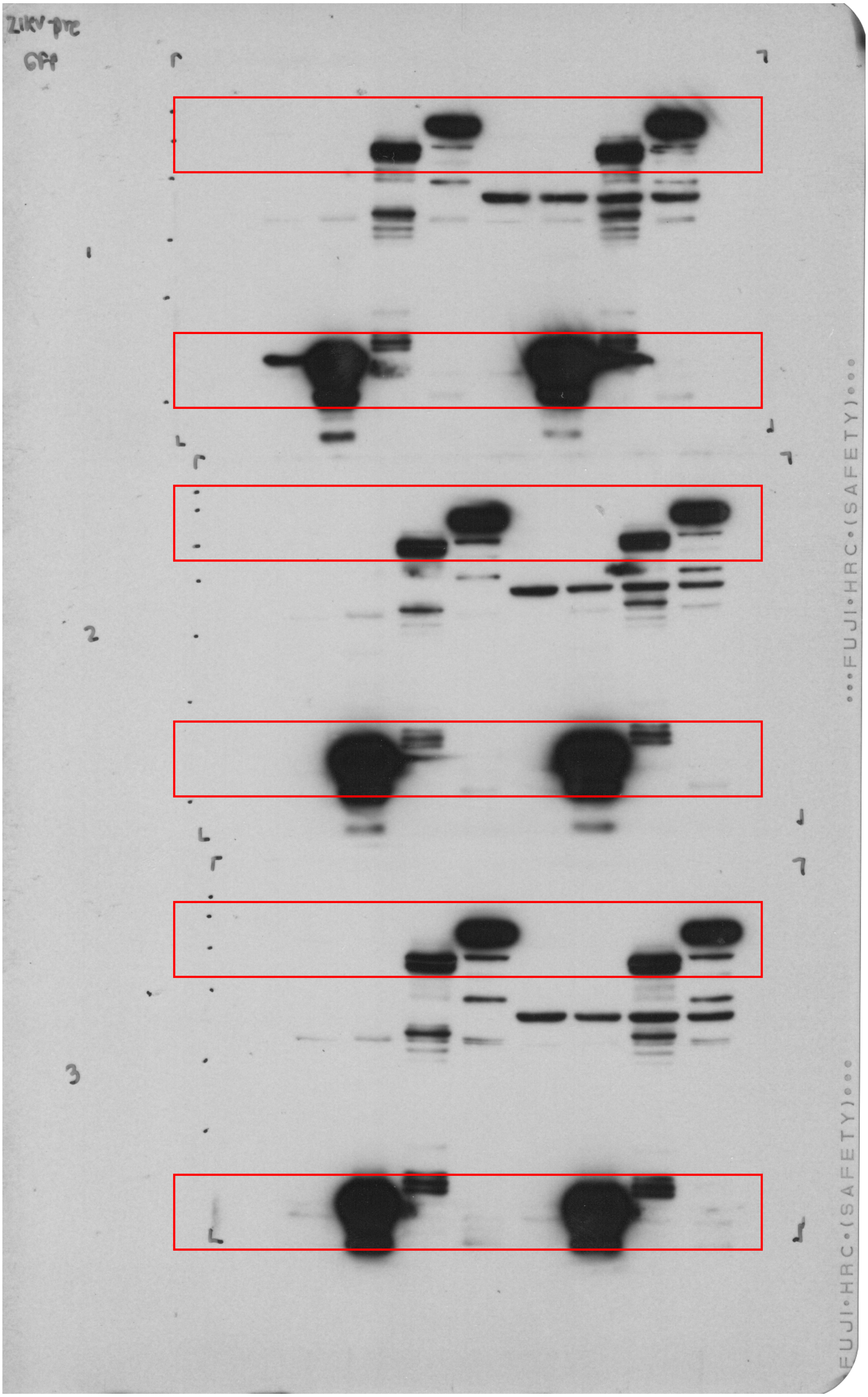

Figure 2C ; ZIKV_ENV (lysate) #1,2, 3

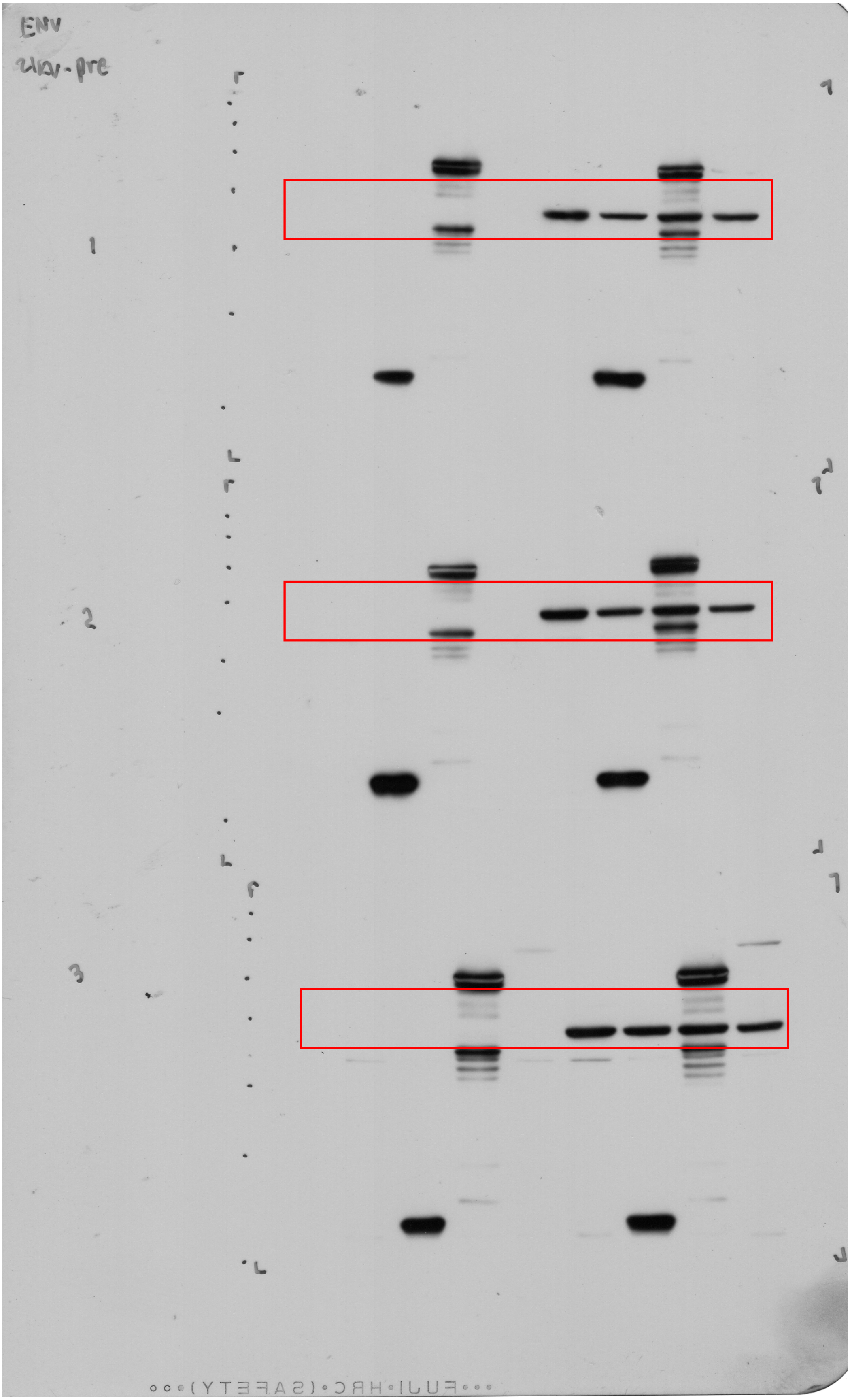

Figure 2C ; ZIKV_GAPDH (lysate) #1

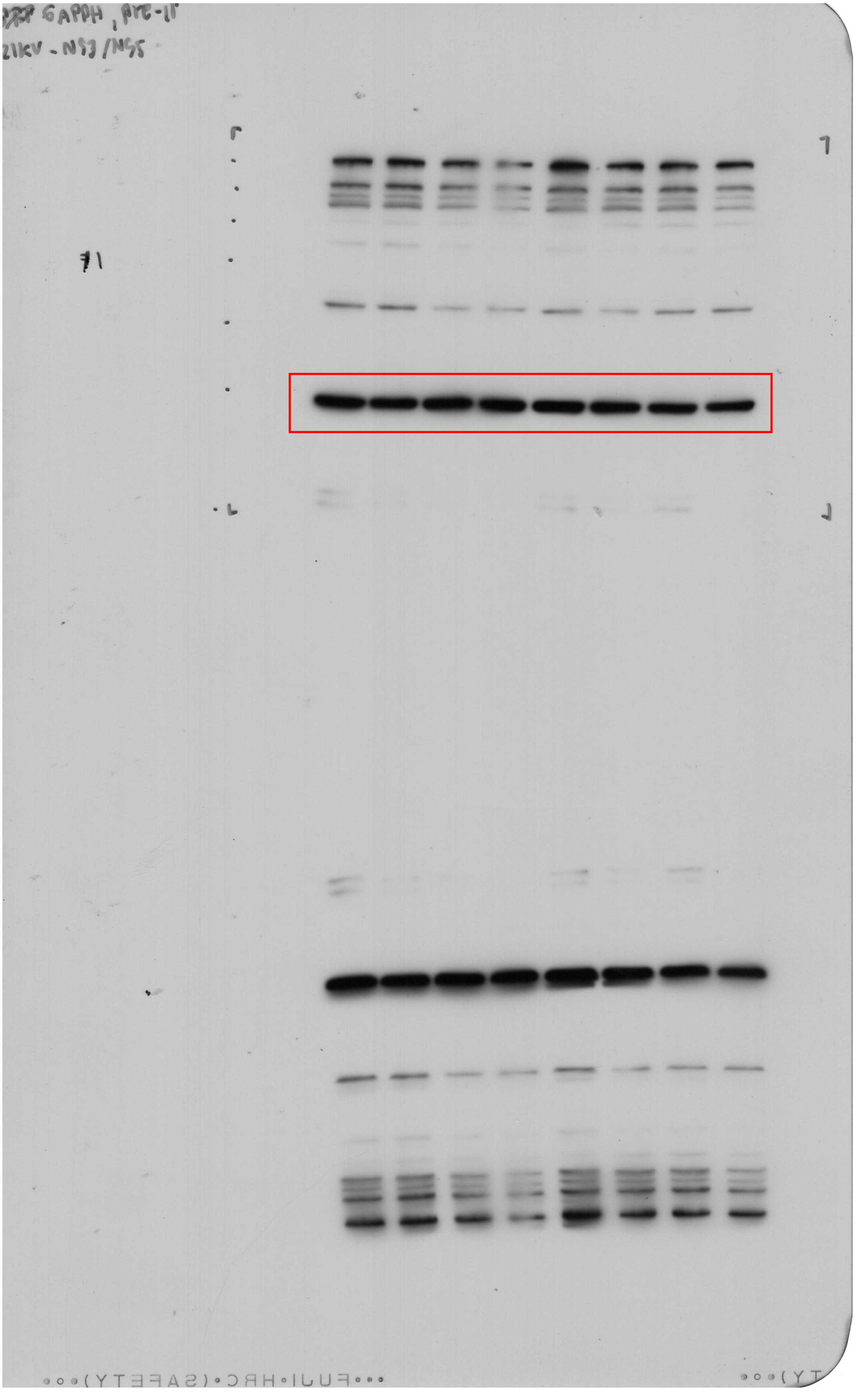

Figure 2C ; ZIKV_GAPDH (lysate) #2, 3

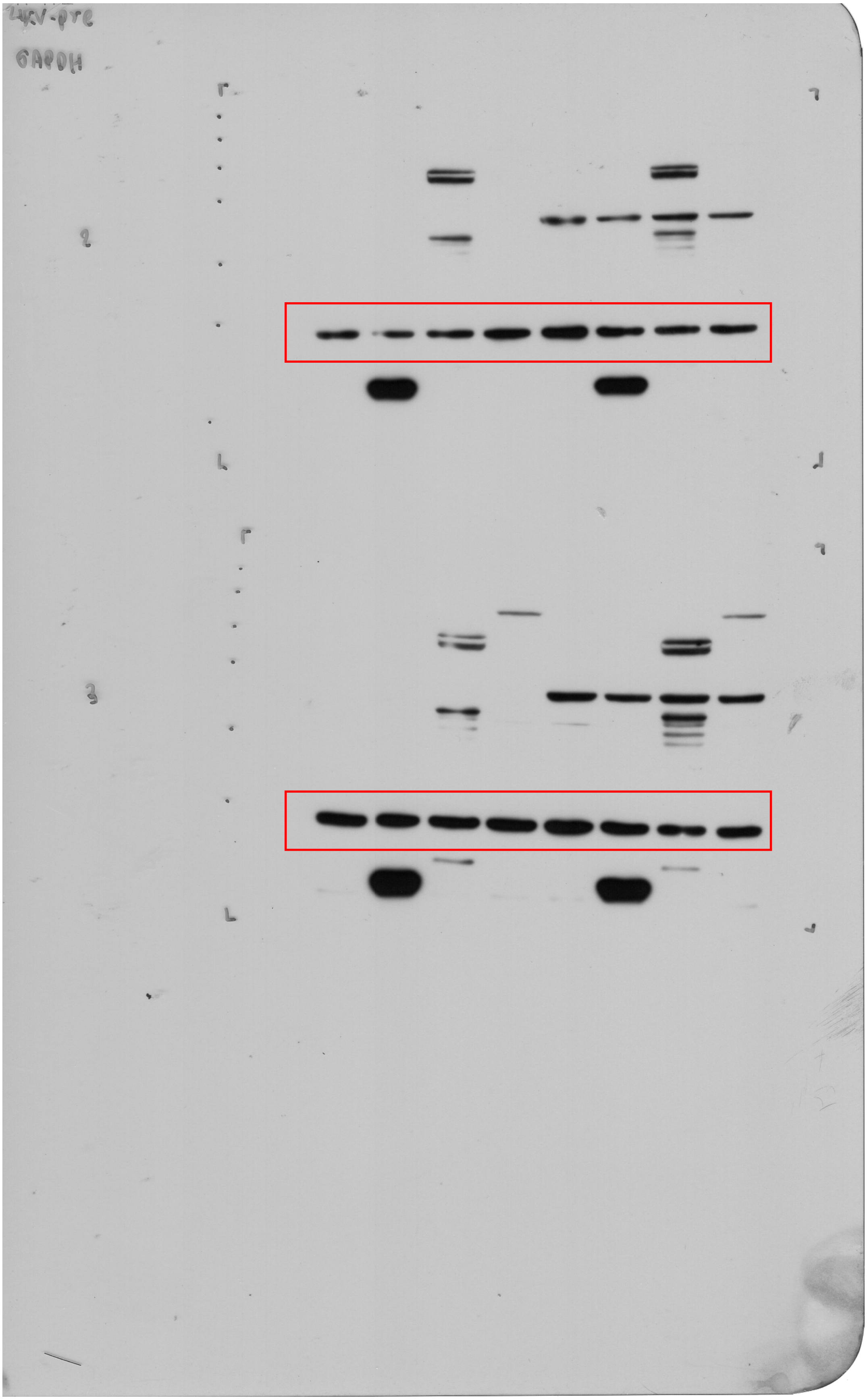

Figure 2E ; JEV_FASN (IP) #1, 2, 3

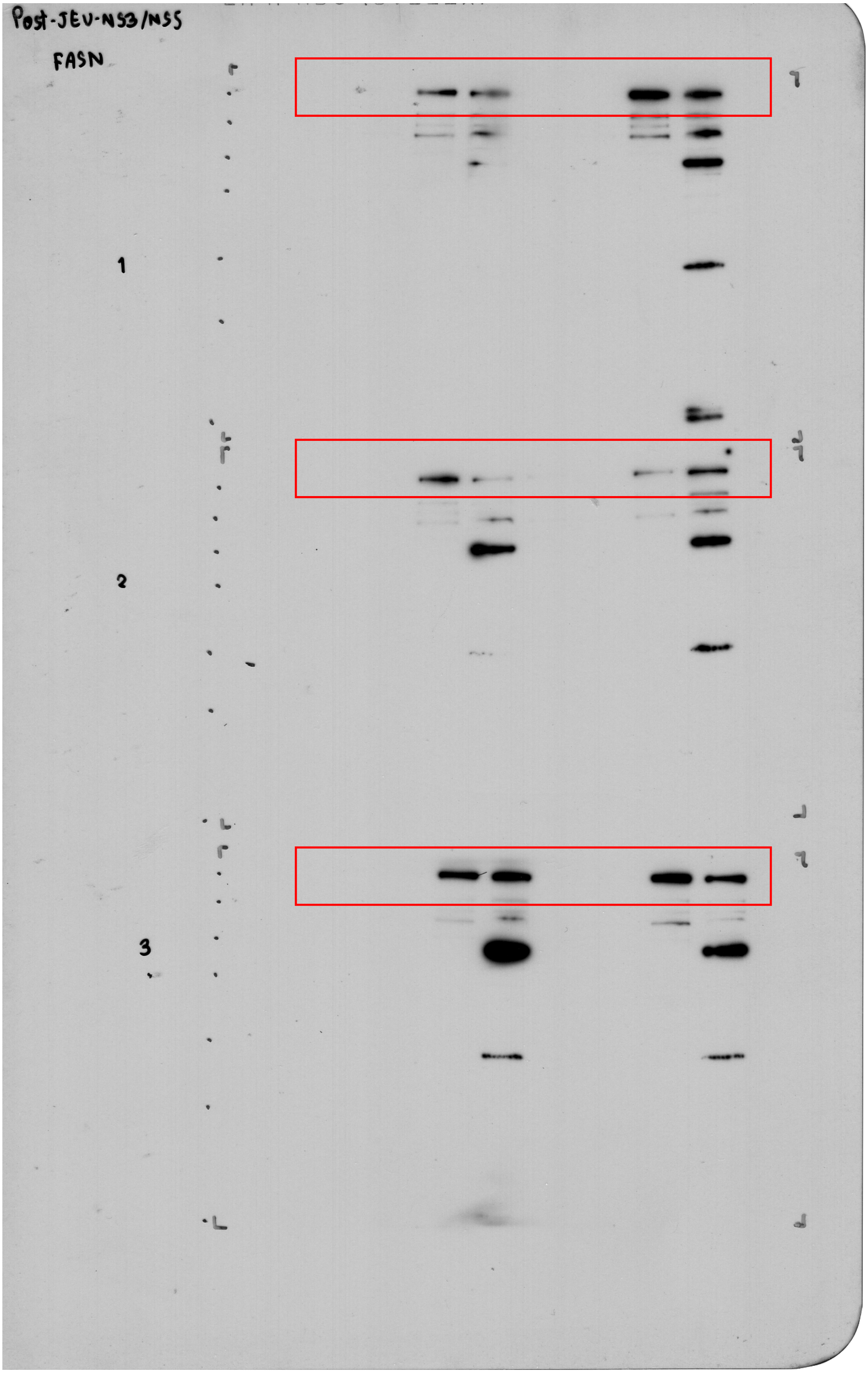

Figure 2E ; JEV_FASN (lysate) #1,2

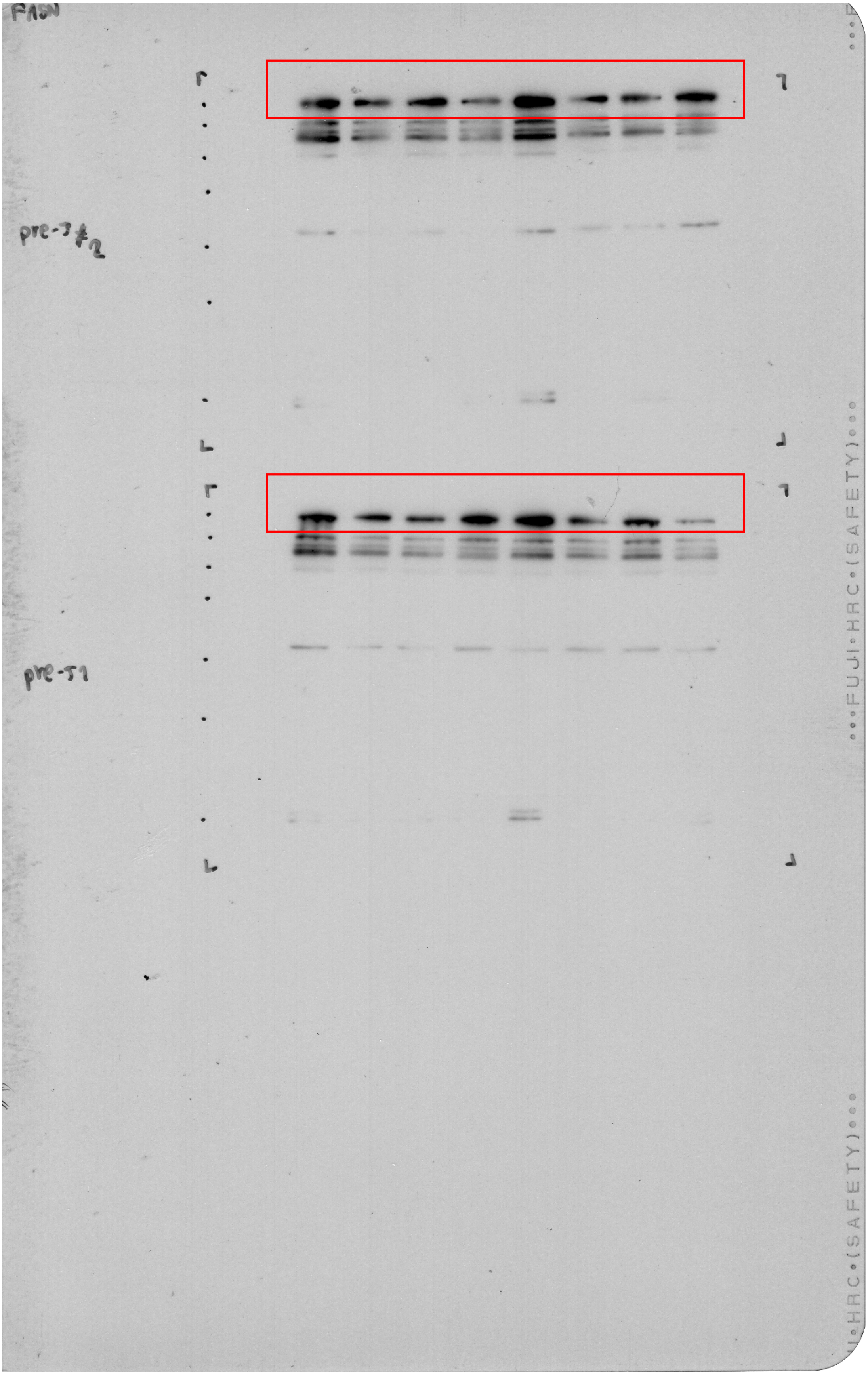

Figure 2E ; JEV_FASN (lysate) #3

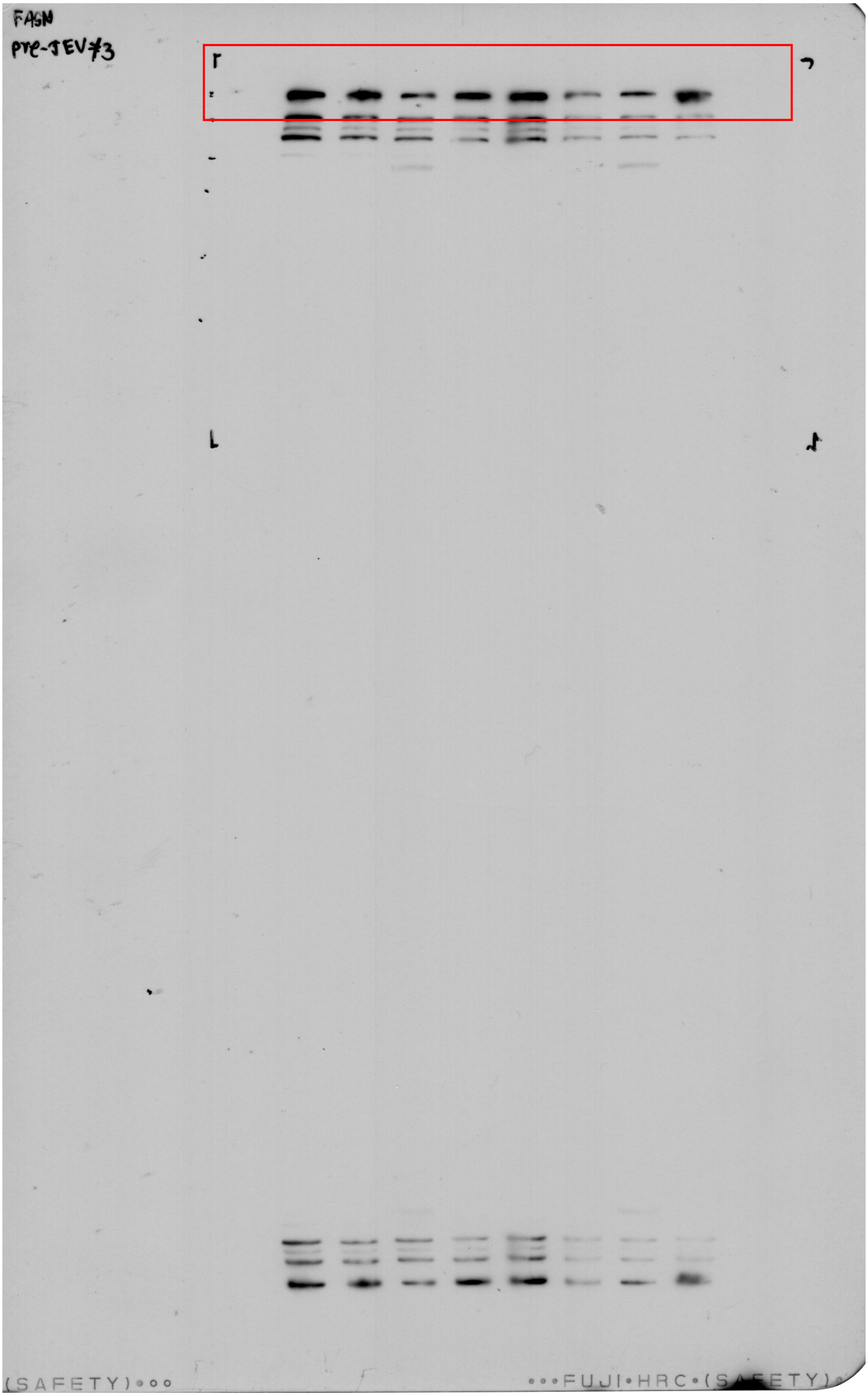

Figure 2E ; JEV_GFP (IP) #1, 2, 3

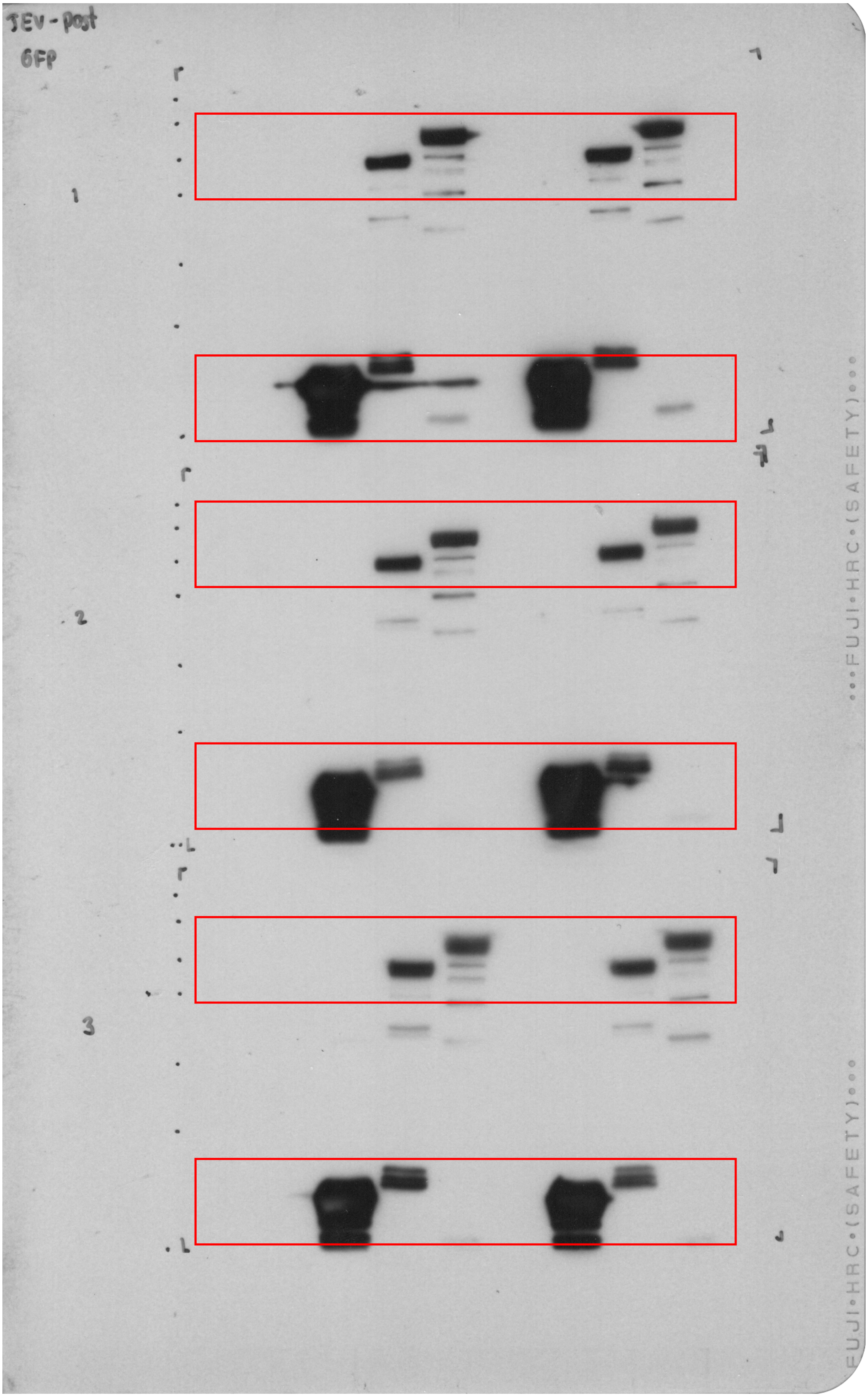

Figure 2E ; JEV_GFP (lysate) #1, 2, 3

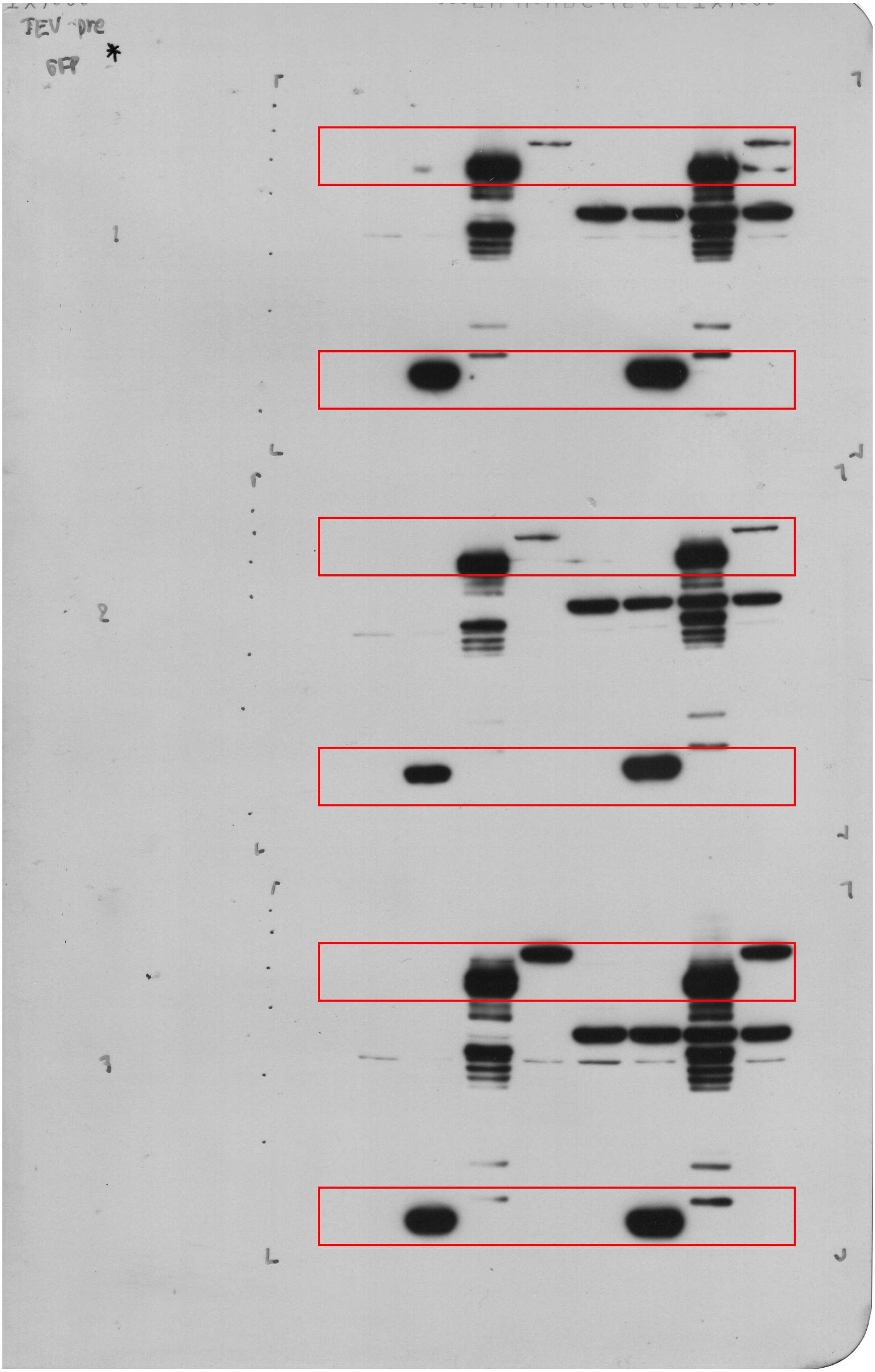

Figure 2E ; JEV_NS3 (lysate) #1, 2, 3

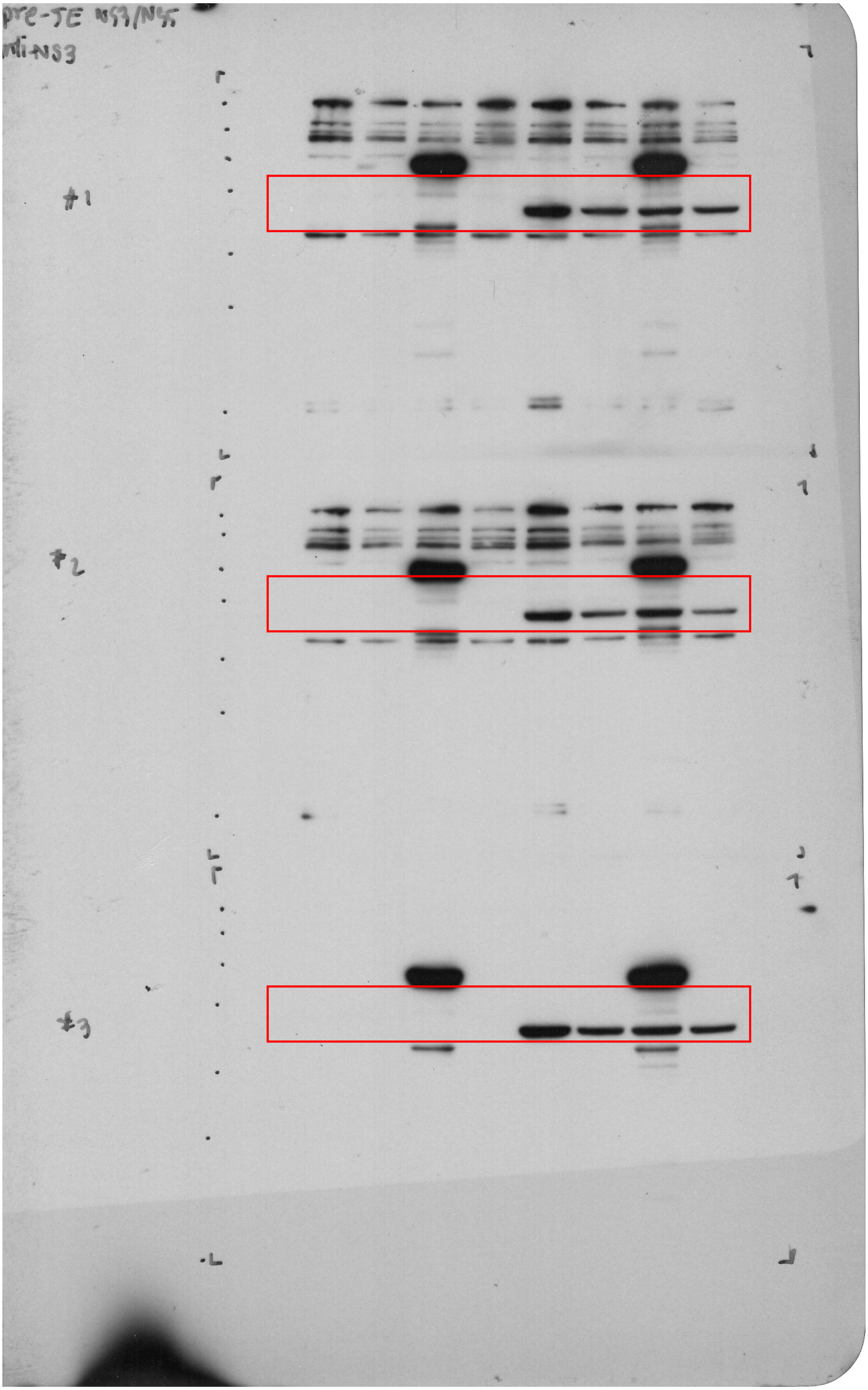

Figure 2E ; JEV_GAPDH (lysate) #1, 2, 3

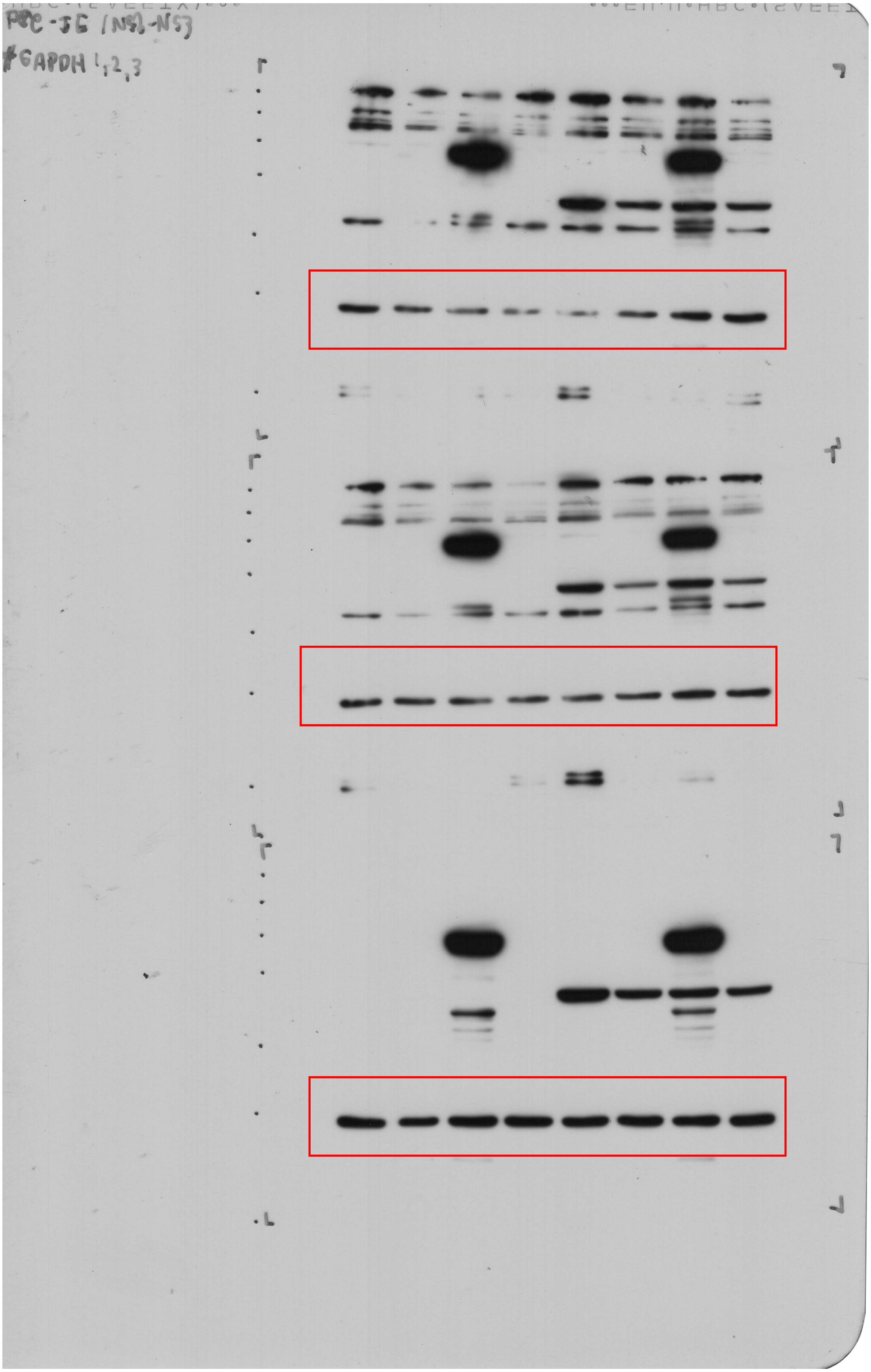

Figure 7A, C ; ZIKV, DENV_FASN

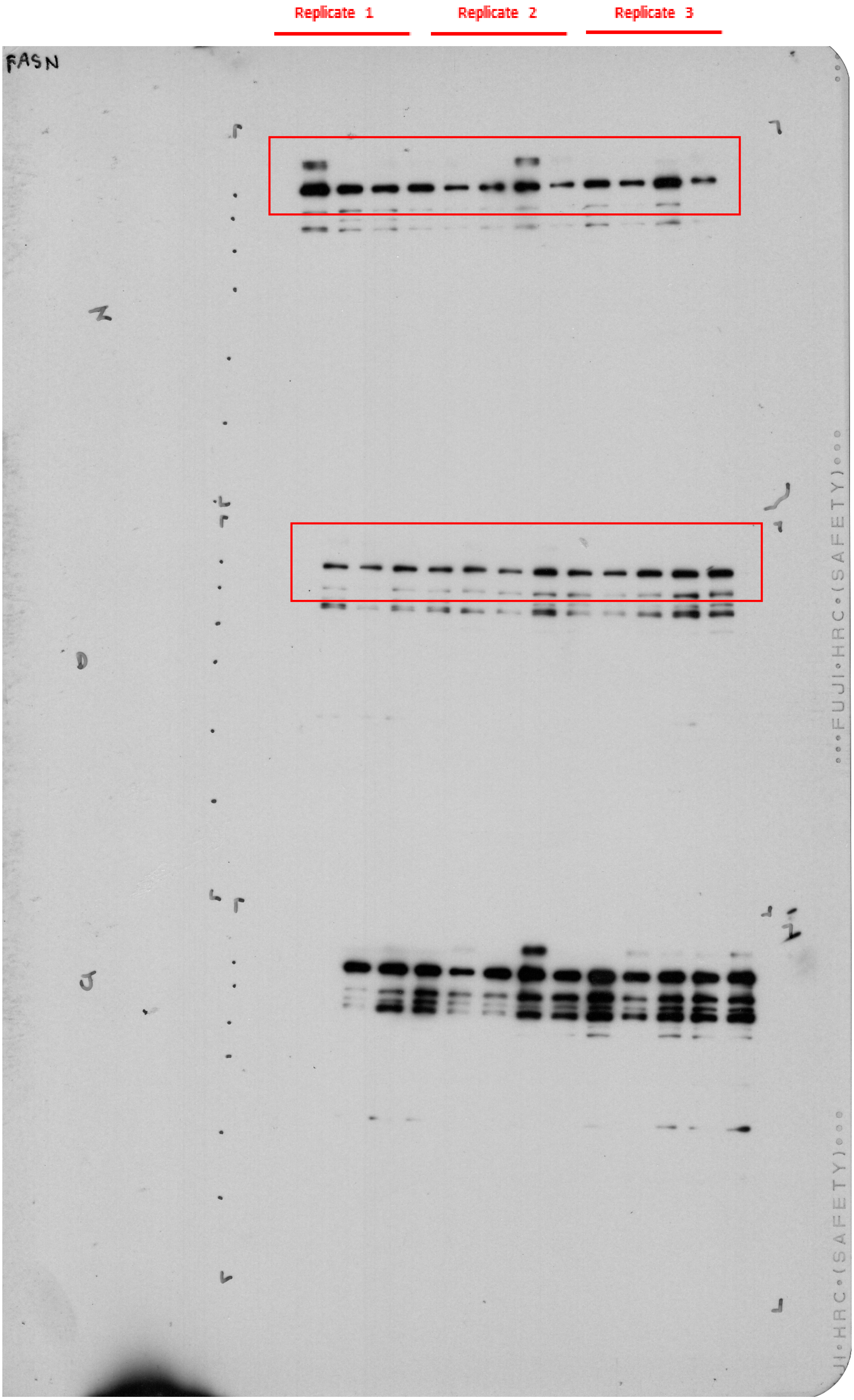

Figure 7E; JEV_FASN

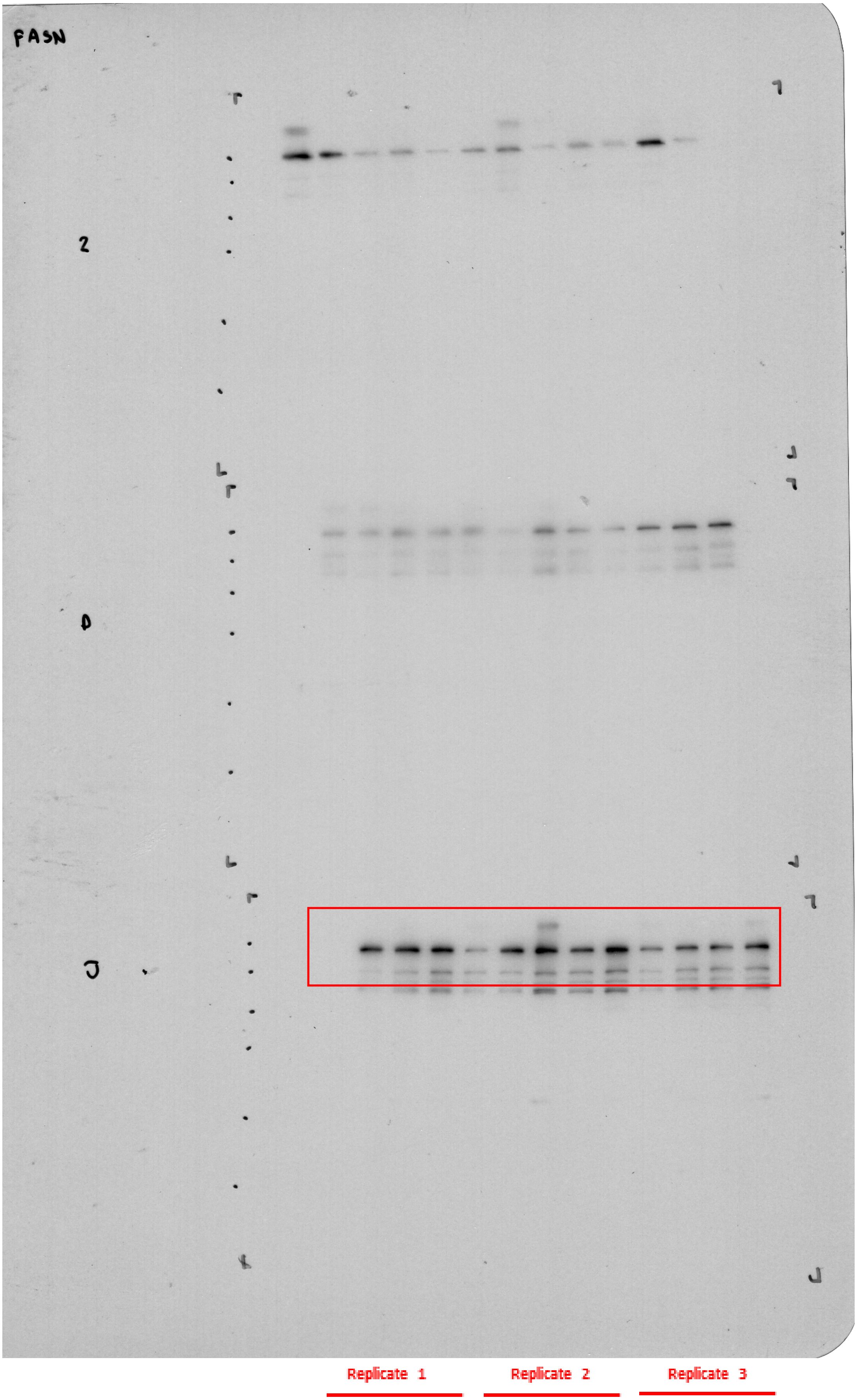

Figure 7A ; DENV_GAPDH

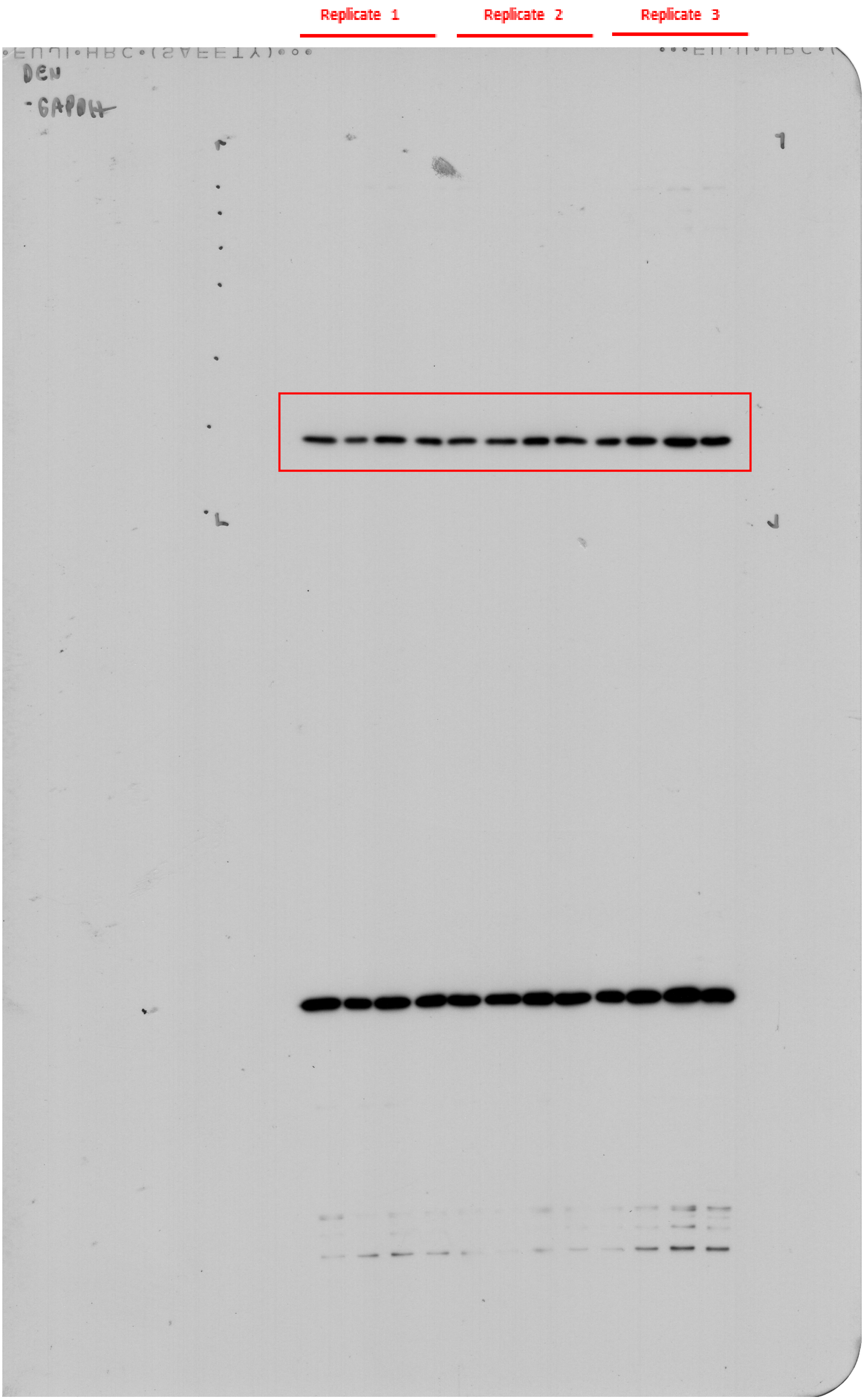

Figure 7C, E ; ZIKV, JEV_GAPDH

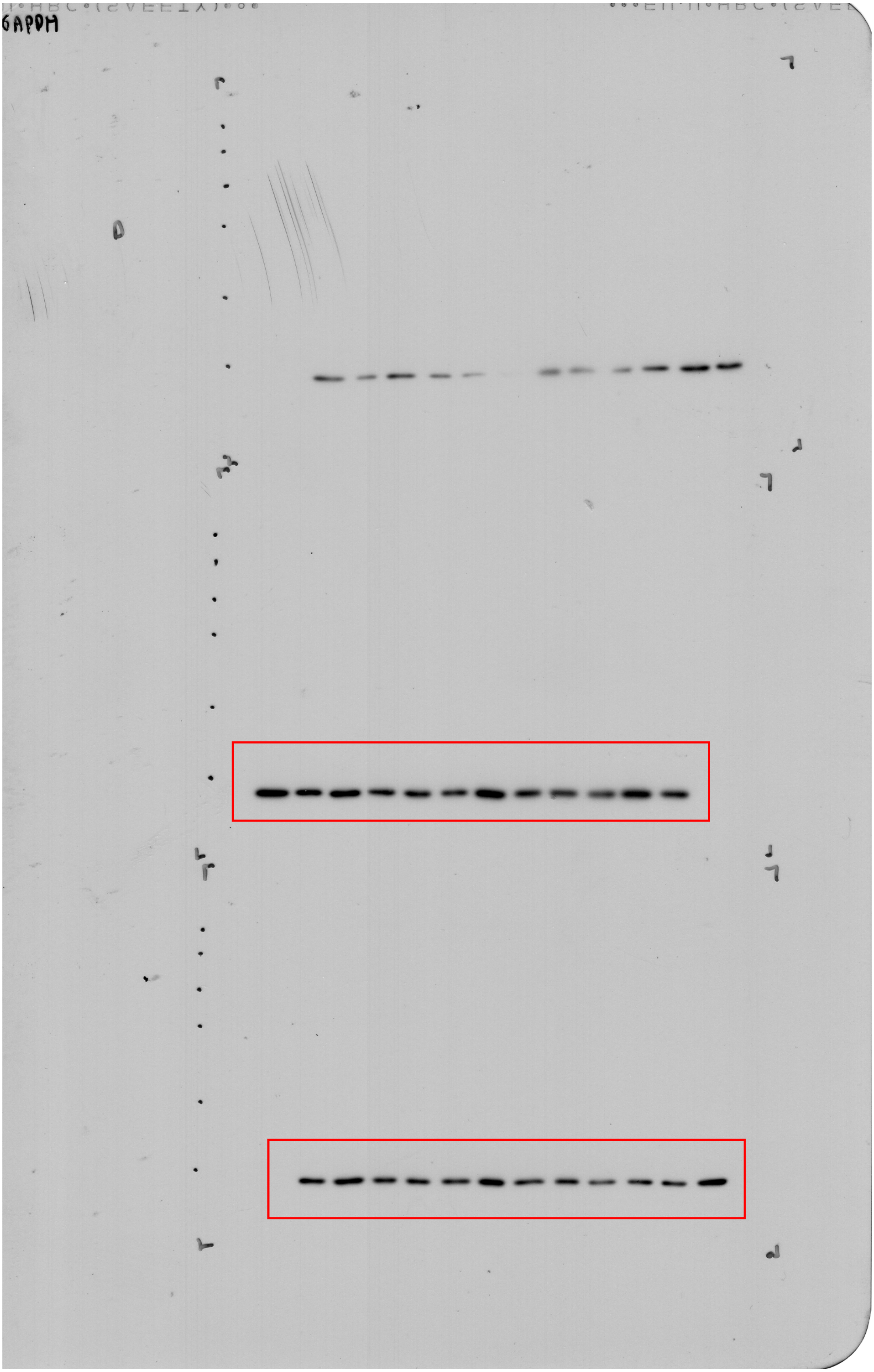

Replicate 1 Replicate 2 Replicate 3

Figure 7A ; DENV_ENV

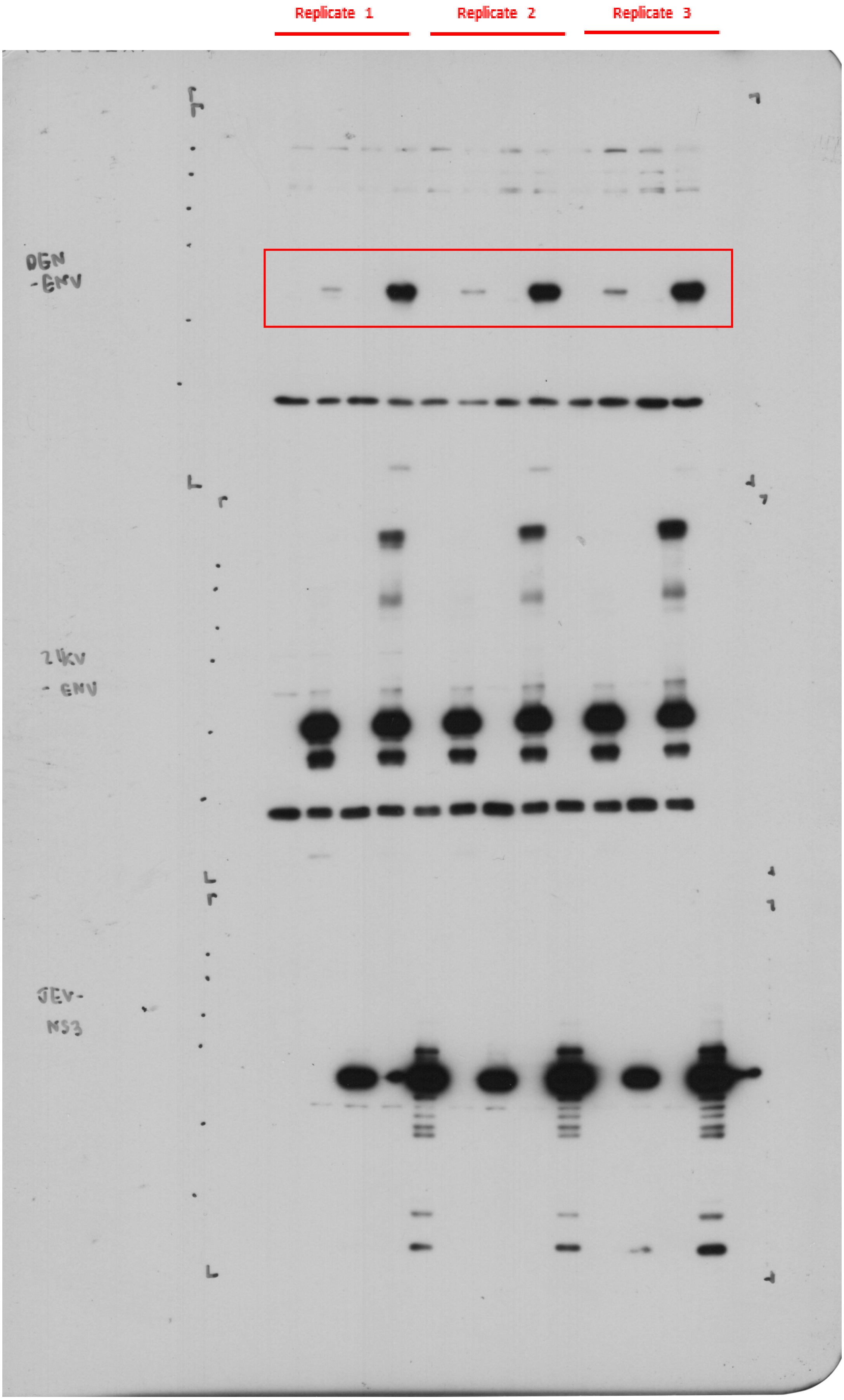

Figure 7C ; ZIKV_ENV

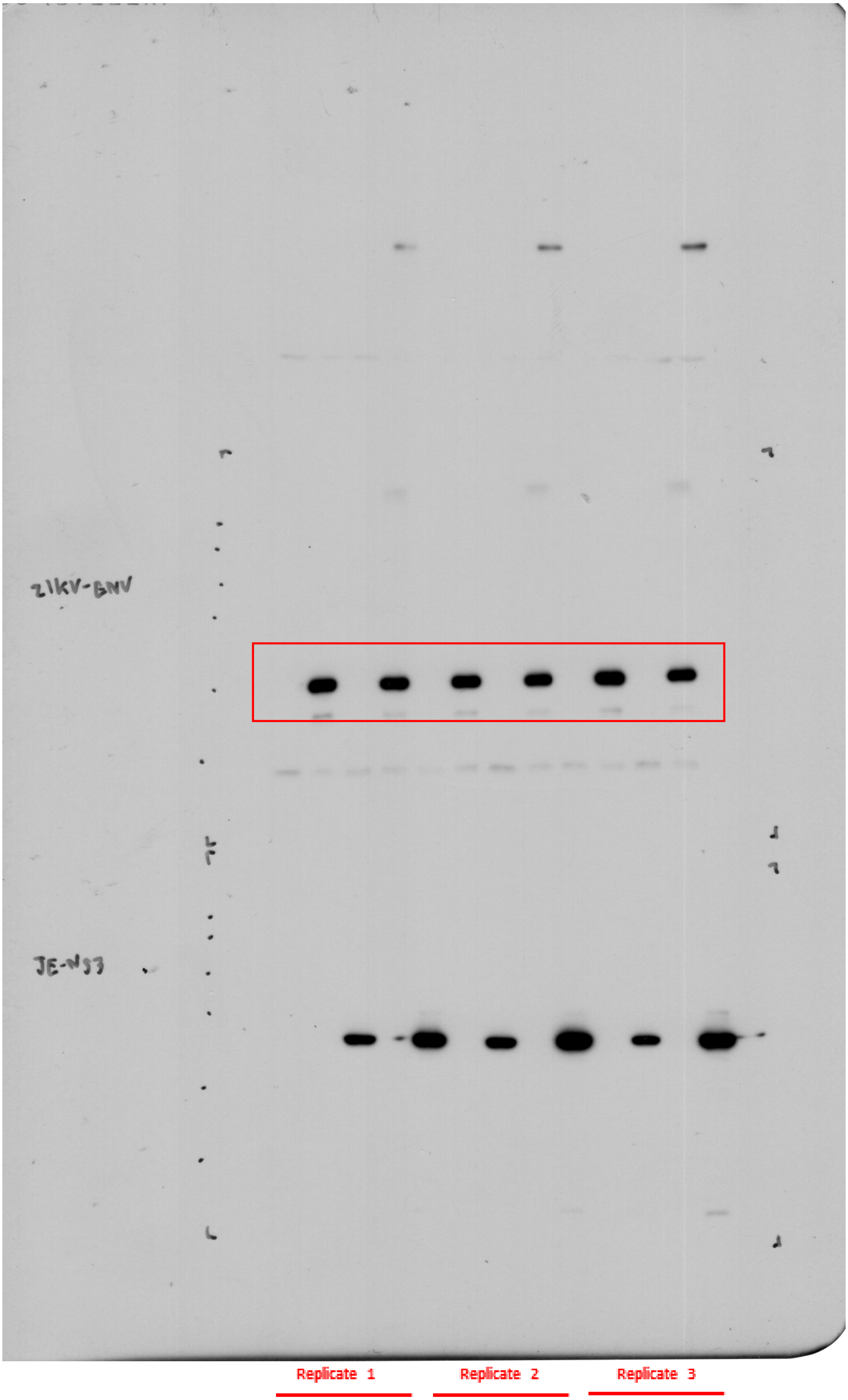

Figure 7E ; JEV_NS3

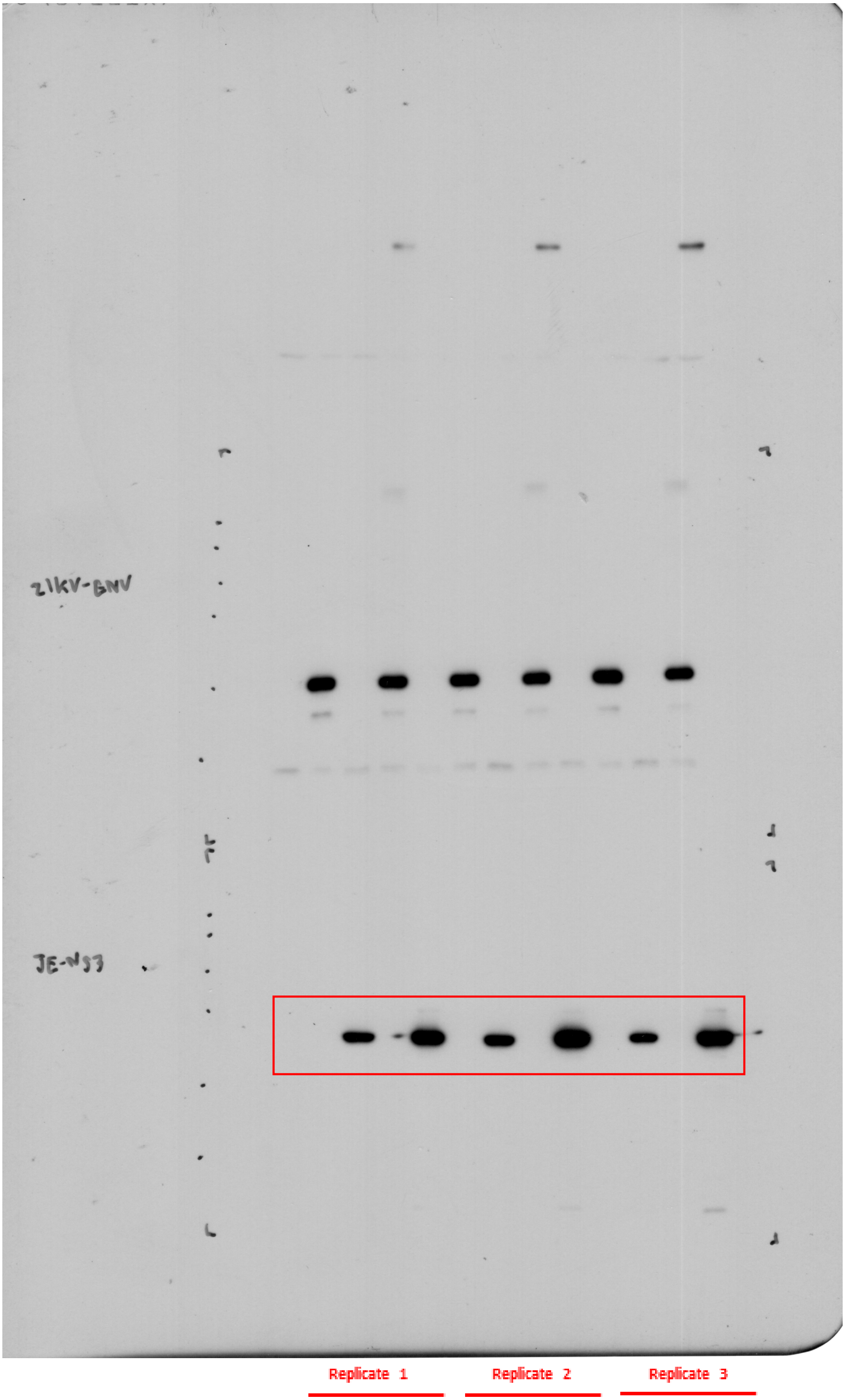

Supplement: S1 File — S1 Fig. The three-dimensional structure of HEK239/17 and A549 cells infected with DENV, JEV, and ZIKV at 12 and 24 hpi. Confocal microscopy determined the colocalization between FASN and NS3/dsRNA of (A) DENV (B) ZIKV (C) JEV. The images were taken in approximately 30 stacks, with 63X magnification and 1.5X Zoom. Each color represents different fluorochrome staining proteins. For staining of FASN and NS3, FASN is represented in green (AlexaTM Fluor 488), and NS3 is represented in red (AlexaTM Fluor 647). For staining of FASN and dsRNA, FASN is represented in red (AlexaTM Fluor 647), and NS3 is represented in green (AlexaTM Fluor 488). The nucleus is represented in blue (DAPI). The percent colocalization was analyzed by Imaris program (version 9.9.0) shown in the right panels. S1 Table. Primer sequences for constructing NS3 and FASN. uncropped western blots. (PDF) [file pone.0319207.s001.pdf]
